# The Condition-Dependent Transcriptional Landscape of *Burkholderia pseudomallei*

**DOI:** 10.1371/journal.pgen.1003795

**Published:** 2013-09-12

**Authors:** Wen Fong Ooi, Catherine Ong, Tannistha Nandi, Jason F. Kreisberg, Hui Hoon Chua, Guangwen Sun, Yahua Chen, Claudia Mueller, Laura Conejero, Majid Eshaghi, Roy Moh Lik Ang, Jianhua Liu, Bruno W. Sobral, Sunee Korbsrisate, Yunn Hwen Gan, Richard W. Titball, Gregory J. Bancroft, Eric Valade, Patrick Tan

**Affiliations:** 1 Genome Institute of Singapore, Singapore, Republic of Singapore; 2DMERI@DSO, DSO National Laboratories, Singapore, Republic of Singapore; 3 School of Applied Science, Republic Polytechnic, Singapore, Republic of Singapore; 4 Department of Biochemistry, Yong Loo Lin School of Medicine, National University of Singapore, Singapore, Republic of Singapore; 5 College of Life and Environmental Sciences, University of Exeter, Exeter, United Kingdom; 6 Department of Immunology and Infection, Faculty of Infectious & Tropical Diseases, London School of Hygiene & Tropical Medicine, London, United Kingdom; 7 Virginia Bioinformatics Institute at Virginia Tech, Blacksburg, Virginia, United States of America; 8 Department of Immunology, Faculty of Medicine Siriraj Hospital, Mahidol University, Bangkok, Thailand; 9 Institut de Recherche Biomédicale des Armées/CRSSA, La Tronche, France; 10 Ecole du Val-de-Grâce, Paris, France; 11 Duke-NUS Graduate Medical School, Singapore, Republic of Singapore; 12 Cancer Science Institute of Singapore, National University of Singapore, Singapore, Republic of Singapore; The University of Texas Health Science Center at Houston, United States of America

## Abstract

*Burkholderia pseudomallei* (Bp), the causative agent of the often-deadly infectious disease melioidosis, contains one of the largest prokaryotic genomes sequenced to date, at 7.2 Mb with two large circular chromosomes (1 and 2). To comprehensively delineate the Bp transcriptome, we integrated whole-genome tiling array expression data of Bp exposed to >80 diverse physical, chemical, and biological conditions. Our results provide direct experimental support for the strand-specific expression of 5,467 Sanger protein-coding genes, 1,041 operons, and 766 non-coding RNAs. A large proportion of these transcripts displayed condition-dependent expression, consistent with them playing functional roles. The two Bp chromosomes exhibited dramatically different transcriptional landscapes — Chr 1 genes were highly and constitutively expressed, while Chr 2 genes exhibited mosaic expression where distinct subsets were expressed in a strongly condition-dependent manner. We identified dozens of *cis*-regulatory motifs associated with specific condition-dependent expression programs, and used the condition compendium to elucidate key biological processes associated with two complex pathogen phenotypes — quorum sensing and *in vivo* infection. Our results demonstrate the utility of a Bp condition-compendium as a community resource for biological discovery. Moreover, the observation that significant portions of the Bp virulence machinery can be activated by specific *in vitro* cues provides insights into Bp's capacity as an “accidental pathogen”, where genetic pathways used by the bacterium to survive in environmental niches may have also facilitated its ability to colonize human hosts.

## Author Summary

Bacterial transcriptomes are dynamic, context-specific and condition-dependent. Infection by the soil bacterium, *Burkholderia pseudomallei*, causes melioidosis, an often fatal infectious disease of humans and animals. Possessing a large multi-chromosomal genome, *B. pseudomallei* is able to persist and survive in a multitude of environments. To obtain a comprehensive overview of *B. pseudomallei* expressed transcripts, we initiated whole-genome transcriptome profiling covering a broad spectrum of conditions and exposures — a so-called “condition compendium”. Using the compendium, we confirmed many previously-annotated genes and operons, and also identified hundreds of novel transcripts including anti-sense transcripts and non-coding RNAs. By systematically examining genes exhibiting highly similar expression patterns, we ascribed putative functions to previously uncharacterized genes, and identified novel regulatory elements controlling these expression patterns. We also used the compendium to elucidate candidate virulence pathways associated with quorum-sensing and infection in mice. Our study showcases the power of a *B. pseudomallei* condition compendium as a valuable resource for understanding microbial physiology and the pathogenesis of melioidosis.

## Introduction

A central goal of pathogen genomics involves identifying the complete repertoire of functional genetic elements within pathogen genomes, including protein-coding genes, *cis*-regulatory elements, and non-coding RNAs, and understanding how these elements operate to cause clinical disease. Analysis of >7,000 prokaryotic genomes in the PAThosystems Resource Integration Center (PATRIC, www.patricbrc.org) has revealed striking diversity in microbial genome sizes [1], the existence of prokaryotes with either single or multiple chromosomes [2], and evolutionary conservation of virulence pathways [3]. Besides genome analysis, transcriptomic profiling of microbial pathogens has also proved invaluable for validating computationally predicted genes and highlighting novel genes missed by computational algorithms based on DNA-sequence alone. Identifying genes expressed under specific conditions can also often provide important clues regarding gene function [4], [5]. However, unlike bacterial genomes that are mostly static, transcriptomes are dynamic, context-specific and condition-dependent. As such, achieving a comprehensive overview of expressed transcripts for any bacterial species ideally requires a detailed collection of profiles covering a broad spectrum of conditions and exposures – a so-called “condition compendium”. While condition compendia for a few bacteria (e.g. *Mycoplasma pneumoniae*, *Bacillus subtilis*) have been reported [6], [7], previous studies have been limited to microbes with small sized genomes and single chromosomes. There is thus a need for similarly detailed transcriptomic studies of bacterial species with large, multi-chromosomal genomes.

The Gram-negative bacterium *Burkholderia pseudomallei* (Bp) is the causative agent of melioidosis, a tropical infectious disease of humans and animals. Among sequenced microbial genomes, the Bp genome is large (7.2 Mb), composed of two chromosomes (Chr 1 and 2) [8], and predicted by sequence analysis to contain ∼5,900 protein coding genes [8]. Human melioidosis has a high mortality rate, estimated at 20% in Northern Australia and up to 50% in Northeast Thailand [9]. Underscoring its highly infective nature, Bp has been categorized as a Tier 1 disease agent under the US Federal Select Agent Program [10]. Bp has a striking ability to survive and thrive in a multiplicity of environments. In endemic areas, the bacterium can be cultured from various sources including soil, water and air, and it can infect a wide range of hosts such as amoebae, nematodes, plants, land and sea mammals, and plants [11]. This versatility suggests that Bp could prove useful as a model to study how pathogens adapt to extreme environments and different hosts. Indeed, it has been proposed that Bp is an example of “accidental virulence”, where genetic pathways used by the bacterium to survive in environmental niches may have indirectly contributed to its ability to cause clinical disease [12].

In this study, we sought to obtain a global overview of how the environment might influence the Bp transcriptional landscape, by integrating expression data from whole-genome tiling microarrays covering >80 diverse environmental and genetic conditions. Our aims in this study were threefold: First, we generated a comprehensive strand-specific catalog of condition-dependent transcripts in Bp, including genes, operons, and non-coding RNAs. Second, we explored if the two Bp chromosomes might be associated with distinct patterns of transcription, related to their overall functions. Third, we defined *cis*-regulatory motifs associated with condition-dependent expression programs, and applied the compendium to elucidate candidate virulence pathways associated with quorum-sensing and *in vivo* infection. Taken collectively, the condition-dependent expression compendium represents a valuable resource for understanding Bp physiology and the pathogenesis of melioidosis. Moreover, our findings may also prove applicable to other bacterial pathogens with multiple chromosomes.

## Results

### Genomic Landscape of the Bp Condition-Dependent Transcriptome

**Figure 1:**
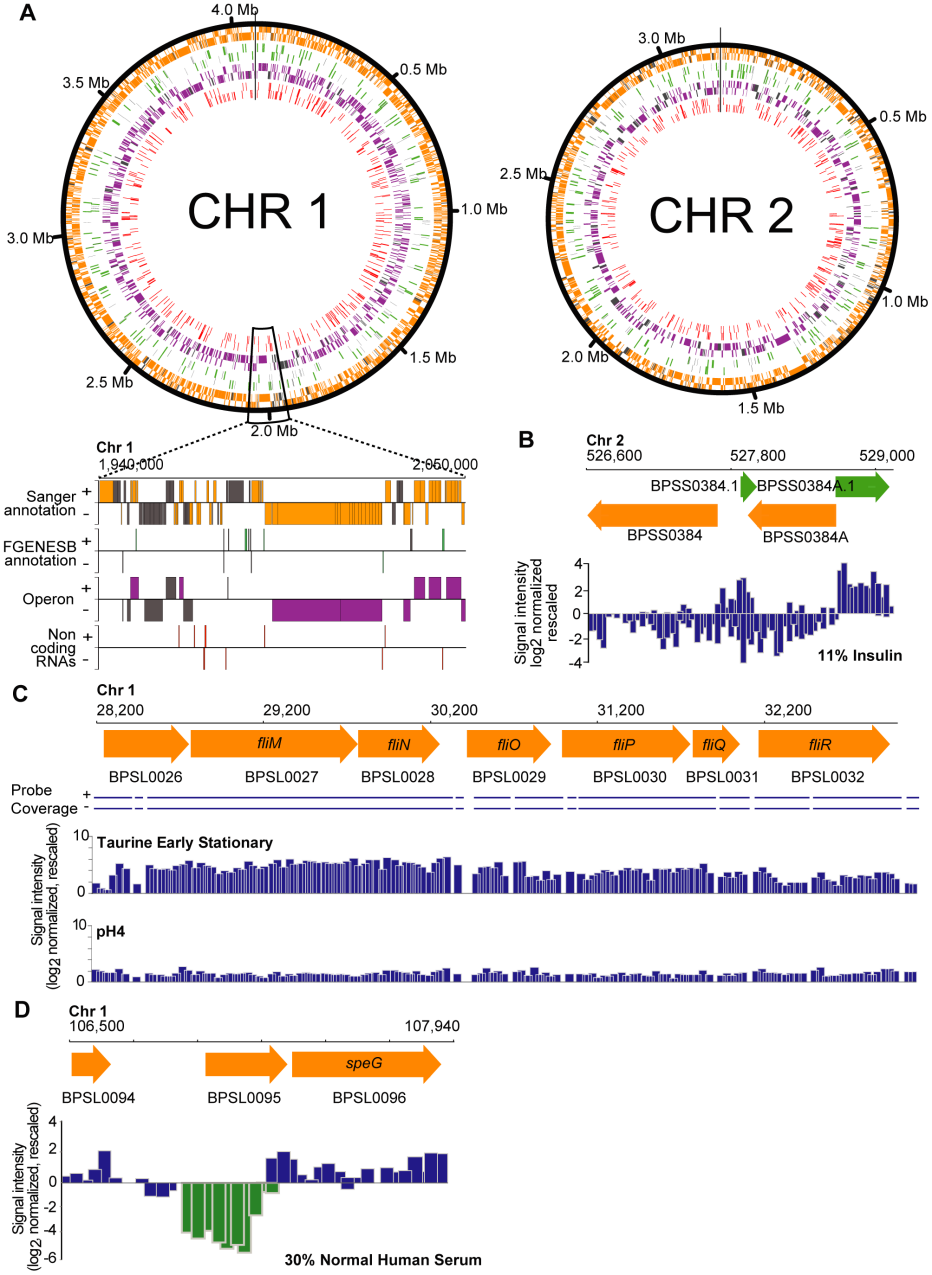
Expressed transcripts in the Bp condition compendium. High-resolution views of different genomic features are depicted. All transcripts depicted were expressed above the median cut-off threshold. (A) Transcriptional annotation of the *Burkholderia pseudomallei* K96243 reference genome. The transcriptome map is presented along the chromosomal coordinates in a strand-specific manner, with the outermost track composed of Sanger annotated genes (orange), followed by novel genes (green), the Bp operons (purple) and finally the non-coding RNAs (ncRNAs; red). In all tracks, predicted genomic features that do not have an associated transcript in this study are colored in grey. The genes, operons and ncRNAs are arranged in a strand-specific manner by visualizing them in either the forward (+) or the reverse (−) tracks. The black vertical lines indicate the start/stop sites of the circular chromosomes. (B) Sanger genes and novel genes. Expressed strand-specific transcripts are presented as blue bars along the forward and reverse strands. Transcript boundaries correspond to predicted start and stop coordinates of Sanger annotated genes and FGENESB novel genes. (C) Differential expression of a Bp operon. Expression of a predicted flagella operon (*BPSL0026 – BPSL0032*) in a specific condition (taurine exposure). (D) Antisense transcription. *BPSL0095*, a gene coding for hypothetical protein exhibits antisense transcription upon exposure to human serum.

Whole-genome tiling microarrays containing strand-specific probes overlapping at 35-base resolution were used to profile Bp transcriptional responses under 82 different conditions (Figure S1A–C). Conditions were selected to mimic natural exposures Bp might encounter in the environment or in infected hosts. Many of these conditions were selected based on prior scientific reports where Bp responses were explored at the phenotypic level. Experimental conditions and their scientific rationales are provided in Table S1. The transcription profiles were found to be robust and reproducible through technical and biological replicates [13] (Figure S1D). We integrated the array data to generate a comprehensive catalog of condition-dependent transcripts in Bp. Using a sliding window smoothing algorithm [14], we identified 5,616 transcriptionally active regions (TARs) across the 82 conditions (Table S2), ranging in size from 215 bp to 52,724 bp (median length 752 bp). We systematically annotated the TARs by comparing them to a variety of genomic features, including “gold standard” Sanger genes [8], novel genes predicted by FGENESB, a separate gene prediction software [13], [15], operons, antisense transcripts, and genomic islands (GIs) (Figure 1). We validated several of these findings using RT-PCR (Figure S2, Table S3). An annotated file describing these transcripts is presented in Table S2, and also in the PATRIC online resource platform (www.patricbrc.org).

#### Genes

We confirmed detectible expression of 5,467 out of 5,935 Sanger genes (92.1%) (Figure 1A, Table S2). Interestingly, 468 Sanger genes did not exhibit detectible expression throughout the Bp condition compendium. These included specific genes residing in Type III and Type VI secretion clusters (T3SS1, T3SS2, T6SS-1 and T6SS-5), genes regulating capsule formation (CPS IV), and certain genes in genomic islands (GIs) (Table S4). The lack of expression of these genes may either indicate the absence of an appropriate condition required for triggering expression of these genes – For example, some T3SS1 and T3SS2 genes might only be expressed during plant infection [16], or alternatively some of these “silent” genes may represent mis-annotated or non-functional genes. Supporting this latter hypothesis, a significant proportion of these “silent” genes encoded hypothetical proteins (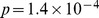, Text S1) or genes not conserved in other Bp strains (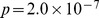).

Besides Sanger genes, we also recently reported the existence of >500 putative novel genes not annotated in the original reference genome (see Discussion) [13]. Of these, 306 novel genes (59.1%) were associated with expressed transcripts (Figure 1B, Table S2). Notably, more than half of the novel genes were expressed in very specific sets of conditions (Table S5) – for example, *BPSL0061.1*, encoding a short 31 aa predicted protein, was only detectably expressed in anaerobic conditions, during macrophage infection, and in quorum sensing mutants (Table S2). These results suggest that many novel genes are likely to demonstrate condition-specific expression.

#### Operons

Of 1,249 computationally predicted polycistronic operons in BpK96243 [13], we detected expression of 1,041 operons (Table S2). ∼20% of the operons (201/1041) were constitutively expressed (≥70 conditions), and often associated with core cellular functions, including DNA replication (*BPSL0073 – BPSL0075*), protein-folding (*BPSL2697-BPSL2698*) and global transcriptional regulation (*BPSL1502 – BPSL1506*; containing *rpoS*) (Table S2, S5). In contrast, condition-specific operons were often involved in accessory pathways such as phosphonate transport (*BPSL2851 – BPSL2857*; expressed upon long-term heat stress), two component sensing (*BPSS1039 – BPSS1043*, expressed upon zinc exposure) and flagella motility (*BPSL0026 – BPSL0032*; expressed upon cold stress and taurine exposure) (Figure 1C).

#### Antisense transcription

Prokaryotic antisense transcription is emerging as an important mechanism regulating many processes including stress response and virulence [17]. To explore antisense transcription in Bp, we defined an antisense transcript as TAR associated strictly with the opposite strand of a Sanger gene, either partially or throughout the entire gene (Figure 1D). Using these criteria, we observed antisense transcription events for 10% of Sanger genes. The occurrence of an antisense transcript was not necessarily associated with cognate expression on the sense strand. Antisense transcription was also observed for whole operons (Figure S3).

#### Genomic Islands

Genomic Islands (GIs) are regions in a bacterial genome representing horizontal transfer events [8]. 16 GIs have been identified in the BpK96243 genome (Table S2). Analyzing BpK96243-specific profiles, we found that genes in GIs were expressed at significantly lower levels compared to other expressed genes on the same chromosomes (Chr 1: 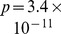; Chr 2: 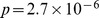, Wilcoxon signed rank test). Several GIs exhibited signatures of condition-dependence (Figure S4). For example, GI14 genes were expressed only under nutrient-deprived conditions or in taurine/sulphur – GI14 contains *BPSS0665*, a *tauD* gene homolog involved in taurine metabolism [13]. We also observed condition-dependent expression of genes in GI1, GI3, GI12 and GI15 upon antibiotic stress (ceftazidime and chloramphenicol) - largely comprising bacteriophage-related genes. The observation that many GI genes are expressed in a condition-dependent manner suggests that they may play a role in the phenotypic diversity of Bp, contributing to survival in specific niches.

### Abundance of Condition-Dependent Non-coding RNAs in Bp

**Figure 2:**
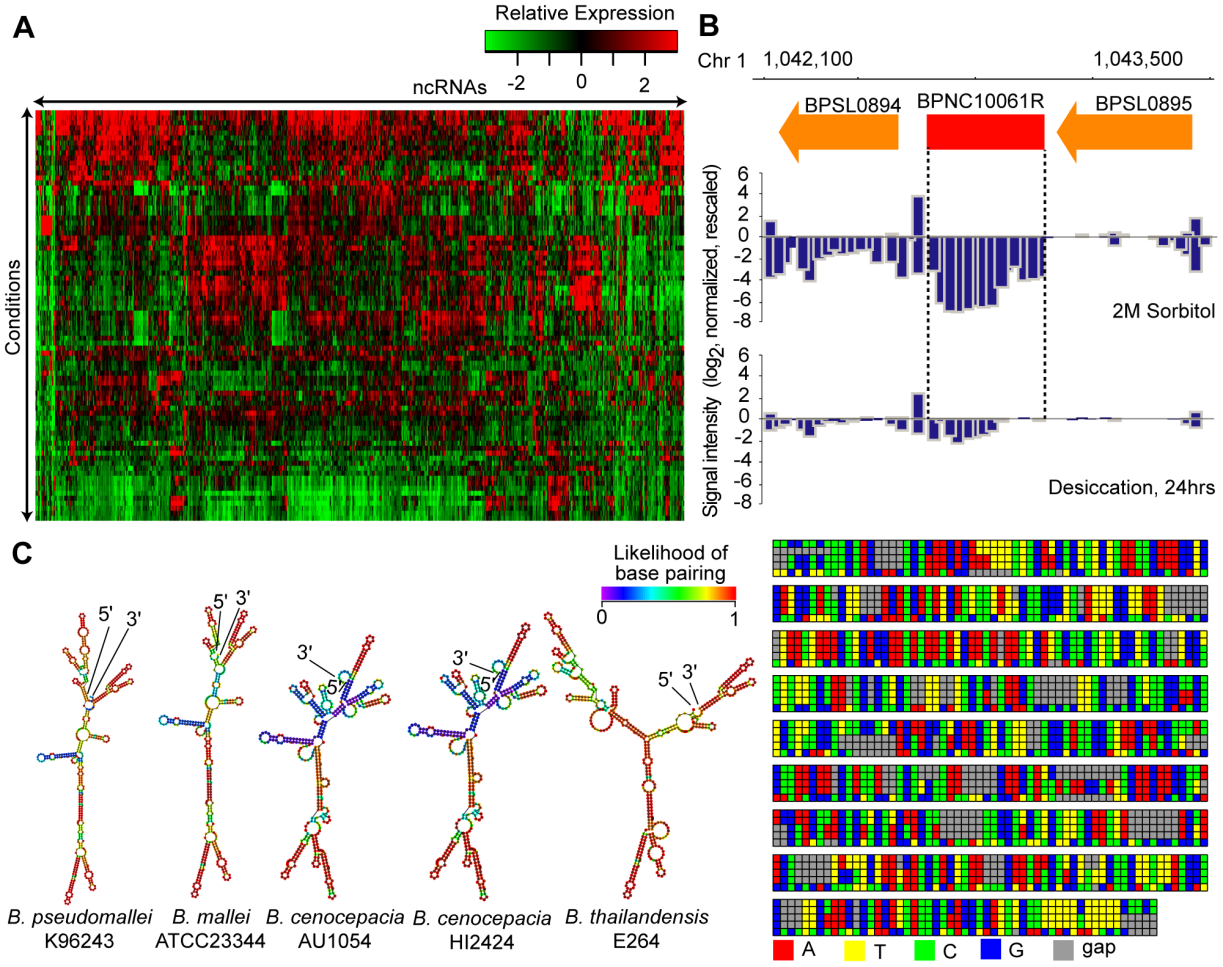
Identification of Bp ncRNAs. (A) Condition-dependence of ncRNA expression. The heat-map depicts 766 identified ncRNAs and their patterns of expression across the condition compendium. Red depicts high expression, and green depicts low expression. (B) *BPNC10061R* expression is triggered by sorbitol. *BPNC10061R* is highly expressed under condition of osmotic stress (2M Sorbitol) compared to desiccation. (C) Secondary structure and species conservation of *BPNC10061R*. Consensus sequences homologous to *BPNC10061R* are found in *B. mallei*, *B. cenocepacia* and *B. thailandensis* strains. The sequences were aligned, and corresponding secondary structures were predicted.

Non-coding RNAs (ncRNAs) are emerging as an important class of regulatory molecules in several prokaryotes [18]. Using stringent filtering criteria and manual curation (see Materials and Methods), we identified a “high-confidence” set of 766 ncRNA transcripts ranging in size from 111 to 750 bp exhibiting high expression levels in the Bp compendium (Figure 2A, Table S2, Text S1). All 81 ncRNAs computationally predicted by the ncRNA database Rfam to in the BpK96243 genome were detectibly expressed [18]. Of the 766 ncRNAs, 532 and 150 ncRNAs were conserved in *B. mallei* (Bm) and *B. thailandensis* (Bt) respectively, at both the levels of sequence identity and chromosomal synteny (Figure S5A,B).

On average, 168 ncRNAs were expressed in any single condition (Table S5). Many ncRNAs exhibited differential expression under different conditions (Figure S5C,D). For example, *BPNC10070F* was up-regulated 12-fold in nutrient-limiting conditions, and *BPNC10061R* exhibited high expression in high osmolarity and nutrient deprivation (Figure 2B, S2E). The Bp ncRNAs were associated with a variety of secondary structures (Table S6), consistent with them belonging to distinct functional classes. Evolutionary conservation analysis of *BPNC10061R* revealed highly homologous sequences in Bm, Bt and *B. cenocepacia* (Bc) but not *P. aeruginosa*. Interestingly, the predicted secondary structure of *BPNC10061R* is similar between Bp and Bm but distinct to Bt (Figure 2C). It is possible that *BPNC10061R*, while evolutionarily conserved within the *Burkholderia* genus, may play different functional roles in different *Burkholderia* species.

### Bp Chromosomes Exhibit Distinct Transcriptional Landscapes

**Figure 3:**
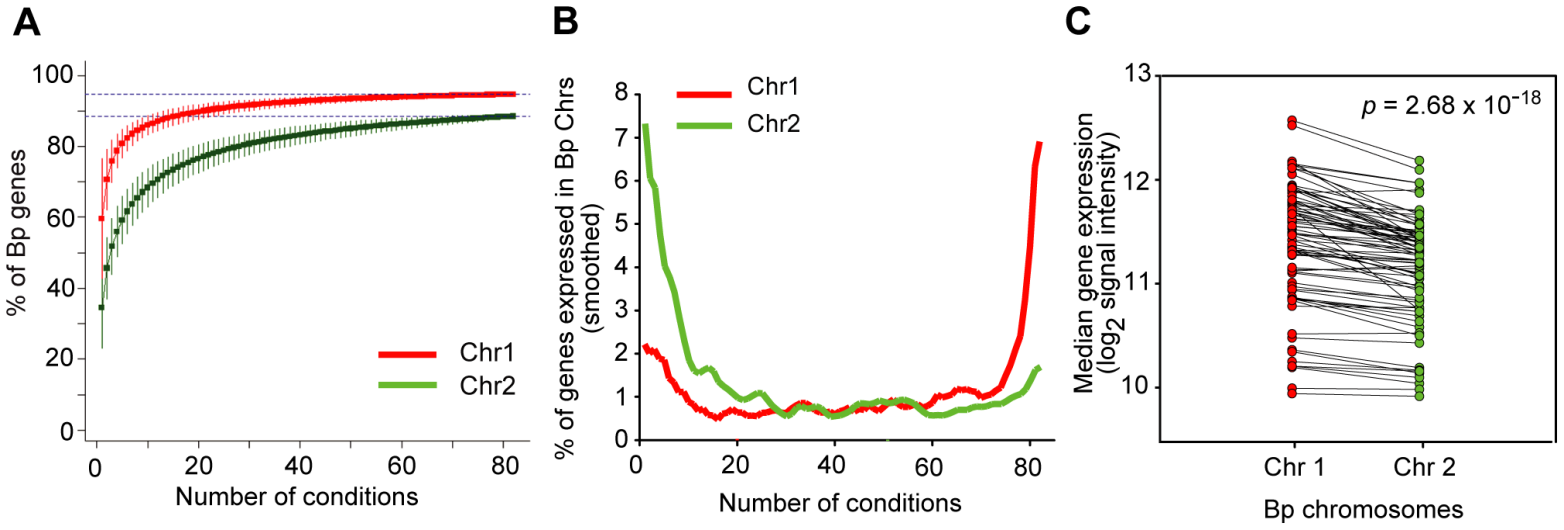
Bp chromosomes display distinct transcriptional landscapes. (A) Cumulative curves for expression of genes across the condition compendium. The graph represents the percentage of new genes expressed on Chr 1 (red) and Chr 2 (green) (y-axis) upon the successive addition of conditions (x-axis). This analysis was confined to Sanger genes to minimize annotation errors. (B) Chr 1 and Chr 2 exhibit constitutive and mosaic expression respectively. The graph relates the proportion of genes expressed on each chromosome (y-axis) under any particular number of conditions (x-axis). Chr 1 genes are expressed in most conditions (rightward upslope, red), while Chr 2 genes are expressed in specific conditions (leftward upslope, green). (C) Chr 1 genes exhibit higher expression levels than Chr 2 genes. Each dot represents the median expression of all detectably expressed genes on the respective chromosome, joined by the same condition. Chromosomal expression levels were compared using one-tailed paired t-test (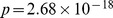).

Previous analysis has revealed that Bp Chr 1 is enriched in genes associated with core functions while Bp Chr 2 contains genes associated with accessory and secondary functions [8]. We investigated if there might exist systematic differences in the transcriptional landscapes of both chromosomes. When computed across all conditions, both Bp chromosomes exhibited a comparable proportion of expressed genes (94% of Chr 1 and 89% for Chr 2) (Figure 3A), suggesting that almost all Bp genes are expressed at least once in the Bp condition compendium. In contrast, dramatic differences in the transcriptional landscape of the two Bp chromosomes were observed when our analysis was confined to individual conditions. For any individual condition, the majority of Chr 1 genes (∼72%) were expressed, while only a minority of Chr 2 genes (∼28%) were expressed under any one condition (Figure 3B, Table S7). Chr 1 genes were also expressed at higher levels than Chr 2 genes (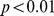, one-tailed paired t-test; Figure 3C, Table S7). This result suggests that genes on Bp Chr 1 are expressed in most or even all conditions, but Bp Chr 2 genes are highly regulated and only expressed under specific conditions, presumably when their gene products are required. Our results provide experimental support that despite >10 million years of coevolution [19] the two chromosomes in Bp continue to exhibit radically different transcriptional landscapes (Table S7).

### Network Analysis Defines Condition-Dependent Gene Expression Clusters

**Figure 4:**
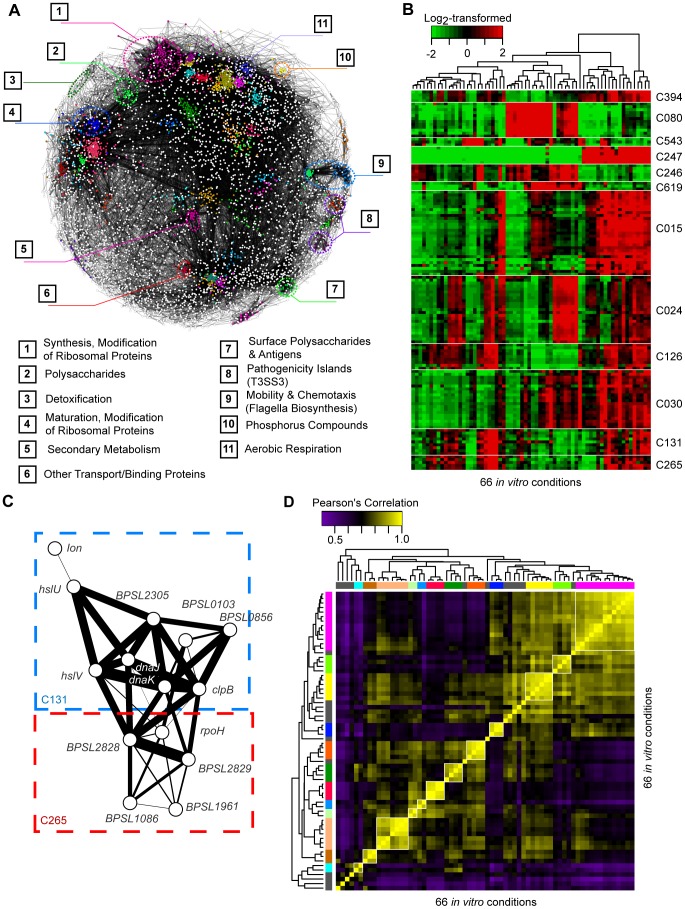
Co-expression network of Bp condition-dependent transcription. (A) Co-expression network. Nodes are individual genes, connected to one another by significant co-expression relationships (mutual information score 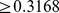). The colours represent clusters over-represented in different Riley annotations, and their respective annotations are provided at the bottom. (B) Condition dependent cluster expression. The heat-map depicts representative clusters and patterns of expression across conditions. Gene expression levels were mean-normalized. (C) Inter-cluster relationships. The MRCN unit M036 consists of two clusters: C131 and C265, which include genes encoding proteins for degrading misfolded proteins and other genes with hypothetical functions. Thickness of edges represents the strength of the co-expression relationship between two genes. (D) Condition groups. The different condition-specific transcriptional profiles were clustered to one another based on similarities in expression of genes from the Bp core genome. Condition groups deemed to be stable by bootstrap assessment are marked in colors.

We sought to define groups of genes (“clusters”) commonly co-expressed under different conditions, as co-expressed genes often share similar cellular functions [20]. Using the 66 profiles representing wild-type Bp exposed to well-defined experimental conditions, we identified co-expression relationships between genes in a hierarchical manner to assemble a Bp condition-dependent gene co-expression network (Figure 4A). Profiles corresponding to genetic mutants and *in vitro*/*in vivo* infection were not included, as these were subsequently used to validate and probe the network architecture (presented later). First, we used ARACNe, an information theoretic algorithm for biological network construction, to identify significantly co-expressed pairs of Bp genes [21]. ∼91% of Sanger genes exhibited significant co-expression relationships to at least one other gene (Figure 4A). Second, we used Markov Clustering [22] to group these linked genes into larger clusters, approximating a scale-free topology commonly associated with transcriptional networks (Text S1) [23]. After permutation testing (see Materials and Methods), we identified 470 highly reproducible clusters, containing 3,754 Sanger genes with a median of 4 genes per cluster. Third, to define higher-order relationships between clusters, we performed MRCN (maximum relatedness of clusters network) analysis to identify highly interlinked clusters [24]. We grouped 259 clusters into 98 MRCN units (average MRCN size = 3 clusters) (Table S8). In total, 55% of the Bp clusters mapped to predicted Bp operons, and one-third of the clusters were significantly enriched in at least one functional annotation (Figure 4A, Table S8). We also identified 363 ncRNAs to be significantly correlated with the clusters (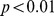), suggesting potential involvement of ncRNAs in these functions (Table S9).

The Bp gene clusters exhibited dynamic regulation across the 66 *in vitro* conditions (Figure 4B, Text S1). For example, clusters C394 (*arcDABC* operon), C247 (*narKGH* operon), and C126 (*paaABCDE* operon) were commonly overexpressed under conditions of temperature, ultra-violet exposure, and oxidative stress. Almost half (43/98) of the MRCNs comprised a mixture of functionally annotated and non-annotated clusters. For example, one MRCN highly expressed upon heat exposure comprised two clusters - C131, containing genes related to heat-stress (C131, 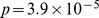), and C265, containing the heat-shock sigma factor *rpoH* and several hypothetical proteins (e.g. *BPSL1086*, *BPSL1961*, *BPSL2828* and *BPSL2829*) (Figure 4C). Interestingly, 5 MRCNs contained clusters associated with pathogen virulence genes, including pathogenicity islands (T3SS2 and T3SS3), chemotaxis and flagella, binding or transport proteins (T6SS3), and surface polysaccharides (Type I capsule) [25]. These virulence-related MRCNs, containing ∼35% of all putative virulence genes cataloged in the original Bp genome annotation [8], were expressed under conditions of nutrient deprivation and prolonged cold stress (4°C, 16 hours). These results thus suggest that specific *in vitro* cues may exist that can activate a substantial portion of the Bp virulence machinery.

Unsupervised clustering associating the different conditions to one another defined 12 robust condition groups encompassing 54 of the 66 condition-specific profiles (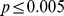
supported by bootstrap assessment, Figure 4D, Table S10). Conditions associated with rich media (LB) grouped together and segregated independently from conditions associated with minimal media (CDM), highlighting the profound influence of external nutrient conditions on the global Bp transcriptome. Interestingly, seemingly unrelated profiles sometimes clustered – for example, antibiotic treatment, osmotic stress, and prolonged heat-stress were all associated with a common down-regulation of clusters related to capsule biosynthesis (C042, C029), electron transport (C113), and small molecular metabolism (C034) (Figure S6). Conversely, apparently similar perturbations sometimes yielded distinctive transcriptional profiles. For example, exposure of Bp to high salt (2M NaCl) or high sorbitol (2M) yielded distinct condition profiles, despite both conditions likely resulting in high osmotic stress. Bp may thus respond differently to salt- and non-ionic induced osmotic stress, similar to findings in *Synechocystis sp.* [26].

### Bp Clusters Facilitate Regulatory Motif Discovery

**Figure 5:**
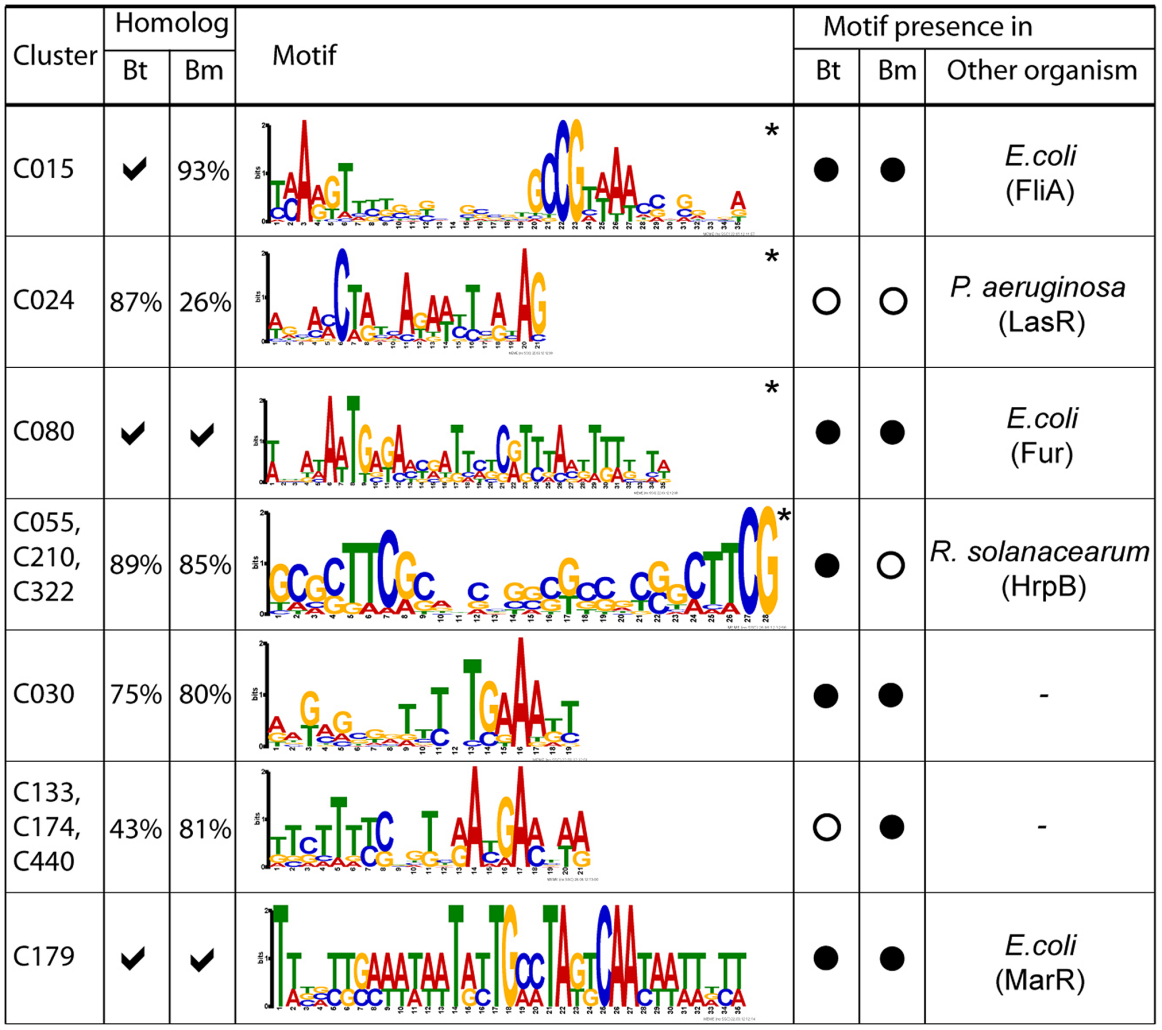
Discovery of *cis*-regulatory motifs. Motifs were identified by analysing upstream sequences of constituent genes or operons in each cluster. The asterisk (*) indicates that the motif was detected using MEME and BioProspector. Tick symbols indicate that all cluster genes have a cognate homolog in the specified species (i.e. 100%), otherwise the proportion of homologs in that species is reported. Filled circles indicate that the discovered *cis*-motifs in Bp are significantly similar (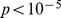) to Bt or Bm. Motifs that match to known binding sites and corresponding binding proteins in other species are reported in the last column. Bt, *B. thailandensis*; Bm, *B. mallei*.

To identify *cis*-regulatory motifs driving these condition-dependent expression programs, we used motif discovery algorithms to analyze upstream regions of co-expressed cluster genes [27], [28]. 194 clusters (41%) were commonly classified as over-represented in motifs (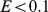) (Table S11). Supporting the accuracy of our results, we identified many motifs previously shown in other bacterial species to regulate similar programs. These included motifs matching the consensus binding sequences of *E. coli* FliA, for a Bp cluster associated with chemotaxis and mobility (C015) [29]; the Fur binding sequence for a Bp cluster related to cation biology (C080) [30]; the *P. aeruginosa* LasR binding sequence for a cluster related to secondary metabolism (C024); and the *R. solanacearum* HrpB binding sequence for clusters related to T3SS2 (C055, C210, C322) [31] (Figure 5).

Our analysis also identified previously unknown regulatory motifs. For example, we discovered a candidate *cis*-regulatory motif in C030 (*BPSS1512 – BPSS1533*), which is associated with T3SS3, a known mammalian virulence factor. Comparisons of homologs of *BPSS1512* – *BPSS1533* in Bm and Bt revealed that this motif is conserved in all three species, suggesting that it is functionally important. Other *cis*-regulatory motifs significantly conserved in Bt or Bm were found in clusters related to capsular biosynthesis (C133, C174, C440) (motif similarity in Bm, 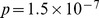, assessed by TOMTOM [32]), and antibiotic resistance (C179) (Bt, 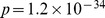; Bm, 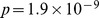). Regulatory motifs were not confined to genes, but were also associated with ncRNAs. Of 147 ncRNAs positively correlated to gene clusters with motifs, approximately 40% of the ncRNAs exhibited a similar motif in their upstream regions. For example, the ncRNAs *BPNC20041F* and *BPNC20065R* both exhibited upstream motifs similar to C080 and M016 (C055, C210, C322) respectively, which are regulated by Fur [30] and HrpB [31] (Figure 5, Table S12). Taken collectively, these results demonstrate the utility of the Bp condition compendium as a resource for regulatory motif discovery.

### Deconvolution of High-Complexity Transcriptome Profiles Using Condition-Dependent Clusters

We reasoned that the condition-dependent clusters, being associated to a diversity of *in vitro* experimental conditions, could be exploited as “molecular fingerprints” to deconvolute independent and high-complexity Bp transcriptomes of biological interest. As a proof-of-concept, preliminary analysis of two independent T3SS3 mutants (BsaN, and BprC) revealed that genes differentially expressed in T3SS3 mutants, were mapped onto the condition-dependent network, were associated with i) significantly closer network distances to the mutated gene compared to randomized gene sets (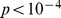), and ii) consistent down-regulation of condition-dependent clusters involved in Type III secretion (C030, C035) (Figure S7A,B, Text S1). To apply this concept to a more complex genetic scenario, we then used the Bp condition-dependent network to deconvolute the program of quorum sensing (QS), a genetic program in bacteria where changes in gene expression and cellular behaviour are linked to population density [33].

**Figure 6:**
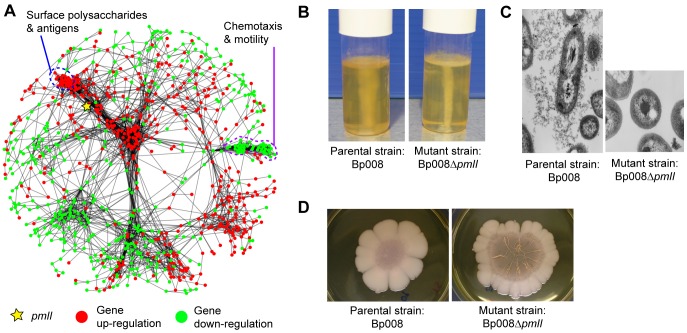
Condition-specific deconvolution of QS mutants. (A) *pmlI* transcriptional network. The diagram shows genes differentially expressed in *pmlI*-disrupted mutants (>2-fold change), overlaid onto the condition-dependent network. Red and green spots represent up- and down-regulated genes. Yellow star - location of the *pmlI* gene. Genes coding for chemotaxis/mobility (violet-dotted line) and surface polysaccharide antigens (blue-dotted line) are shown. (B) Motility assays. The wild type parental strain Bp008 is motile, as shown by the more turbid medium. The QS mutant is non-motile and only grows along the line of inoculation. (C) Electron microscope photographs of the Bp capsule. The exopolysaccharide material typical of Bp capsule I (CPSI) is apparent in the parental strain Bp008 as shown by the black streaks surrounding the rod-shaped bacterium. In contrast, neither exopolysaccharide material nor capsule architecture is observable in the mutant. (D) Disruption of QS system results in altered bacterial phenotype. The wild type parental strain Bp008 exhibits a smooth colony phenotype when grown on agar plate whereas the QS mutant has a wrinkled phenotype.

In Bp, genetic disruption of the PmlI-PmlR QS system has been shown to attenuate virulence in mouse infection models [34]. However, as hundreds of genes are regulated by QS systems, the specific molecular determinants underlying this virulence attenuation remain unclear. To deconvolute the PmlI/R profile, we first defined a “QS signature” of 1,187 genes (562 up-regulated, 625 down-regulated, Table S13), comprising genes significantly differentially expressed between quorum sensing mutants and wild-type Bp. The signature contained several genes previously reported to be associated with quorum sensing in Bp, including genes related to stationary phase growth (*BPSL1505* (*rpoS*)), other quorum sensing pathways (*BPSS1180* (*bpsI2*), *BPSS1176* (*bpsR2*)) and oxidative stress (*BPSL2863* (*dpsA*)) [34]–[37] (Table S14). Genes in the quorum sensing signature were then mapped onto the condition-dependent network. Of the 1,187 genes, 1,002 genes were successfully mapped onto the network. The QS signature genes were highly connected to one another (Figure 6A), exhibiting a level of modularity significantly higher than a randomized network (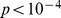, comparing weighted clustering coefficients; Figure S7C, Text S1). QS mutants exhibited down-regulation of condition-dependent clusters C015 and C021 (Figure 6A, violet dotted line), functionally related to chemotaxis (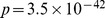) and flagella assembly (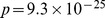), and up-regulation of cluster C029 related to Type III capsule biosynthesis (CPSIII; 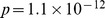). Notably, previous reports have demonstrated that flagella expression is required for full virulence in Bp [38]. CPSIII is non-essential for virulence [39], but its expression is reciprocal to the expression of genes involved in Type I capsule polysaccharides (CPSI), a major virulence determinant [25]. Indeed, we observed down-regulation of the CPSI biosynthesis gene *wzt2*, which encodes a component of the ABC transporter required for the delivery of capsular polysaccharides to the outer membrane [40].

We sought to validate these results at the phenotypic level. In motility assays, consistent with the network results we observed significant differences in mobility between wild type and mutant strain when cultured on soft agar, with the mutant being less motile (Figure 6B). To investigate if CPSI polysaccharides were effectively delivered to the outer membrane, we performed electron microscopy [41]. Unlike wild-type strains, CPSI polysaccharides were not effectively secreted in the QS mutant (Figure 6C), and when cultured on agar plates, the QS mutant exhibited a distinctively wrinkled colony morphology distinct from the smooth phenotypes of wild type strain (Figure 6D). These findings suggest that the altered virulence observed in Bp QS mutant is likely due to disruptions in two key virulence traits: flagella and CPSI activity.

Finally, we applied the condition-dependent network to deconvolute a Bp transcriptome profile associated with murine lung infection. Genes differentially regulated in Bp isolated from infected mouse lungs were significantly enriched in 9 condition-dependent clusters (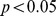, hypergeometric test). One upregulated cluster C030, comprised T3SS3 genes, likely reflecting a strong functional requirement for T3SS3 activity during lung colonization [42]. Among the upregulated genes, we identified five that might function as potential effector proteins - *BPSS1498* (*tssD-5*), *BPSL3319* (*fliC*), *BPSS1525* (*bopE*), *BPSS1529* (*bipD*) and *BPSS1532* (*bipB*). These effectors were identified using the program PSORTb 3.0 [43] – a subcellular localization prediction tool. Notably, several of these genes have been previously validated as secreted effector proteins [44], [45], and are thus likely to be secreted into lung cells to hijack host cellular pathways. Other clusters upregulated during lung infection (C080, C446, C087) contained genes involved in ferric ion acquisition, including *BPSL1775* (C446), an iron uptake receptor, and the pyochelin genes (*pch*) and *fptA* in cluster C087. The murine lung infection profile was also significantly similar to *in vitro* profiles related to nutrient starvation (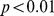, Text S1). The results indicate that two of the most strongly regulated pathways during Bp lung infection are T3SS3 and iron-acquisition (see Discussion).

## Discussion

In this study, we integrated strand-specific whole-genome transcriptional data over 80 environmental, chemical and genetic perturbations to generate a transcriptional condition compendium of Bp. Previous molecular studies on Bp have largely focused on protein-coding genes defined by the original genome annotation study [8]. However, our data suggests that additional functional elements are also likely to reside in the Bp genome. For example, of ∼500 putative novel genes identified by an alternative gene prediction algorithm (FGENESB [15]), 59% of these novel genes were associated with expressed transcripts indicating that they are transcribed. Notably, previous analysis of these novel genes has also shown that 46% are associated with other proteins in the COG, KEGG, STRING and NR databases, and high-confidence ribosome binding sites have also been identified in 60% of these novel genes [13]. Moreover, while several of these novel genes have short lengths (<500 bp), recent proteomic studies have confirmed the *bona-fide* expression of many short-length Bp genes [46], and in one study a newly identified short-length *Burkholderia* gene of 74 amino acids was experimentally demonstrated to regulate contact dependent growth inhibition [47]. Expression of short-length genes has also been confirmed in other bacterial species, such as MgtR in *Salmonella* (30 amino acids) [48], Sda in *Bacillus subtilis* (46 a.a.) [49], [50], YccB, YncL, YohP and IlvX in *Escherichia coli* (<50 a.a.) [51].

Besides potential coding genes, we also identified in this work >700 Bp condition-dependent ncRNAs. This is a conservative estimate, since in our study potential ncRNAs shorter than 100 bp were excluded from analysis due to challenges in resolving *bona-fide* ncRNA signals from background noise. ncRNAs are emerging as a major new class of regulatory molecules governing many aspects of prokaryote biology, including protein synthesis (e.g. tRNAs), cellular regulation (riboswitches) and cellular catalysis (ribozymes). ncRNAs associated with virulence and host-pathogen interactions have also been found in *Yersinia* spp and *E. coli* [52], [53]. In Bp, we identified several ncRNAs expressed under conditions plausibly linked to mammalian infection, such as *BPNC10134F*, *BPNC20132R*, *BPNC10175R* expressed in normal human serum and *BPNC10090R*, *BPNC20136F*, *BPNC20142R* expressed upon insulin exposure. Besides ncRNAs, we also discovered genome-wide expression of antisense RNAs in Bp. In other prokaryotes, antisense RNAs have been shown to modulate gene transcription by promoting RNA degradation or transcriptional interference [54], and in pathogens such as *H. pylori* and *L. monocytogenes*, antisense RNAs are involved in regulating metabolic enzymes and virulence factors [5], [55]. Taken collectively, these results strongly suggest that several features of Bp biology are likely to be modulated by other molecular entities beyond protein-coding genes, specifically ncRNAs and antisense RNAs.

Our data demonstrates that the two Bp chromosomes exhibit very different transcriptional landscapes. Specifically, Chr 1 genes were often constitutively and highly expressed, while Chr 2 genes exhibited “mosaic” expression, where distinct subsets of Chr 2 genes were expressed in a strongly condition-dependent manner. Previous genome analysis has also suggested that the two Bp chromosomes are distinct in composition and function, and Chr 1 has been proposed as a “housekeeping” chromosome. Interestingly, when compared against other prokaryotic transcriptome studies, the transcriptional landscape of Bp Chr 1 bears high resemblance to other single chromosomal microbes *E. coli*, *L. monocytogenes* and *B. subtilis* [4], [5], [56], while the consistently lower expression levels of Bp Chr 2 and its condition response profiles more closely resemble profiles previously observed in plasmid pXO_1_ in *B. anthracis* and pSymB in *S. meliloti*, respectively [57], [58]. Comparison of our gene expression data to previously published proteomic studies also revealed that there is a positive but modest correlation between transcript and protein data, as has been reported for other prokaryotes [46], [59]–[61] (Figure S8). However, to our best knowledge, this is the first formal report demonstrating the distinct transcriptional landscapes of multi-chromosomal bacteria, and suggests very different evolutionary origins for the two Bp chromosomes. Specifically, Bp Chr 1 is the ancestral chromosome with a transcriptional profile similar to single-chromosome pathogens, while Chr 2 is likely derived originally from an exogenous plasmid, which subsequently acquired sufficient numbers of essential genes to become an obligate part of the Bp genome. Interestingly, these findings may also explain the origins of other prokaryotes with multi-partite genomes (e.g. *Vibrio cholerae*).

Using the compendium data, we constructed a co-expression network of Bp genes. Co-expression networks are often useful for two major applications – functional discovery, and *cis*-regulatory motifs. For functional discovery, genes encoding proteins participating in the same pathway, or forming part of the same protein complex, often display patterns of co-regulation when surveyed across a large number of diverse conditions [23]. In the Bp network, examples of co-expressed genes included clusters related to motility, aerobic respiration, detoxification, and ribosomal function (Figure 4A). Besides known genes, such “guilt-by-association” approaches can also often shed light on genes with poorly-understood or unknown functions. Despite ongoing genome annotation efforts, many hypothetical and putatively assigned genes still exist in the BpK96243 genome, and less than 50% of Bp genes are currently annotated in the KEGG (Kyoto Encyclopedia of Genes and Genomes) PATHWAY database (www.genome.jp/kegg/pathway.html). Linking these genes to other co-expressed genes of known function may thus prove useful in inferring potential functions. For example, we highlighted a set of “hypothetical” protein-coding genes (*BPSL2828, BPSL2829*) which strongly co-expressed with genes associated with heat-shock and protein unfolding. Once identified, these genes can then be further tested through targeted experimentation. Indeed, ongoing *in silico* analyses by the PATRIC team have revealed that BPSL2829 is a heatshock protein GrpE. Besides protein-coding genes, we also discovered numerous associations between ncRNAs and the co-expressed genes. For example, the ncRNAs *BPNC20122R* and *BPNC20135F* were positively correlated with the T3SS3-related expression clusters C030 and C035 (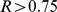, 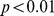), suggesting that these two ncRNAs might also influence Type III secretion activity.

For *cis*-regulatory motifs, we analyzed the Bp network to discover >190 candidate *cis*-regulatory motifs previously undescribed in Bp, related to biologically important functions such as iron uptake, motility and secondary metabolism. Several of these motifs were conserved in other distantly-related species, such as *E. coli* and *P. aeruginosa*, arguing that upstream regulatory pathways controlling these functions are likely to be conserved. In general, most of the newly detected motifs in our study remain uncharacterized. Possible explanations include (i) similar motifs in other species have not been studied, (ii) regulation of the same cellular process in Bp has been changed due to evolutionary pressures or (iii) the DNA-binding protein and the motif it recognizes have mutated in a parallel manner [62].

Finally, our study presents a general approach to integrate condition-dependent transcriptome data with genetic data, for the purpose of dissecting transcriptional profiles of biological interest but formidable complexity. Applying this concept to the process of quorum sensing, we were able to highlight two processes, cell motility and capsule formation, as likely contributors to the attenuation of virulence previously observed in a mutant genetically disrupted in *PmlI*, a master regulator of quorum sensing. We also used this approach to highlight T3SS3 and iron acquisition as two of the most highly regulated pathways during murine *in vivo* infection. A recent Bp study showed that the disruption of ferric-pyochelins and other iron acquisition mechanisms significantly reduced bacterial loads in murine lungs, though a *mba pch hmu hem* quadruple mutant was still capable of iron acquisition and inducing lethality in an acute murine melioidosis model [63]. In pyochelin-negative *B. cepacia* strains, exogenously supplied pyochelin increased bacterial virulence [64]. Collectively, these results imply that pyochelin-mediated iron acquisition may represent the preferred pathway amongst the numerous iron acquisition mechanisms encoded in the Bp genome for efficient iron uptake during host infection. The presence of many other iron acquisition genes and perhaps even novel ferritin-iron acquisition pathways could likely act as backup mechanisms in case Pch is ineffective, as observed in the quadruple mutant experiment [63].

In conclusion, similar to recent tiling microarray studies of other bacterial species [4], [5], [56], the Bp condition compendium presented here represents an important contribution to the melioidosis field, in its validation of previously described genes discovery and characterization of a host of novel genomic features, including ncRNAs, antisense transcripts, and co-expression clusters containing both known and hypothetical genes. Detailed experimental interrogation will be necessary to characterize the functional relevance of these genomic features to Bp regulation, physiology and pathogenicity.

## Materials and Methods

### Bacterial Strains and Conditions

Bp strains used are listed in Table S15. Strains were exposed to 82 separate conditions broadly classified under 21 major categories (Table S1). Manipulations of live bacteria were performed in a BioSafety Level 3 facility in DSO National Laboratories. For all conditions, a minimum of 2 biological replicates were used.

### BpK96243 Tiling Microarrays and Expression Profiling

High-density tiling arrays were fabricated by Roche NimbleGen (Roche NimbleGen, USA) based on the BpK96243 reference genome [8]. Bacterial RNAs were extracted and processed for microarray hybridization as described in [13]. In total, 166 samples were profiled; however one sample (K9BALBcLungs 1) had overwhelmingly high background and was excluded. The final Bp condition-specific compendium comprises 165 array profiles. Microarray images were analyzed by Roche NimbleScan software (Roche NimbleGen, USA), and LOWESS normalized (Locally Weighted Scatter Plot Smoother) by GeneSpring GX software (Agilent, USA). All arrays were median-normalized. Normalized signals from biological replicates were averaged to obtain a single, normalized, probe signal for each condition. Microarray data has been deposited into the Gene Expression Omnibus (GEO) under accession number GSE43205.

### Identification and Annotation of Transcriptionally Active Regions (TARs)

A moving window binomial approach was performed for *de novo* TAR identification [14] (Text S1). TARs were visualized using Artemis (Sanger, UK) or SignalMap (Roche NimbleGen, USA), and annotated against Sanger coding genes [8], ncRNAs (Rfam, [18]), FGENESB predicted genes [13], [15], and predicted operons. Genes passing a 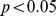 (Binomial test) cut-off were classified as expressed. Polycistronic operons were classified as expressed only if all gene members within the operon were classified as expressed in the same condition. Antisense transcripts were defined as expressed TARs mapping to the complementary strand of a Sanger or FGENESB gene, either spanning the entire gene or partially. Differential expression between conditions was determined by comparing the log-transformed median probe expression levels of probes corresponding to genic units (e.g. Sanger genes). Expression levels were visualized using GeneSpring GX 11.0 software (Agilent, USA), using a >2-fold change cutoff (Text S1).

### Identification of ncRNAs

We applied the following criteria to identify new candidate ncRNAs: i) the ncRNAs should be a subset of the identified TARs, ii) ncRNAs should be distinct from other genic features (e.g. protein-coding genes) by a minimum of 3 consecutive probes (105 bp), iii) ncRNAs are strictly located in intergenic regions, iv) ncRNAs should not be antisense to any genic feature, v) expression levels of probes corresponding to the ncRNA must be the top 90^th^ percentile and above of expressed probes, and vi) the minimum length of an ncRNA is 100 bp. Secondary structure predictions were performed using RNAfold [65].

### Gene Co-expression Networks and Co-expression Clusters

Co-expression associations between genes were defined by the ARACNe algorithm [21]. Each gene pair was assigned to a mutual information score (MIS) greater than zero, and we retained the top 2% of gene pairs (MIS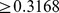). The MISs were also used to form a weighted adjacency matrix, and indirect interactions between gene pairs were identified and removed by ARACNe using a Data Processing Inequality strategy. The final network covers 5,387 genes connected by 60,024 direct interactions. Distances between adjacent genes were computed by subtracting the power transformed weight by its maximum, forming a distance matrix. The iGraph R package was used to compute the shortest distance between any two genes based on the distance matrix.

To define co-expression clusters, we identified groups of highly co-expressed genes using Markov Clustering (MCL) [22]. To identify the optimal level of cluster granularity, the clustering analysis was performed using different inflation parameters (1.0 - 3.5) and at each value the clustering results were evaluated for structural efficiency and functional coherence, measured by the fraction of gene pairs within the cluster sharing identical or similar Riley functional categories. We also confirmed the robustness of the cluster compositions by a leave-one-out validation approach where the network construction and clustering was repeated on a reduced data set with one sample removed in an iterative fashion. An observed cluster was deemed robust if at least 75% of the cluster composition was also observed in at least 95% of the reduced data sets. Stable clusters were compared to Riley's classifications, and functional annotations were assigned to clusters exhibiting a statistical over-representation of the same or similar annotations (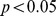, after Benjamini & Hochberg multiple testing correction). We also constructed maximum related cluster networks (MRCN), composed of highly weighted edges connecting different co-expression clusters [24]. To compute associations between any cluster pair 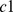 and 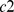, we quantified the number of highly connected links (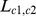) bridging 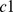 and 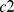
and calculated the Z-score of 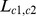:
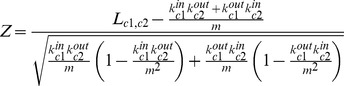
where 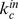 is the number of highly weighted links of cluster *c* incoming from other clusters or genes; 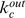 is the number of links outgoing to other clusters or genes; and *m* is the total number of highly weighted links bridging different clusters. Clusters connected by 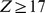
were deemed significant. The condition-dependent co-expression network was visualized using Cytoscape 2.8.1.

### Motif Identification

Candidate regulatory motifs were identified using the MEME algorithm [27], applied to sequence regions upstream of genes or the first operon gene (translational start site to 500 bp upstream). The background was set to a first order Markov model. Other MEME parameters were: (i) zero or one occurrence per gene, (ii) minimum width of 8 bp, and (iii) maximum width of 35 bp, (iv) motifs were not searched for on the reverse complement strand. Motifs were deemed to be significant if 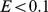. See Text S1 for the parameters used in BioProspector. Similarities between motifs in different *Burkholderia* species were measured using TOMTOM [32]. We also compared the discovered motifs against the Prodoric 8.9 database [66].

### Mouse Infection Assays

For mouse infections, female BALB/c mice (6–8 week-old; Harlan Laboratories, Bicester, Oxon, UK) were maintained under Animal Biohazard Containment Level 3 conditions. All animal experiments were performed in accordance with the guidelines of the Animals (Scientific Procedures) Act of 1986 and were approved by the local ethical review committee at the London School of Hygiene and Tropical Medicine. Prior to intranasal (i.n.) infection, mice were anesthetized intraperitoneally with ketamine (50 mg/kg; Ketaset; Fort Dodge Animal, Iowa, USA) and xylazine (10 mg/kg; Rompur; Bayer, Leverkusen, Germany) diluted in PFS. Challenge was performed by administering a total volume of 50 µl i.n. containing 2500 colony forming units (CFU) BpK96243. At day 3 post-infection (p.i.), mice were killed and lungs aseptically removed into 3 ml of cold Trizol Reagent (Invitrogen, CA, USA). Organs were homogenized using a Polytron homogenizer and samples stored at −80°C until further processing.

## Supporting Information

Figure S1 BpK96243 tiling microarray design and quantification. (A) Probes were tiled across both forward (+) and reverse (−) strands of the 2 Bp chromosomes. Red bars show the locations of annotated Sanger genes in the genome on the respective strands of the chromosomes. Blue bars represent the probes. (B) Schematic representative example of probes on the tiling array. Precise 50 mer reverse complement probes were designed for both top (forward) and bottom (reverse) strands of both chromosomes of BpK96243. Sense probes are located within predicted genes and on the coding chromosomal strand. Reverse complements of sense probes constitute the antisense probes. (C) Genes with high probe redundancy (probe counts). Two representative examples are shown. *BPSS1434* and *BPSS1439* are membrane anchored cell surface proteins found on Chr 2 with probe counts of 2 to 10. Each shares significant similarity with streptococal hemagglutinin. There are 16 adhesin genes encoding for proteins with conserved domains associated with the Hep-Hag family hemagglutinin-like proteins in the BpK96243 genome, out of which, 9 of them correspond to high probe redundancy in our array design. (D) Quantitation of array reproducibility and robustness. (i) Technical replicates. Scatter plot comparing signal intensities of all probes from 2 technical replicates of LBS were plotted and the Pearson coefficient of determination computed and shown at the bottom right. The dynamic range of signal intensities is limited by the scanner. (ii) Biological replicates. Scatter plot comparing the *probe expression ratios* of all probes from 2 biological replicates of LBS. The corresponding Pearson correlation coefficient is shown at the bottom right. For ii), note that expression ratios are being compared rather than absolute intensities. Thus, two replicate profiles are deemed reproducible if their probe ratios cluster around 0. More than 98% of probes lie within the acceptable range (grey dots). Blue dots (∼2%) represent outliers that are random.
(TIF)

Figure S2 Experimental RT-PCR validation of detected transcripts and novel genomic features. (A) Sanger gene expression. SignalMap snapshots of expressed Sanger genes belonging to different functional classifications and their respective RT-PCR validations (100 bp molecular ladder): i. DNA replication genes – *BPSL0074* (*dnaN*) and *BPSL0075* (*dnaA*) from K9LBS; ii. Motility gene – *BPSL3319* (*fliC*) from K942C16hrs; iii. Virulence genes (T3SS3) –*BPSS1546* (*bsaN*), *BPSS1525* (*bopE*), from K9UV1hr; iv. Capsule gene – *BPSL2800* (*wcbH*) from K930NHS. (B) FGENESB novel genes. PCR products using primers from Left to Right, 1. BPSL0393.1-F&R, 2. BPSL0706.1-F&R, 3. BPSL1304.1-F&R, 4. BPSL2880.1-F&R, 5. BPSL2882.1-F&R, 6. BPSS0035.1-F&R, 7. BPSS0279.1-F&R, 8. BPSS0818.1-F&R, 9. BPSS1773.1-F&R, 10. BPSS1927.1-F&R and Lane M: 100 bp molecular ladder. (C) Operons. Bp operon BpOpr0007 (*BPSL0026 – BPSL0032*). Operon expression from condition K9TaurineES was validated by RT-PCR. Regions between the gene members were amplified by primers as shown by black arrows above. (D) Antisense transcription. Experimental validation of antisense transcription of Sanger genes using strand-specific real-time PCR. Fourteen Sanger genes were experimentally validated; 7 of them exhibited normal gene expression (sense expression, Forward Primer, left) and 7 of them with associated antisense transcripts on the microarray (antisense expression, Reverse Primer, right). The figure shows concordance of results for most of the genes (except *BPSL0502* and *BPSL2540*) using strand-specific real-time PCR. (E) Non-coding RNA (ncRNAs). (i) Experimental validation of five novel ncRNA transcripts using RT-PCR; (ii) Experimental validation of ncRNA *BPNC10061R* transcripts under different conditions.
(TIF)

Figure S3 Antisense transcription of Bp operons. During *in vivo* infection (K9BalbcLungs), two operons belonging to T3SS3 exhibited antisense transcription: *BpOpr1082* (*BPSS1529* and *BPSS1530*) and *BpOpr1083* (*BPSS1531 – BPSS1533*).
(TIFF)

Figure S4 Expression heat-map of 16 genomic islands. Gene expression of 16 genomic islands (GIs) in the BpK96243 genome were normalized to mean zero across non-genetic perturbations. The over- and underexpression of genes are indicated in red and green, respectively. Overexpression of GIs in specific perturbations are marked by yellow boxes and the involved conditions are indicated on the right.
(TIF)

Figure S5 Sequence, chromosomal synteny conservation and differential expression of ncRNAs. (A) Total number of ncRNAs in *B.pseudomallei* K96243 being conserved in *B.cenocepacia* AU1054, HI2424, *B.mallei* ATCC23344 and *B.thailandensis* E264. The number of conserved ncRNAs in one species is indicated in brackets. (B) Shared synteny statistics. Conserved ncRNAs are flanked by (i) two homologs with conserved genes' order; (ii) two homologs with reversed genes' order; (iii) one homolog, the gene's order is either conserved or reversed. We determined the order of genes/homologs by using K96243 as reference. (C–D) The distributions of up-regulated and down-regulated ncRNAs across the conditions are shown respectively.
(TIF)

Figure S6Common down-regulated genes in Bp during exposure to antibiotic treatment, osmotic stress and prolonged heat stress. 158 genes were commonly down-regulated by at least 2-fold in the presence of chloramphenicol (K9Chlamp), ceftazidime (K9Ceft), 2M of sorbitol (K9Sorb) or under 42°C for 16 hours (K942C16hrs). Genes that are significantly enriched (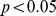, hypergeometric test) by clusters or Riley's functional annotations are indicated at the right column.(TIF)

Figure S7Visualization of condition-dependent networks of *bprC*, *bsaN* and weighted clustering coefficient properties of the *pmlI* network. (A) *bprC* transcriptional network. The distances between *bprC* and other differentially expressed genes are significantly shorter (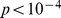). (B) *bsaN* transcriptional network. The distances between *bsaN* and other differentially expressed genes are significantly shorter (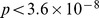). (C) The comparison between the observed weighted clustering coefficient (WCC) from the *pmlI* transcriptional network (blue line) and the distribution of WCC obtained from a set of randomized networks (red line).(TIF)

Figure S8Comparison between transcript levels from our study to protein levels from published literature. (A) The scatter plot shows the ratio of 28 transcripts to corresponding proteins in *rpoE* mutants compared to wild type [61]. Significance of the Pearson correlation coefficient (R) was tested using a two-tailed t-test. (B) Approximately 80% of Bp proteins detectibly expressed at early stationary phase [46] were also associated with detectible transcripts (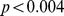, Text S1). These latter transcripts also exhibited higher expression signals compared to transcripts not associated with detectible proteins (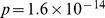, one-tailed Wilcoxon rank sum test). Taken together, transcript and protein abundance in Bp are positively but not perfectly correlated.(TIF)

Table S1 Details of conditions used for *Burkholderia pseudomallei* transcriptome.
(XLS)

Table S2 Overview of transcriptional landscape in *Burkholderia pseudomallei*.
(XLS)

Table S3 Primers for RT-PCR validation of transcripts and novel genomic features.
(DOC)

Table S4 Details of genes without detectible expression.
(XLS)

Table S5 The total number of expressed genomic features in each condition.
(XLS)

Table S6 Predicted secondary structures of novel Bp ncRNAs using RNAFold.
(DOC)

Table S7 Details of transcriptional landscape comparison between chromosome 1 (Chr1) and chromosome 2 (Chr2) in each condition.
(XLS)

Table S8 Coexpression network analysis revealed a collection of gene clusters, functional enrichments in the clusters and associations between clusters - maximum relatedness cluster network (MRCN).
(XLS)

Table S9 Correlation between ncRNAs and clusters.
(DOC)

Table S10 Condition subgroups identified by condition clustering.
(DOC)

Table S11 *cis*-regulatory motifs detected using genic upstream sequences from clusters and MRCNs.
(DOC)

Table S12 ncRNAs with identified *cis*-regulatory motifs.
(DOC)

Table S13 Differential expression of quorum sensing signature genes.
(DOC)

Table S14 Quorum-sensing (QS) associated genes found in literature.
(DOC)

Table S15 Bacterial strains and sources used in this study.
(DOC)

Text S1 Supplemental methods and supplemental references.
(DOC)
