# Supplementary figures and images for: The Condition-Dependent Transcriptional Landscape of Burkholderia pseudomallei
Source: PLoS Genet. 2013 Sep 12;9(9):e1003795. doi: 10.1371/journal.pgen.1003795 (PMC3772027; doi:10.1371/journal.pgen.1003795)

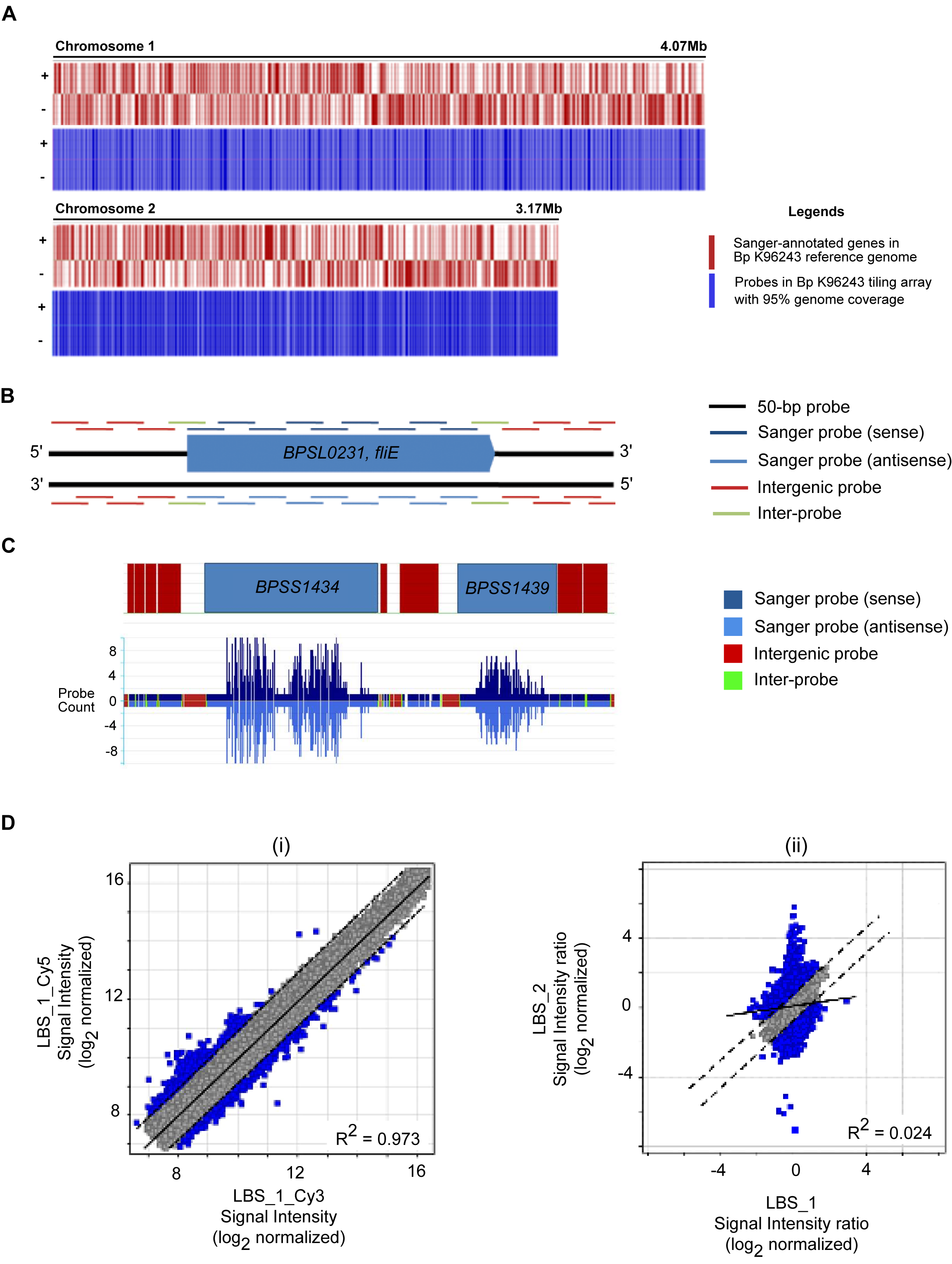

Supplement: Figure S1 — BpK96243 tiling microarray design and quantification. (A) Probes were tiled across both forward (+) and reverse (−) strands of the 2 Bp chromosomes. Red bars show the locations of annotated Sanger genes in the genome on the respective strands of the chromosomes. Blue bars represent the probes. (B) Schematic representative example of probes on the tiling array. Precise 50 mer reverse complement probes were designed for both top (forward) and bottom (reverse) strands of both chromosomes of BpK96243. Sense probes are located within predicted genes and on the coding chromosomal strand. Reverse complements of sense probes constitute the antisense probes. (C) Genes with high probe redundancy (probe counts). Two representative examples are shown. BPSS1434 and BPSS1439 are membrane anchored cell surface proteins found on Chr 2 with probe counts of 2 to 10. Each shares significant similarity with streptococal hemagglutinin. There are 16 adhesin genes encoding for proteins with conserved domains associated with the Hep-Hag family hemagglutinin-like proteins in the BpK96243 genome, out of which, 9 of them correspond to high probe redundancy in our array design. (D) Quantitation of array reproducibility and robustness. (i) Technical replicates. Scatter plot comparing signal intensities of all probes from 2 technical replicates of LBS were plotted and the Pearson coefficient of determination computed and shown at the bottom right. The dynamic range of signal intensities is limited by the scanner. (ii) Biological replicates. Scatter plot comparing the probe expression ratios of all probes from 2 biological replicates of LBS. The corresponding Pearson correlation coefficient is shown at the bottom right. For ii), note that expression ratios are being compared rather than absolute intensities. Thus, two replicate profiles are deemed reproducible if their probe ratios cluster around 0. More than 98% of probes lie within the acceptable range (grey dots). Blue dots (∼2%) represent out [file pgen.1003795.s001.tif]

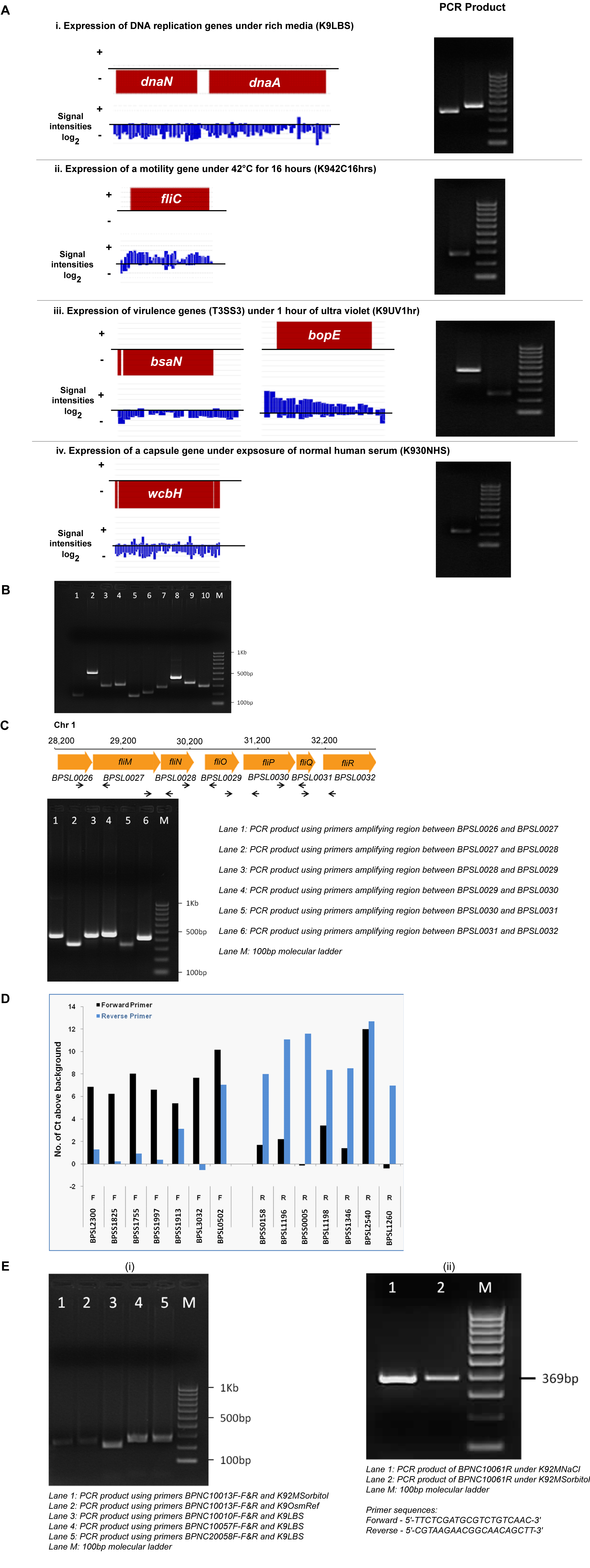

Supplement: Figure S2 — Experimental RT-PCR validation of detected transcripts and novel genomic features. (A) Sanger gene expression. SignalMap snapshots of expressed Sanger genes belonging to different functional classifications and their respective RT-PCR validations (100 bp molecular ladder): i. DNA replication genes – BPSL0074 (dnaN) and BPSL0075 (dnaA) from K9LBS; ii. Motility gene – BPSL3319 (fliC) from K942C16hrs; iii. Virulence genes (T3SS3) –BPSS1546 (bsaN), BPSS1525 (bopE), from K9UV1hr; iv. Capsule gene – BPSL2800 (wcbH) from K930NHS. (B) FGENESB novel genes. PCR products using primers from Left to Right, 1. BPSL0393.1-F&R, 2. BPSL0706.1-F&R, 3. BPSL1304.1-F&R, 4. BPSL2880.1-F&R, 5. BPSL2882.1-F&R, 6. BPSS0035.1-F&R, 7. BPSS0279.1-F&R, 8. BPSS0818.1-F&R, 9. BPSS1773.1-F&R, 10. BPSS1927.1-F&R and Lane M: 100 bp molecular ladder. (C) Operons. Bp operon BpOpr0007 (BPSL0026 – BPSL0032). Operon expression from condition K9TaurineES was validated by RT-PCR. Regions between the gene members were amplified by primers as shown by black arrows above. (D) Antisense transcription. Experimental validation of antisense transcription of Sanger genes using strand-specific real-time PCR. Fourteen Sanger genes were experimentally validated; 7 of them exhibited normal gene expression (sense expression, Forward Primer, left) and 7 of them with associated antisense transcripts on the microarray (antisense expression, Reverse Primer, right). The figure shows concordance of results for most of the genes (except BPSL0502 and BPSL2540) using strand-specific real-time PCR. (E) Non-coding RNA (ncRNAs). (i) Experimental validation of five novel ncRNA transcripts using RT-PCR; (ii) Experimental validation of ncRNA BPNC10061R transcripts under different conditions. (TIF) [file pgen.1003795.s002.tif]

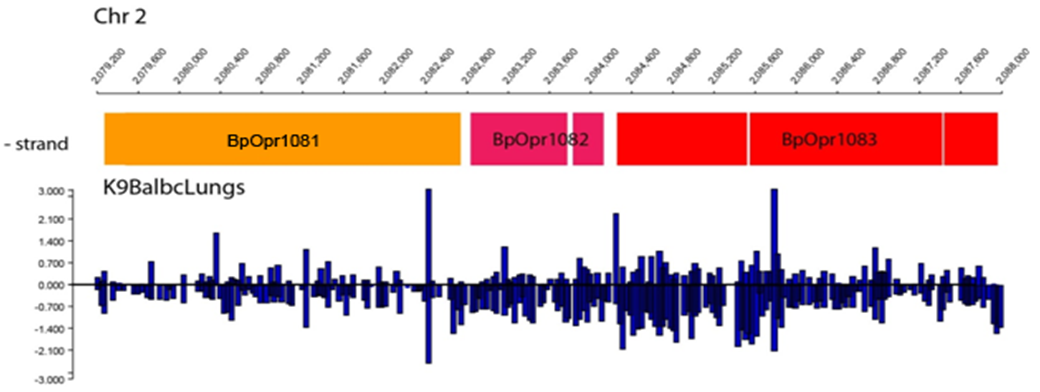

Supplement: Figure S3 — Antisense transcription of Bp operons. During in vivo infection (K9BalbcLungs), two operons belonging to T3SS3 exhibited antisense transcription: BpOpr1082 (BPSS1529 and BPSS1530) and BpOpr1083 (BPSS1531 – BPSS1533). (TIFF) [file pgen.1003795.s003.tif]

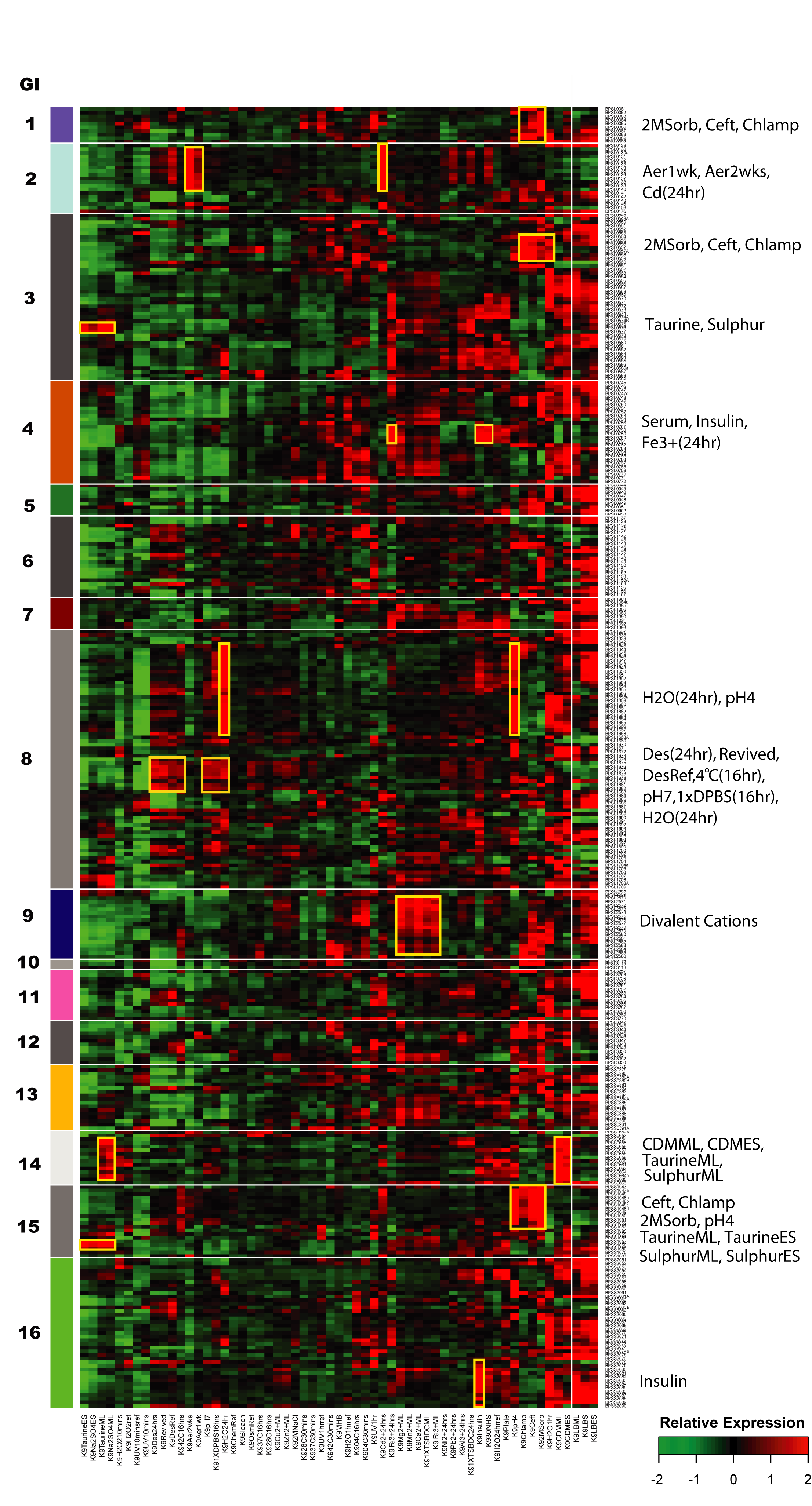

Supplement: Figure S4 — Expression heat-map of 16 genomic islands. Gene expression of 16 genomic islands (GIs) in the BpK96243 genome were normalized to mean zero across non-genetic perturbations. The over- and underexpression of genes are indicated in red and green, respectively. Overexpression of GIs in specific perturbations are marked by yellow boxes and the involved conditions are indicated on the right. (TIF) [file pgen.1003795.s004.tif]

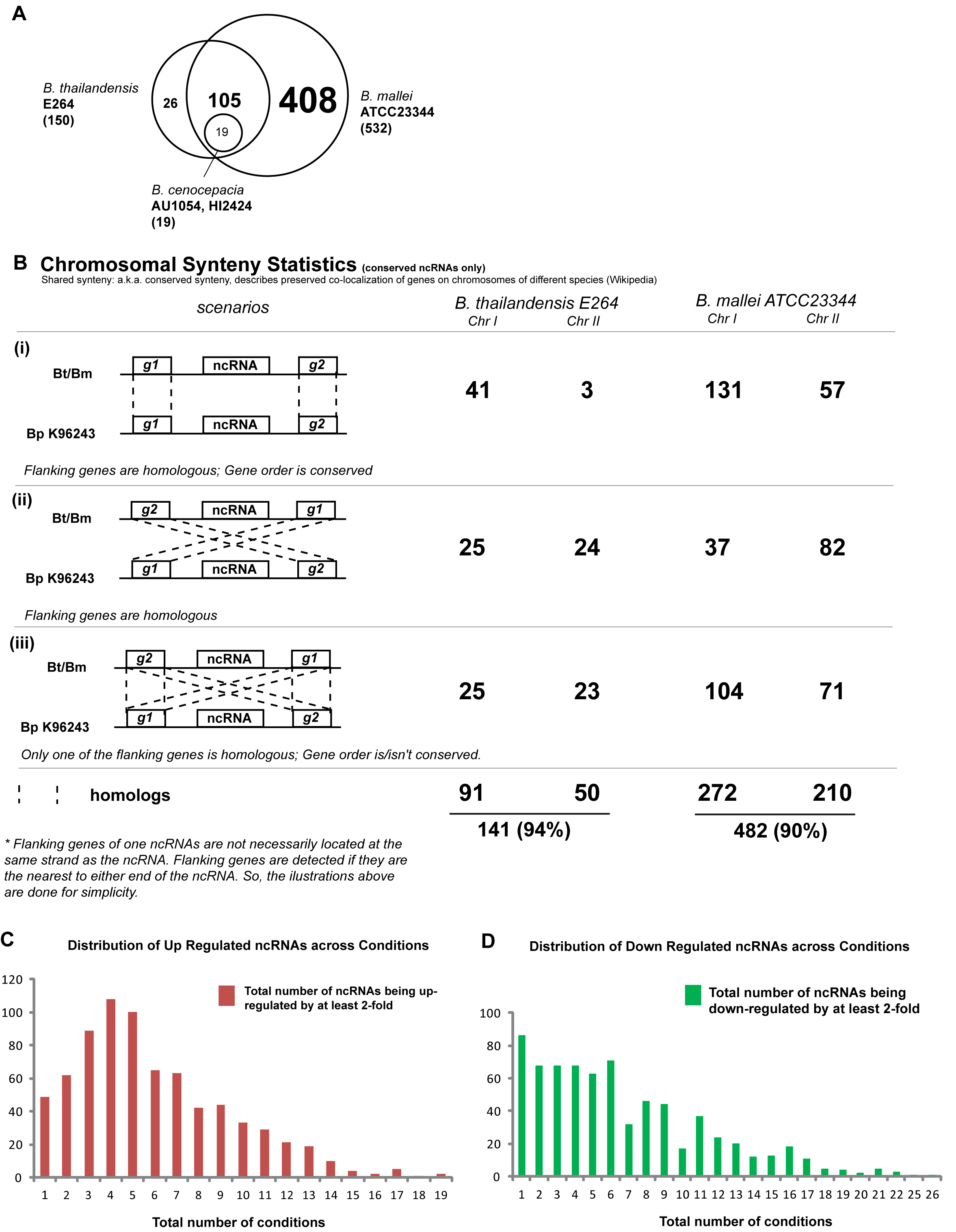

Supplement: Figure S5 — Sequence, chromosomal synteny conservation and differential expression of ncRNAs. (A) Total number of ncRNAs in B.pseudomallei K96243 being conserved in B.cenocepacia AU1054, HI2424, B.mallei ATCC23344 and B.thailandensis E264. The number of conserved ncRNAs in one species is indicated in brackets. (B) Shared synteny statistics. Conserved ncRNAs are flanked by (i) two homologs with conserved genes' order; (ii) two homologs with reversed genes' order; (iii) one homolog, the gene's order is either conserved or reversed. We determined the order of genes/homologs by using K96243 as reference. (C–D) The distributions of up-regulated and down-regulated ncRNAs across the conditions are shown respectively. (TIF) [file pgen.1003795.s005.tif]

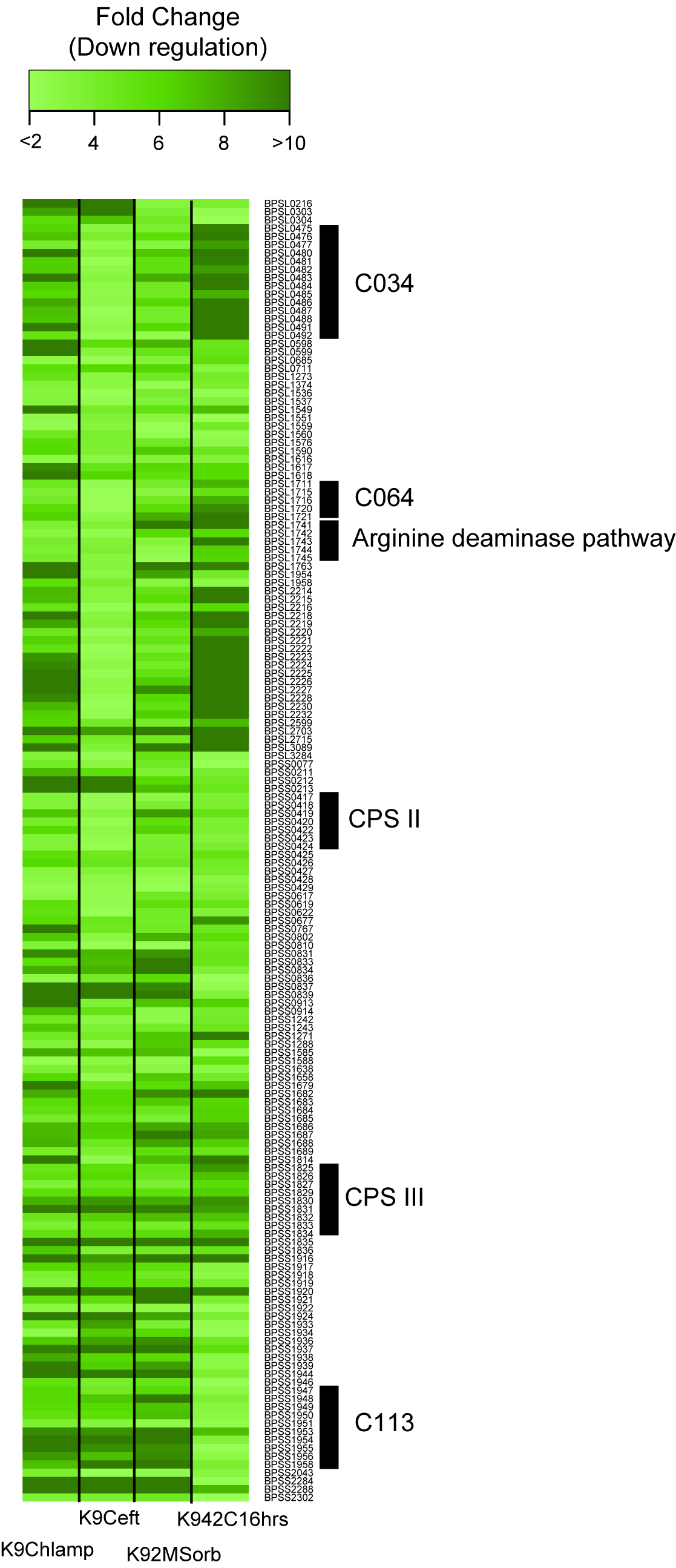

Supplement: Figure S6 — Common down-regulated genes in Bp during exposure to antibiotic treatment, osmotic stress and prolonged heat stress. 158 genes were commonly down-regulated by at least 2-fold in the presence of chloramphenicol (K9Chlamp), ceftazidime (K9Ceft), 2M of sorbitol (K9Sorb) or under 42°C for 16 hours (K942C16hrs). Genes that are significantly enriched (, hypergeometric test) by clusters or Riley's functional annotations are indicated at the right column. (TIF) [file pgen.1003795.s006.tif]

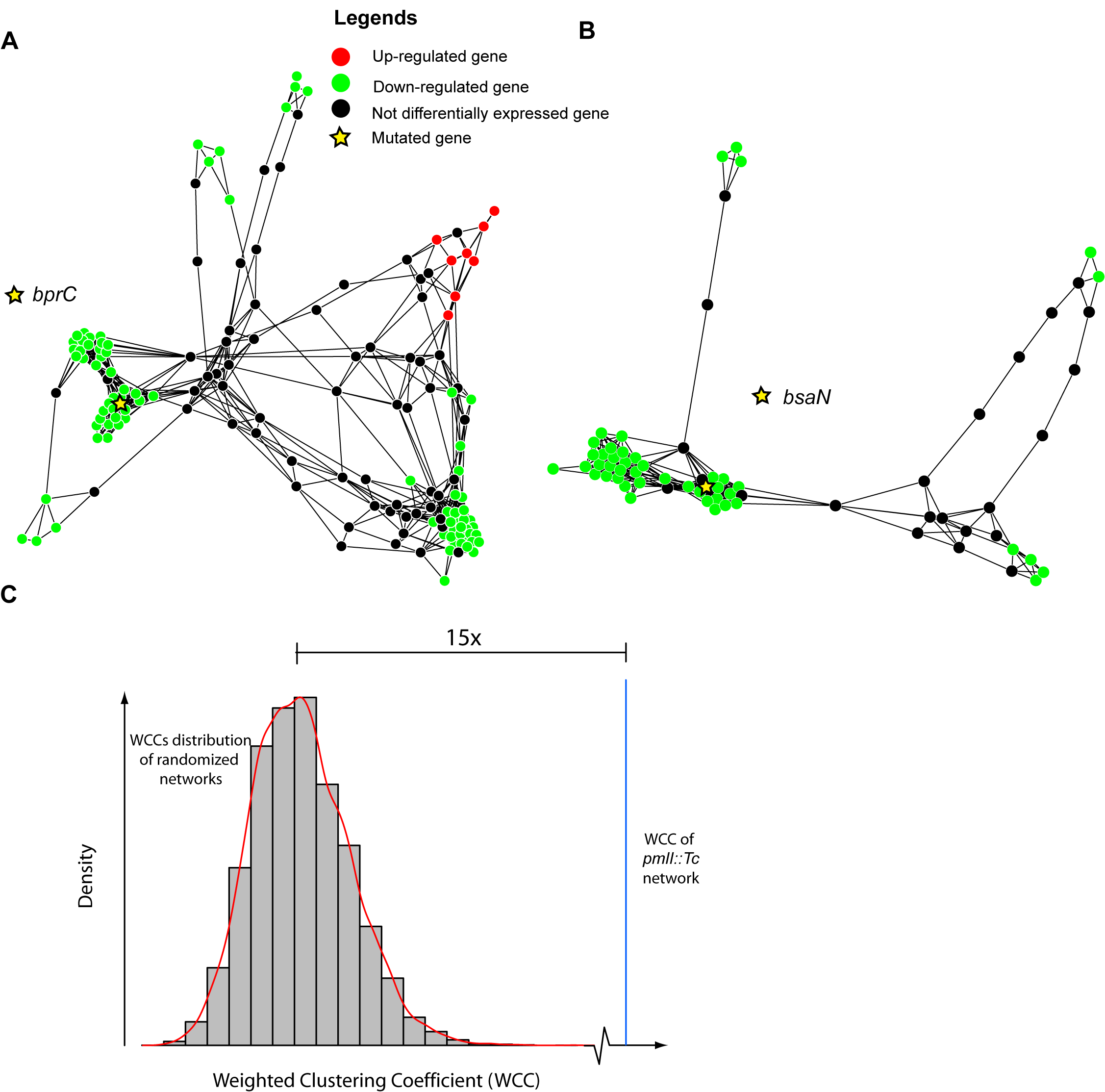

Supplement: Figure S7 — Visualization of condition-dependent networks of bprC, bsaN and weighted clustering coefficient properties of the pmlI network. (A) bprC transcriptional network. The distances between bprC and other differentially expressed genes are significantly shorter (). (B) bsaN transcriptional network. The distances between bsaN and other differentially expressed genes are significantly shorter (). (C) The comparison between the observed weighted clustering coefficient (WCC) from the pmlI transcriptional network (blue line) and the distribution of WCC obtained from a set of randomized networks (red line). (TIF) [file pgen.1003795.s007.tif]

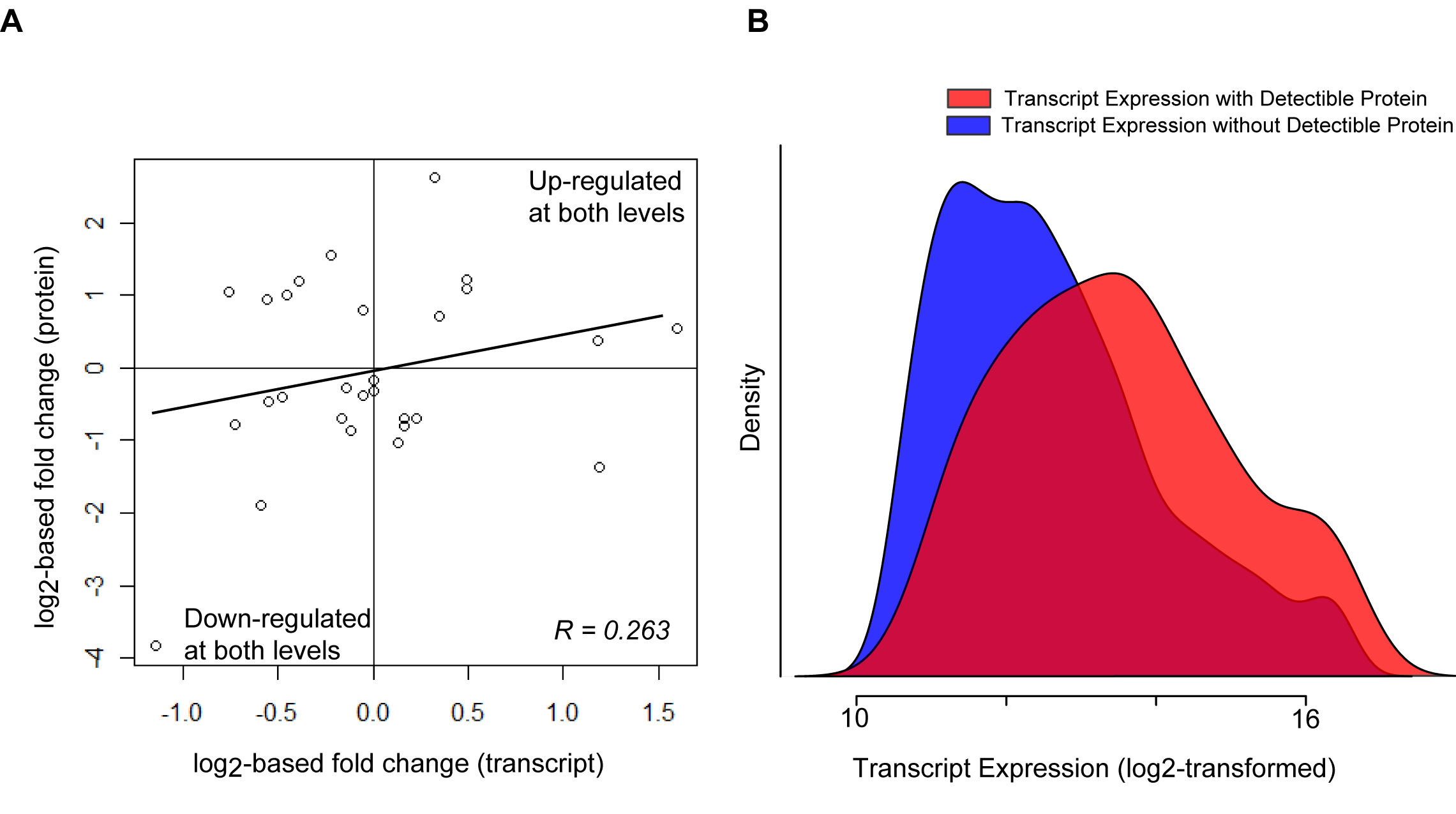

Supplement: Figure S8 — Comparison between transcript levels from our study to protein levels from published literature. (A) The scatter plot shows the ratio of 28 transcripts to corresponding proteins in rpoE mutants compared to wild type [61]. Significance of the Pearson correlation coefficient (R) was tested using a two-tailed t-test. (B) Approximately 80% of Bp proteins detectibly expressed at early stationary phase [46] were also associated with detectible transcripts (, Text S1). These latter transcripts also exhibited higher expression signals compared to transcripts not associated with detectible proteins (, one-tailed Wilcoxon rank sum test). Taken together, transcript and protein abundance in Bp are positively but not perfectly correlated. (TIF) [file pgen.1003795.s008.tif]
