# Supplementary material for: The Condition-Dependent Transcriptional Landscape of Burkholderia pseudomallei
Source: PLoS Genet. 2013 Sep 12;9(9):e1003795. doi: 10.1371/journal.pgen.1003795 (PMC3772027; doi:10.1371/journal.pgen.1003795)
Supplement: Table S3 — Primers for RT-PCR validation of transcripts and novel genomic features. (DOC) [file pgen.1003795.s011.doc]

Table S3. Primers for RT-PCR validation of transcripts and novel genomic features.

1. Sanger genes

| **Primer Name** | **SangerID/**  **Gene name** | | | **Sequence** | **Product size (bp)** | | | **Condition** |
| --- | --- | --- | --- | --- | --- | --- | --- | --- |
| DNA replication genes | | | | | | | | |
| BPSL0074-F  BPSL0074-R | *BPSL0074*/  *dnaN* | | ACTACCTGAACGGGATGCTG  TACGCCCTTGAACTTGTCG | | 386 | | K9LBS | |
| BPSL0075-F  BPSL0075-R | *BPSL0075*/  *dnaA* | | CGCACGCAGGAAGAGTTC  GGCGACCTTGATGTTGTAGA | | 444 | | K9LBS | |
| Motility genes | | | | | | | | |
| BPSL3319-F  BPSL3319-R | *BPSL3319*/  *fliC* | | ACAACGGCAAGAACATCCTC  TCTGATCGGTGAACGTGAAG | | 273 | | K942C16hrs | |
| BPSL0782-F  BPSL0782-R | *BPSL0782*/  *pilA* | | GATTCCCGCCTATCAGGATT  CACCAGCACGAGCGTATTC | | 244 | | K91XDPBS16hrs | |
| Capsule genes | | | | | | | | |
| BPSL2800-F  BPSL2800-R | *BPSL2800*/  *wcbH* | | TACAACCTGGGCAACAATGA  ACACCTCCCGCATAACTCAG | | 227 | | K930NHS | |
| BPSL2803-F  BPSL2803-R | *BPSL2803*/  *wcbE* | | GGTTGAGCCGAGAAAGAATC  CGACGGAGTAAGGGTTGAAG | | 365 | | K930NHS | |
| Virulence genes (T3SS3) | | | | | | | | |
| BPSS1525-F  BPSS1525-R | *BPSS1525*/  *bopE* | GTTCGCACAACAGATCAAGC  CGTCGCTTCGTAGACTTGC | | | | 259 | K9UV1hr | |
| BPSS1546-F  BPSS1546-R | *BPSS1546*/  *bsaN* | TTATCGGCATCGGTCAATTC  CGAGAAGTGGGACGAAGAC | | | | 527 | K9UV1hr | |
| Secondary metabolism genes | | | | | | | | |
| BPSS0581-F  BPSS0581-R | *BPSS0581*/  *pchA* | GCTTGCGAAACTGCTGGT  GCACGACCTTGCTGAACG | | | | 260 | K9BALBcLungs | |
| BPSS1006-F  BPSS1006-R | *BPSS1006* | ACGGCTTCTACGACGAGGAC  CCTTGCCCTGCTTCAGATT | | | | 306 | K9Al3+24hrs | |

1. FGENESB novel genes

| **Primer Name** | **SangerID/**  **Gene name** | **Sequence** | **Product size (bp)** | **Condition** |
| --- | --- | --- | --- | --- |
| BPSL0393.1-F  BPSL0393.1-R | *BPSL0393.1* | CGGGATGACGCACTGTCG  GGCCCGATGTGATGTATTC | 187 | K9LBS |
| BPSL0706.1-F  BPSL0706.1-R  BPSL1304B.1-F  BPSL1304B.1-R  BPSL2880.1-F  BPSL2880.1-R  BPSL2882.1-F  BPSL2882.1-R  BPSS0035.1-F  BPSS0035.1-R  BPSS0279.1-F  BPSS0279.1-R  BPSS0818.1-F  BPSS0818.1-R  BPSS1773.1-F  BPSS1773.1-R  BPSS1927.1-F  BPSS1927.1-R | *BPSL0706.1*  *BPSL1304B.1*  *BPSL2880.1*  *BPSL2882.1*  *BPSS0035.1*  *BPSS0279.1*  *BPSS0818.1*  *BPSS1773.1*  *BPSS1927.1* | CGAGAGCTGAGTGAGTGGAA  ACGCTGCCCGTATCGAAG  CGTGTTGAGGTCGAGCATT  TCCACTATCCGCTTTCTGCT  CTATTGCCCCGTTTGACATT  GGCAGGTTTCTGTCGAGATG  CGATCGACACCCGAGCAG  GGAGGGGCGACGACAGAC  AGCGCCGTTCGAGTGTGG  GAGCATCGTCACCGACATT  TCGACATCGGCGTGATTAT  GAGGGAAACGCTTGAGACAA  GCGACAAGAACGACGACAC  GCAGGCGATGGTAGAAGAAG  GTTTCCTCAAAAACCGATGC  AAAGCAAGCGATTCCAAGTC  GATTCCGTTTCGATTTCGTC  GCGTTCGTGTGTTGCATATC | 495  297  315  182  220  285  426  341  298 | K9Al3+24hrs  K9H2O1hr  K9Ceft  K9Ceft  K9Ceft  K9LBS  K9LBS  K9Al3+24hrs  K9LBS |

1. Operons

Primers for RT-PCR validation of Bp operon *BpOpr0007*. Primers were designed to amplify intergenic regions of the gene members of the operons.

| **Primer Name** | **Between**  **SangerID genes** | **Sequences** | **Product size (bp)** |
| --- | --- | --- | --- |
| *BPSL0026* | *BPSL0026* and *BPSL0027* | Forward- gtcttccggccctttcag  Reverse- ccttccttcgacagcatga | 365 |
| *BPSL0027* | *BPSL0027* and *BPSL0028* | Forward- gaatacctgacgcagcacat  Reverse- gaagttgaagatgccgatcc | 442 |
| *BPSL0028* | *BPSL0028* and *BPSL0029* | Forward- ggtgatggagtgcggatac  Reverse- atcaggtcgatgtcgttgtg | 349 |
| *BPSL0029* | *BPSL0029* and *BPSL0030* | Forward- gaagctgaaccgatgaaacc  Reverse- cgagcacgagccaggtat | 451 |
| *BPSL0030* | *BPSL0030* and *BPSL0031* | Forward- cgatacctggctcgtgct  Reverse- cgctcaacgaataggtcgtg | 468 |
| *BPSL0031* | *BPSL0031* and *BPSL0032* | Forward- cgatcttcatcccgtttctc  Reverse- gtcgcctcgttgatctgc | 341 |

1. Antisense transcription

List of primers used for strand-specific real-time PCR validation of antisense transcription.

| **Primer Name** | **SangerID/Gene name** | **Sequence** |
| --- | --- | --- |
| BPSL_2300_F_4  BPSL_2300_R_4  BPSS_1825_F_5  BPSS_1825_R_5  BPSS_1755_F_7  BPSS_1755_R_7  BPSS_1997_F_12  BPSS_1997_R_12  BPSS_1913_F_18  BPSS_1913_R_18  BPSL_3032_F_21  BPSL_3032_R_21  BPSL_0502_F_22a  BPSL_0502_R_22a  BPSS_0158_F_3  BPSS_0158_R_3  BPSL_1196_F_9  BPSL_1196_R_9  BPSS_0005_F_10  BPSS_0005_R_10  BPSL_1198_F_11  BPSL_1198_R_11  BPSS_1346_F_14  BPSS_1346_R_14  BPSL_2540_F_15  BPSL_2540_R_15  BPSL_1260_F_19  BPSL_1260_R_19 | *BPSL2300*  *phnB*  *BPSS1825*  *hepB*  *BPSS1755*  *BPSS1997*  *oxa*  *BPSS1913*  *lysP*  *BPSL3032*  *ftsL*  *BPSL0502*  *cydA*  *BPSS0158*  *BPSL1196*  *ilvI*  *BPSS0005*  *kbl*  *BPSL1198*  *ilvC*  *BPSS1346*  *BPSL2540*  *BPSL1260* | ATTGATGATCGGCGTGAAGT  GACAACCTCGTGTTCAAGCA  GTAGCGCGACGTGAGAATC  ACTTCGCGCTGTACACGTTC  GACGACGATGTGCAGGATCT  CGTTCGAGAAGGAAGGCTAC  AGCTTGCGATTGAGCATCTT  TCAAGTATTCGGTCGTGTGG  GTCAGGATCACCGCGTTC  GCATCCTGCTGTTCTACGTG  GCATCTTCAGCGAGCTTGTC  CTGATCATCGTGATGGGATG  GATCCGGAACGAGAAGAACA  ACGTTCGATCAGCACAAGC  TACAGCGGCATGAACGTCTA  AAATTGAAGTTGCCCGGATT  TGGCAGCAGATCGAATACAG  CGGTCTGGAAGTCGAGAAAC  AAAACGACGCGATCATCAG  ATGCCGTCCATCGAGAATAC  GGCATCATCGAGACGAACTT  ATCAGGTCGACGATCAGCTT  CACGATGCTCTGGCTTCC  GCGGCTTCAGGTAATCGTC  GTCGGCGTATTCATGATGG  AGAGCTTGAGCACGACGAAC  CACGCGATCGTCTACTTCTG  GAACACCGATTGCAGGAACT |

1. Non-coding RNAs (ncRNAs)

List of primers used for RT-PCR validation of Bp novel ncRNAs.

It must be mentioned that some of the RT-PCR validation of these ncRNAs were not successful (marked with *) and this was not unexpected as some ncRNAs are known to form looping structures and other secondary structures [1].

| **Primer Name** | **ncRNA** | **Sequence** |
| --- | --- | --- |
| BPNC10013F-F  BPNC10013F-R  BPNC20080F-F*  BPNC20080F-R*  BPNC10010F-F  BPNC10010F-R  BPNC10023F-F*  BPNC10023F-R*  BPNC10042F-F*  BPNC10042F-R*  BPNC10057F-F  BPNC10057F-R  BPNC20010F-F*  BPNC20010F-R*  BPNC20012F-F*  BPNC20012F-R*  BPNC20058F-F  BPNC20058F-R  BPNC20124F-F*  BPNC20124F-R* | *BPNC10014F*  *BPNC20080F**  *BPNC10010F*  *BPNC10023F**  *BPNC10042F**  *BPNC10057F*  *BPNC20010F**  *BPNC20012F**  *BPNC20058F*  *BPNC20124F** | ATGCTCCTTTCACCAAGTCG  CGCTCGAGATGAAAAATGC  GTCGAGCCCGCCGTAGAT  CAGCCGATGAGCGCGAAG  CGGGAAAGTGAGGTTTTCCT  CTCGGTCATCGCAAATGG  GATTCGCTGCGCTTCGTT  CCGGTCATCGCGGCGTTC  TGGCGACATAATGTTTTTATTTG  ACGCCGCGCCGCCGAAAT  CTGGCGCTCTTCCAGTGT  GCTGAACCTCTTCGCGTATC  GGTAGACGAGCGTGAAGAGC  ATTCGATGGGGAGTGAACTG  CTCGAGGCGCTGTTCTCG  GAATCGAGCCCGCGGATG  CAAGAACCTGCCGCTCTACT  AGTTCTCGGTCCAGATGTCG  CTCGTGCGACCTGTTCGT  CCGCCGACGTCGAAGCAC |

**REFERENCE**

1. Weinberg Z, Barrick JE, Yao Z, Roth A, Kim JN, et al. (2007) Identification of 22 candidate structured RNAs in bacteria using the CMfinder comparative genomics pipeline. Nucleic Acids Res 35: 4809-4819.
