# Supplementary material for: The Condition-Dependent Transcriptional Landscape of Burkholderia pseudomallei
Source: PLoS Genet. 2013 Sep 12;9(9):e1003795. doi: 10.1371/journal.pgen.1003795 (PMC3772027; doi:10.1371/journal.pgen.1003795)
Supplement: Table S6 — Predicted secondary structures of novel Bp ncRNAs using RNAFold. (DOC) [file pgen.1003795.s014.doc]

Table S6. Predicted secondary structures of novel Bp ncRNAs using RNAFold. The strand information is embedded in the last character of each ncRNA’s identifier (F: forward strand; R: reverse strand). Start and stop coordinates indicated in red refer to truncated regions of the respective ncRNAs used for analytical purposes. The color of each nucleotide indicates the likelihood of base pairing, ranging from red (most likely) to purple (least likely).

| **No.** | **ID** | **Chromosome** | **Start** | **Stop** | **Secondary Structure** |
| --- | --- | --- | --- | --- | --- |
| 1 | BPNC10011F | Chr1 | 210605 | 210866 | 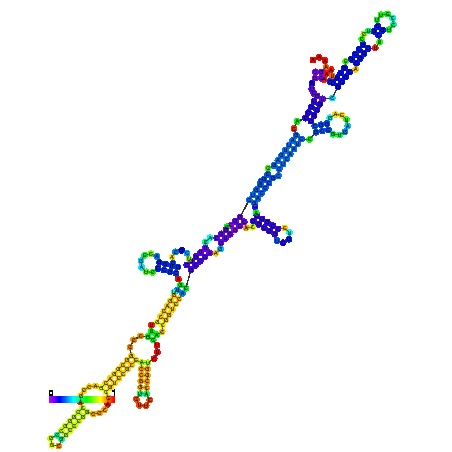 |
| 2 | BPNC10013F | Chr1 | 266668 | 266901 | 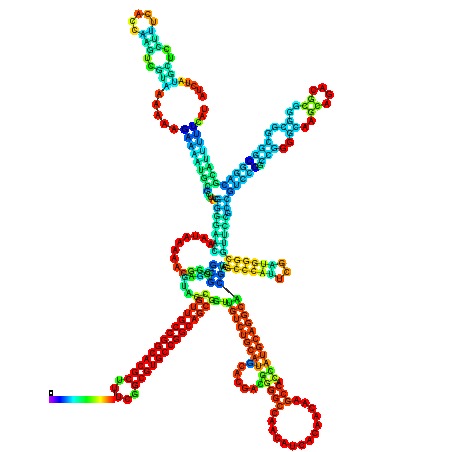 |
| 3 | BPNC10020F | Chr1 | 316896 | 317621 | 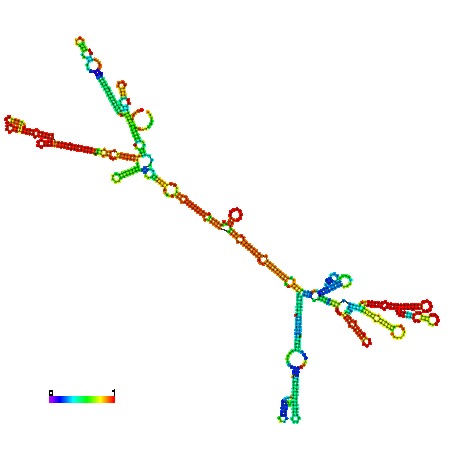 |
| 4 | BPNC10035F | Chr1 | 686949 | 687074 | 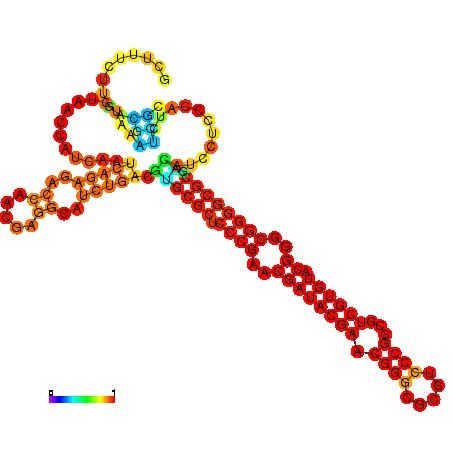 |
| 5 | BPNC10061R | Chr1 | 1042763 | 1043269 | 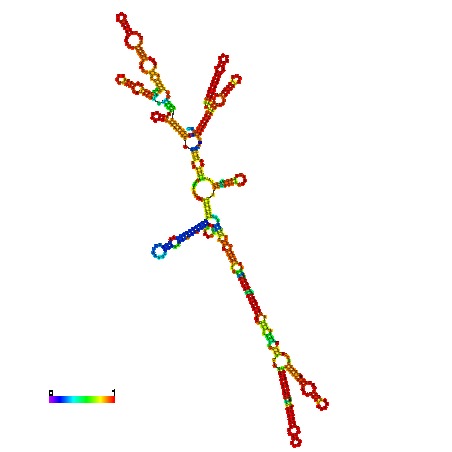 |
| 6 | BPNC10063R | Chr1 | 1090819 | 1091001 | 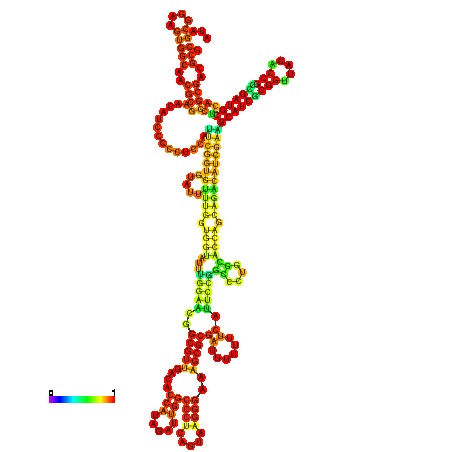 |
| 7 | BPNC10067R | Chr1 | 1162251 | 1162372 | 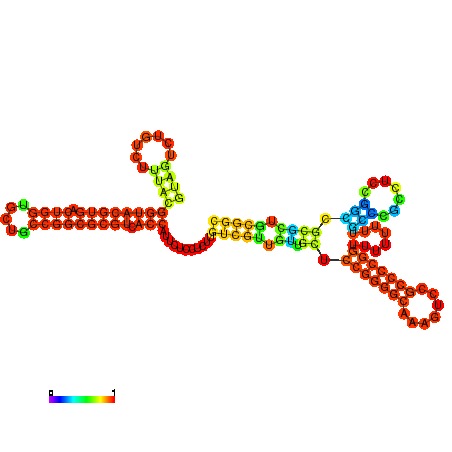 |
| 8 | BPNC10077R | Chr1 | 1225393 | 1225581 | 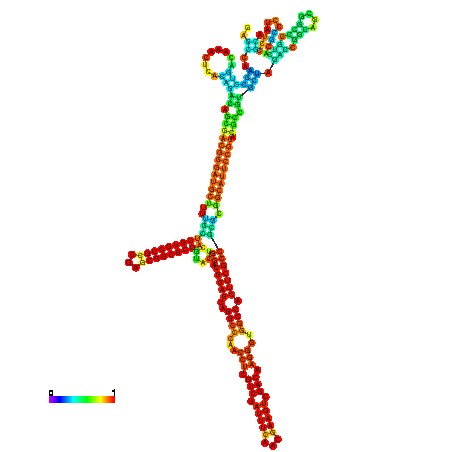 |
| 9 | BPNC10094F | Chr1 | 1575010 | 1575195 | 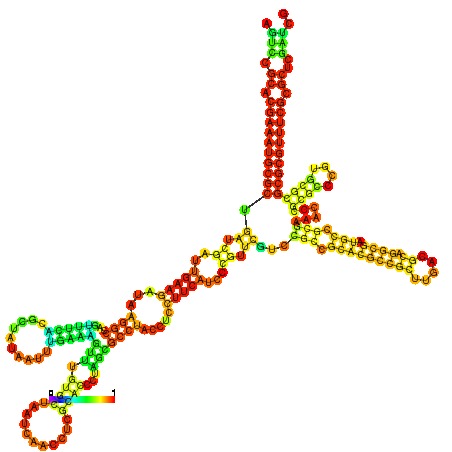 |
| 10 | BPNC10094R | Chr1 | 1565307 | 1565612 | 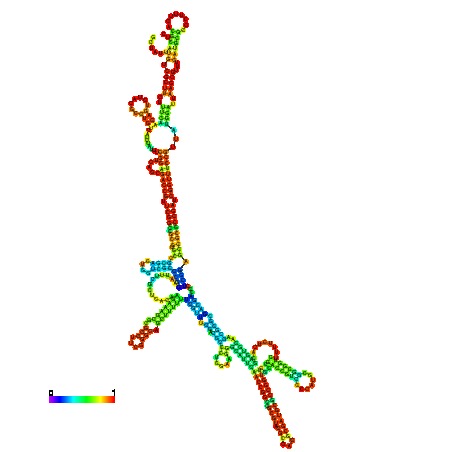 |
| 11 | BPNC10099F | Chr1 | 1636496 | 1636864 | 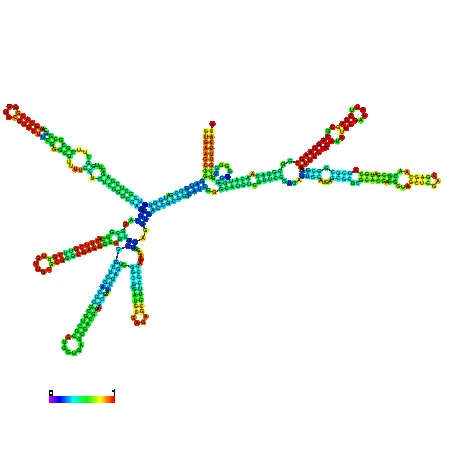 |
| 12 | BPNC10103R | Chr1 | 1642840 | 1642986 | 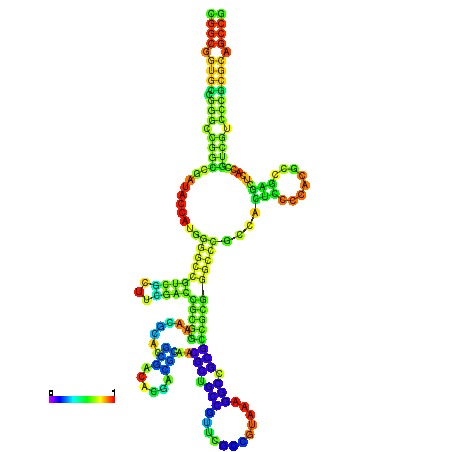 |
| 13 | BPNC10105F | Chr1 | 1802467 | 1802700 | 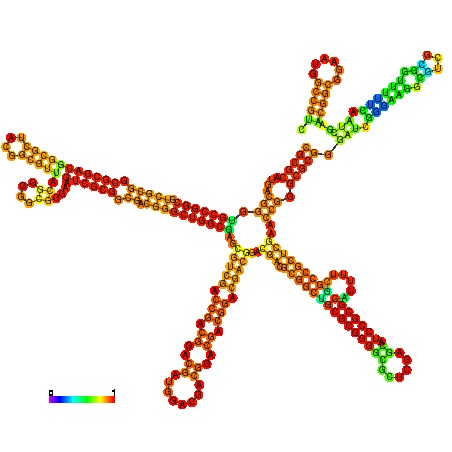 |
| 14 | BPNC10105R | Chr1 | 1702795 | 1703266 | 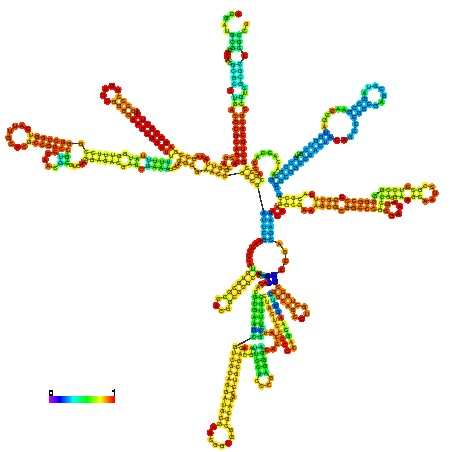 |
| 15 | BPNC10107R | Chr1 | 1802503 | 1802700 | 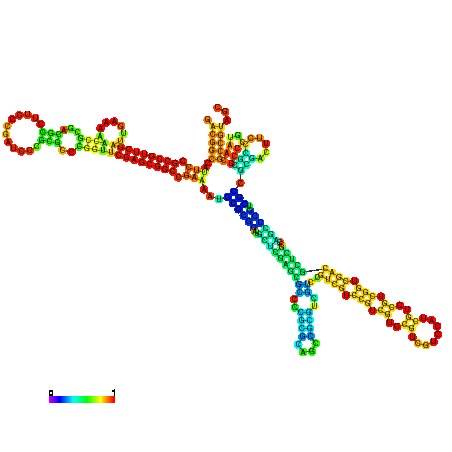 |
| 16 | BPNC10108F | Chr1 | 1834326 | 1834481 | 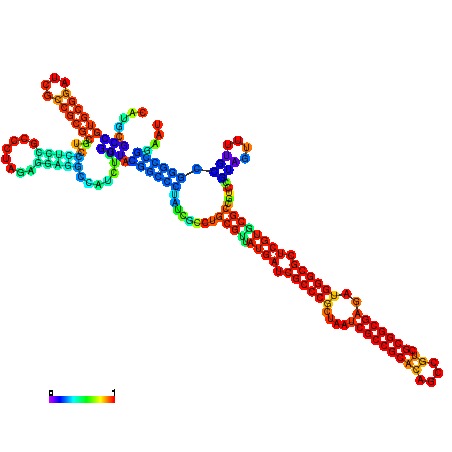 |
| 17 | BPNC10124F | Chr1 | 2196260 | 2196415 | 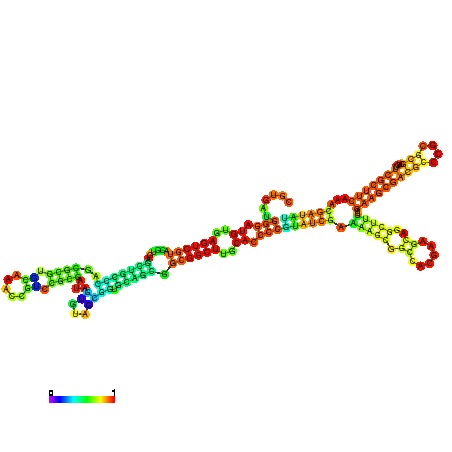 |
| 18 | BPNC10126R | Chr1 | 2290410 | 2290526 | 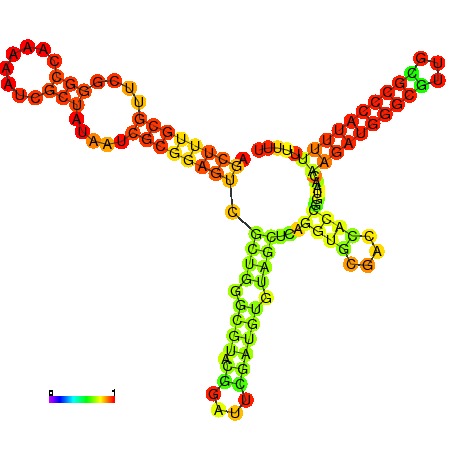 |
| 19 | BPNC10129R | Chr1 | 2330373 | 2330606 | 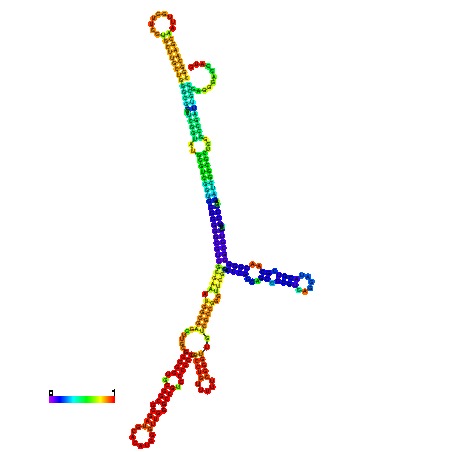 |
| 20 | BPNC10132R | Chr1 | 2352393 | 2352506 | 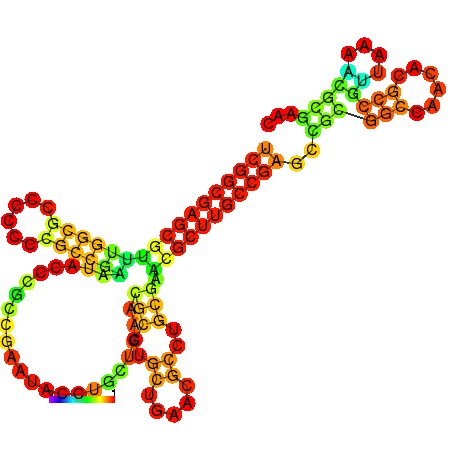 |
| 21 | BPNC10143F | Chr1 | 2551293 | 2551523 | 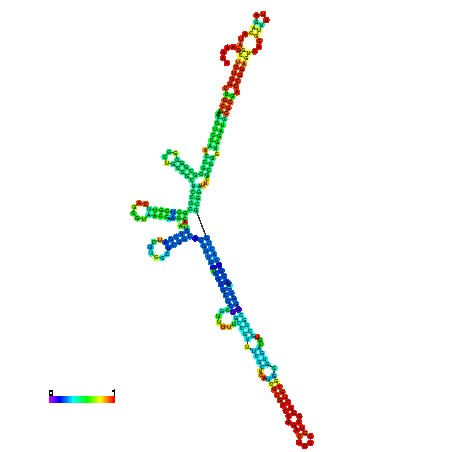 |
| 22 | BPNC10157R | Chr1 | 2751738 | 2751866 | 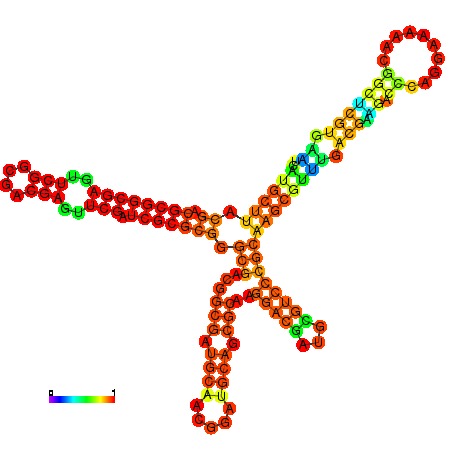 |
| 23 | BPNC10162R | Chr1 | 2803824 | 2804147 | 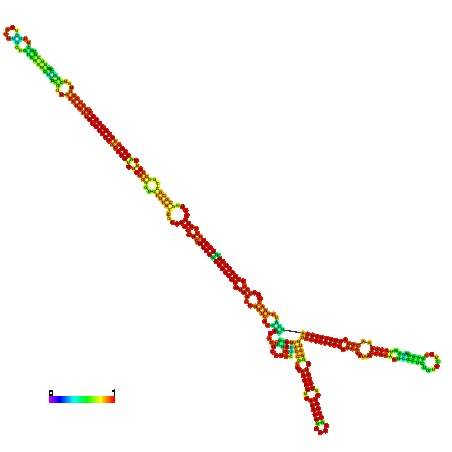 |
| 24 | BPNC10163F | Chr1 | 3054494 | 3054613 | 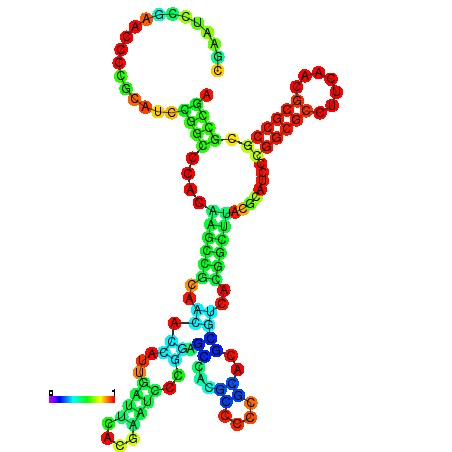 |
| 25 | BPNC10171F | Chr1 | 3245314 | 3245427 | 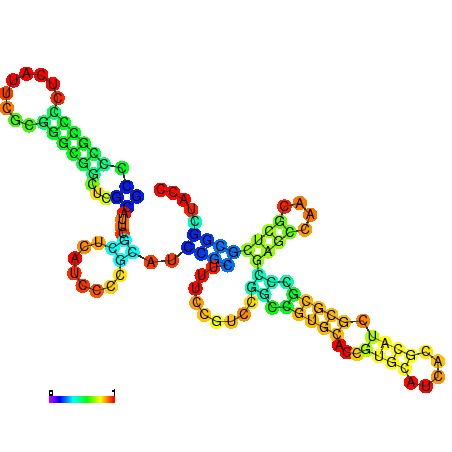 |
| 26 | BPNC10175R | Chr1 | 3071012 | 3071131 | 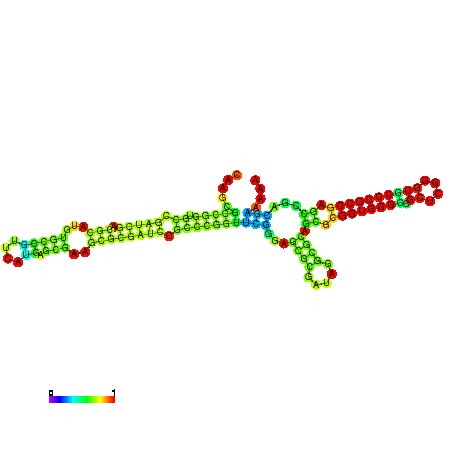 |
| 27 | BPNC10178F | Chr1 | 3399137 | 3399292 | 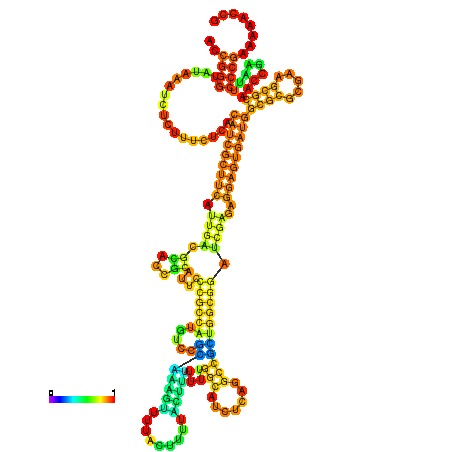 |
| 28 | BPNC10179R | Chr1 | 3154035 | 3154226 | 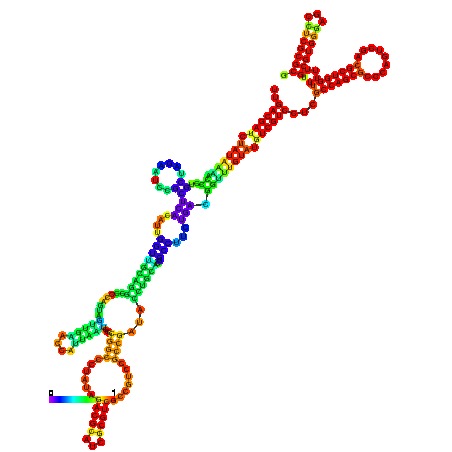 |
| 29 | BPNC10183R | Chr1 | 3225567 | 3225791 | 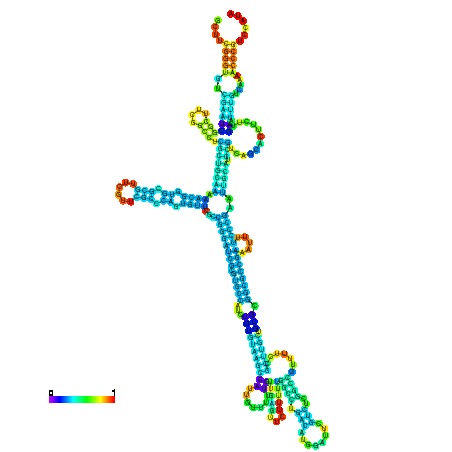 |
| 30 | BPNC10185F | Chr1 | 3639761 | 3639886 | 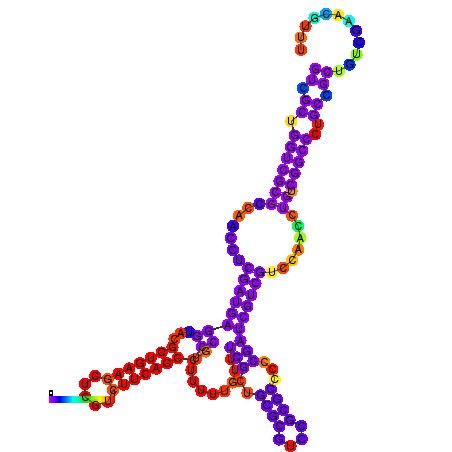 |
| 31 | BPNC10194R | Chr1 | 3427736 | 3427846 | 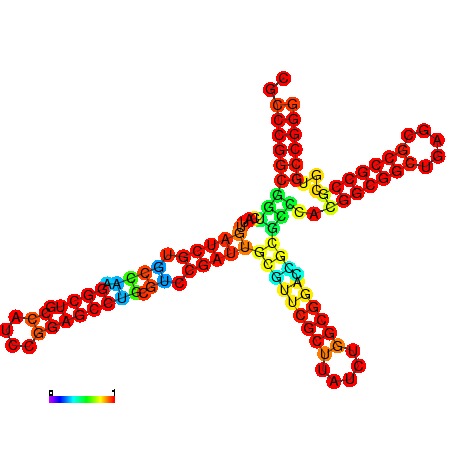 |
| 32 | BPNC10196F | Chr1 | 3824634 | 3824783 | 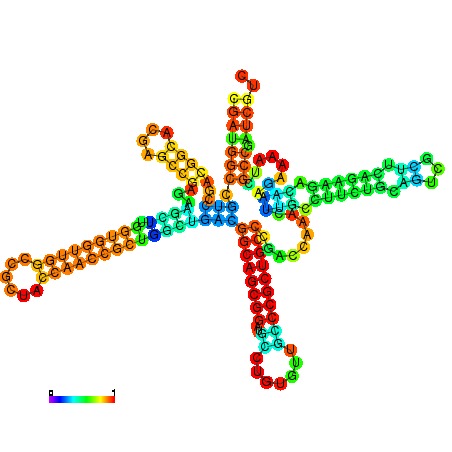 |
| 33 | BPNC10205F | Chr1 | 4031793 | 4031915 | 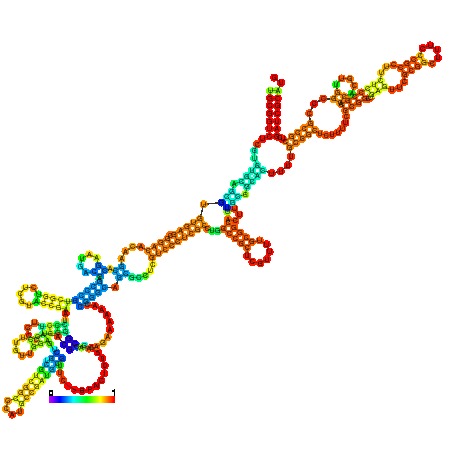 |
| 34 | BPNC10225R | Chr1 | 3937187 | 3937381 | 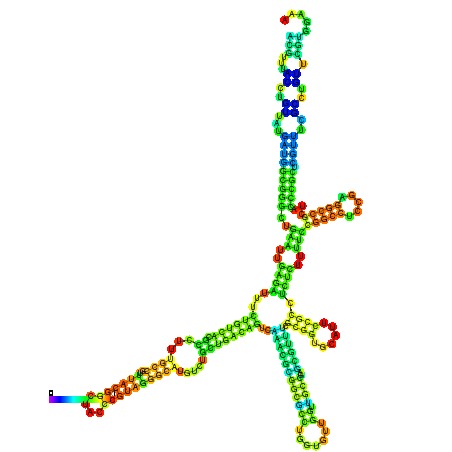 |
| 35 | BPNC10232R | Chr1 | 4054372 | 4054491 | 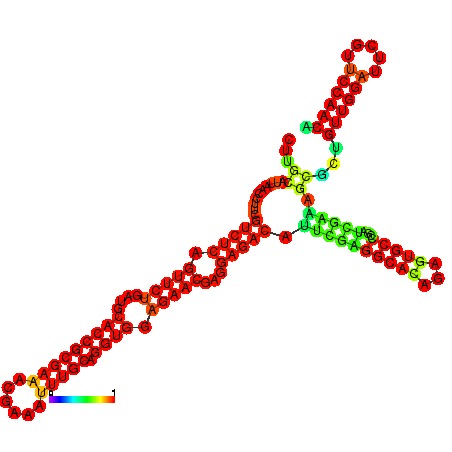 |
| 36 | BPNC10233R | Chr1 | 4059556 | 4059738 | 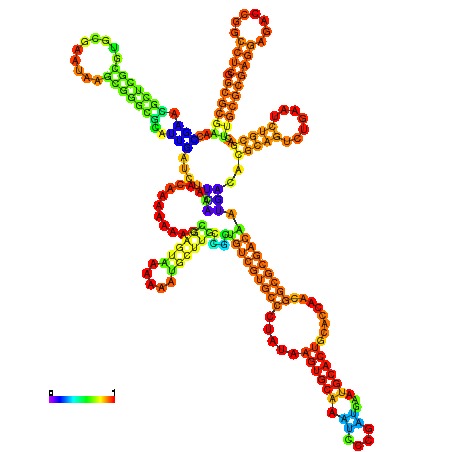 |
| 37 | BPNC10234R | Chr1 | 4074115 | 4074511 | 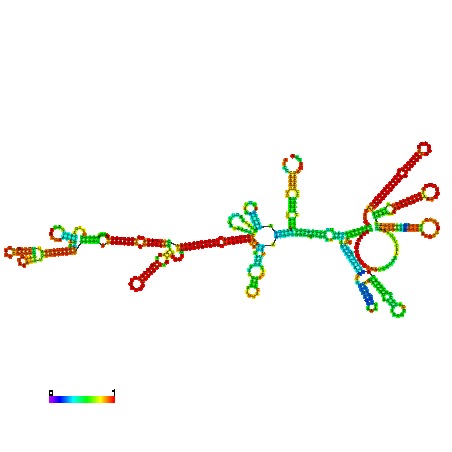 |
| 38 | BPNC20019F | Chr2 | 370416 | 370562 | 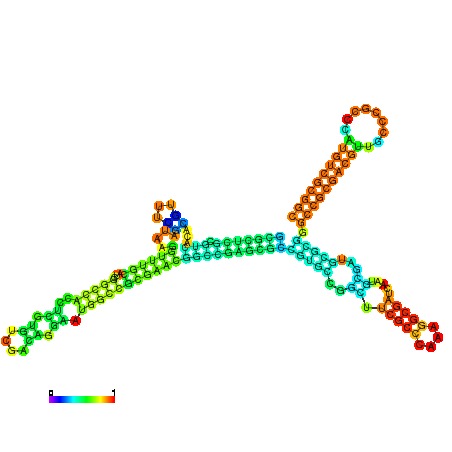 |
| 39 | BPNC20020R | Chr2 | 254105 | 254326 | 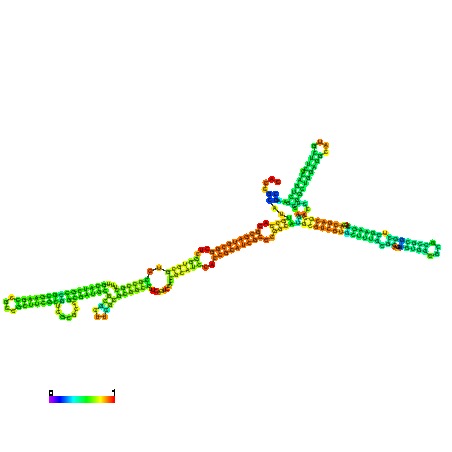 |
| 40 | BPNC20027F | Chr2 | 451816 | 451971 | 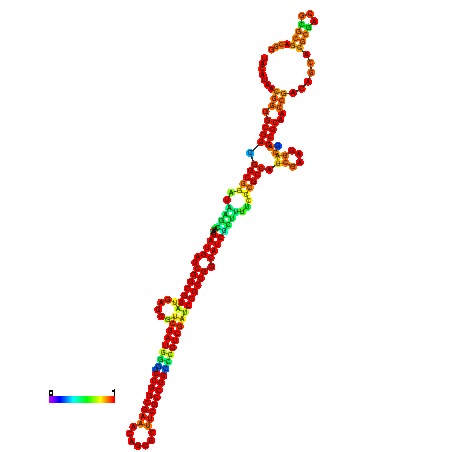 |
| 41 | BPNC20028R | Chr2 | 397045 | 397164 | 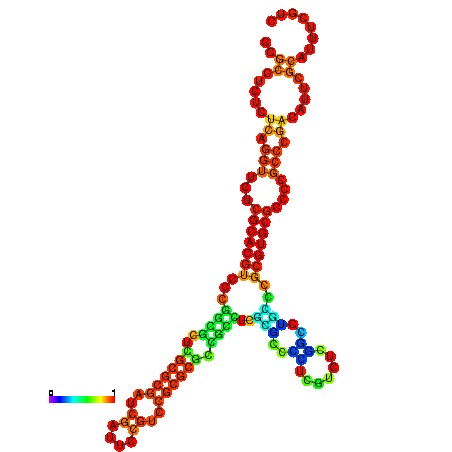 |
| 42 | BPNC20040R | Chr2 | 671442 | 671738 | 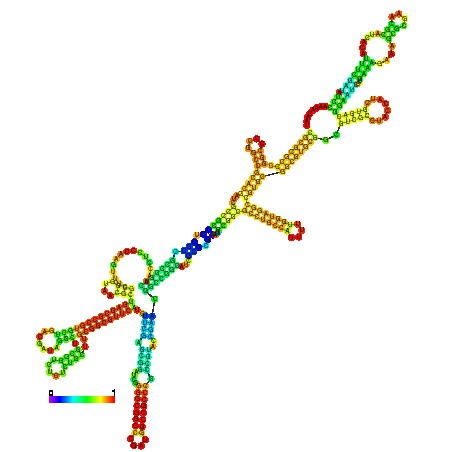 |
| 43 | BPNC20042R | Chr2 | 777600 | 777722 | 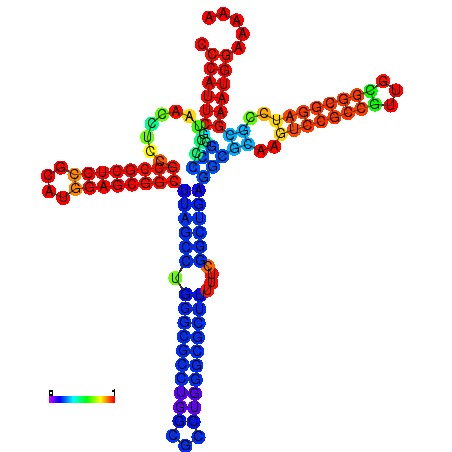 |
| 44 | BPNC20045F | Chr2 | 819989 | 820492 | 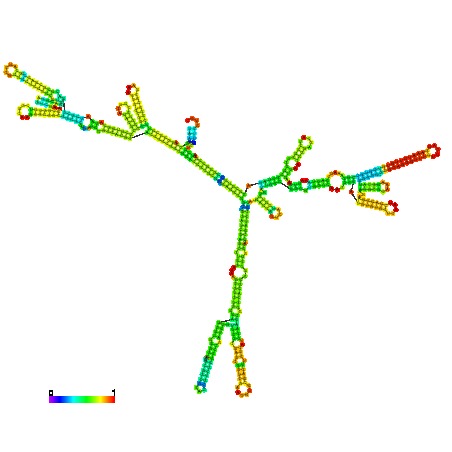 |
| 45 | BPNC20055R | Chr2 | 923485 | 923598 | 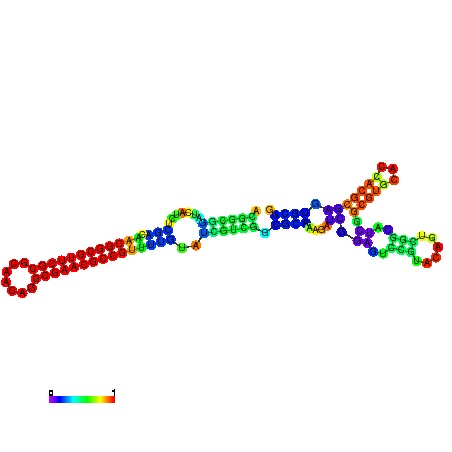 |
| 46 | BPNC20056R | Chr2 | 975704 | 975853 | 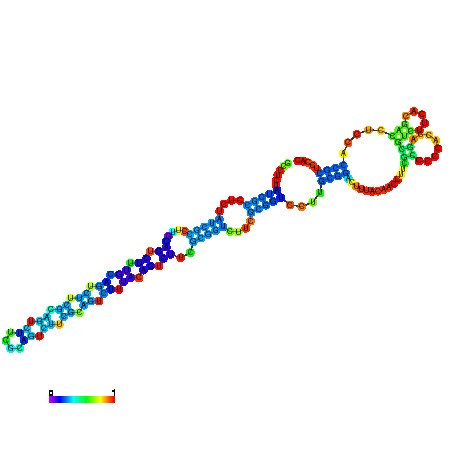 |
| 47 | BPNC20079R | Chr2 | 1289443 | 1289739 | 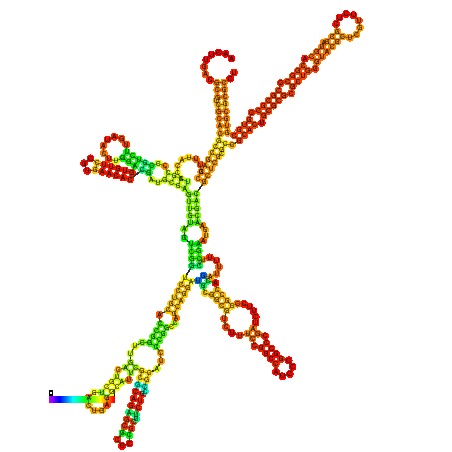 |
| 48 | BPNC20091F | Chr2 | 1245127 | 1245249 | 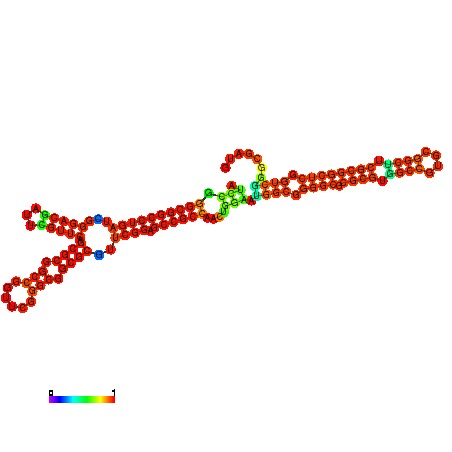 |
| 49 | BPNC20096F | Chr2 | 1382686 | 1382796 | 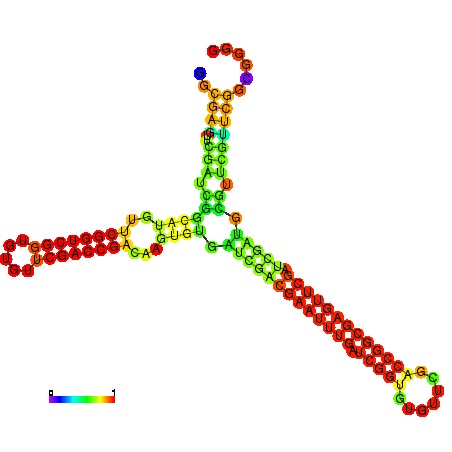 |
| 50 | BPNC20097F | Chr2 | 1382860 | 1383006 | 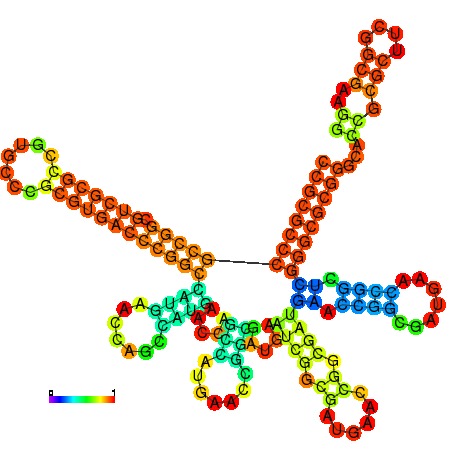 |
| 51 | BPNC20102F | Chr2 | 1553761 | 1553916 | 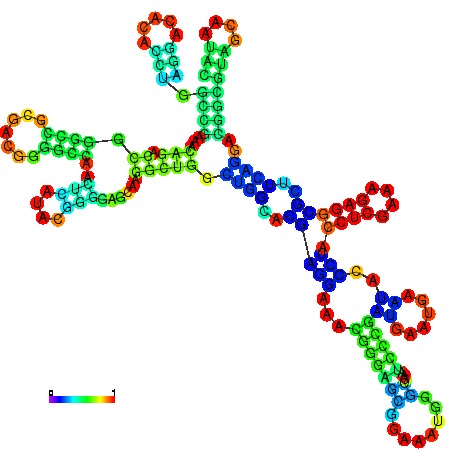 |
| 52 | BPNC20120F | Chr2 | 1826799 | 1826912 | 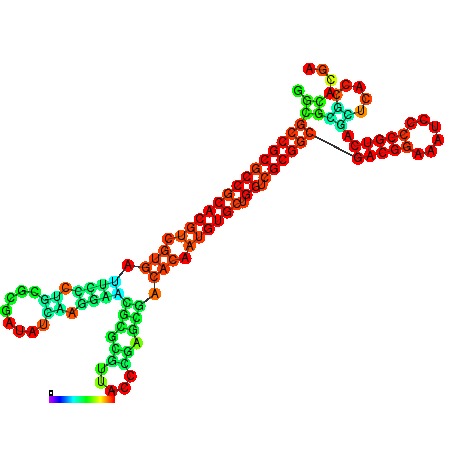 |
| 53 | BPNC20134R | Chr2 | 2376021 | 2376176 | 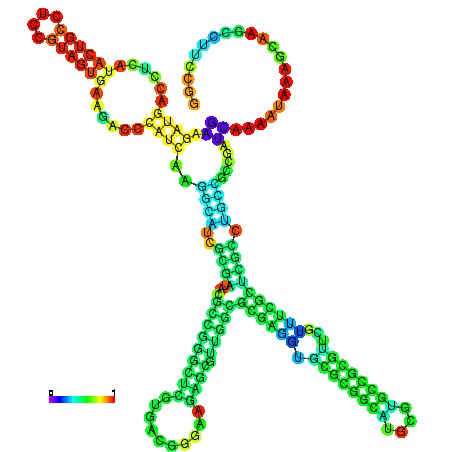 |
| 54 | BPNC20139R | Chr2 | 2432432 | 2432587 | 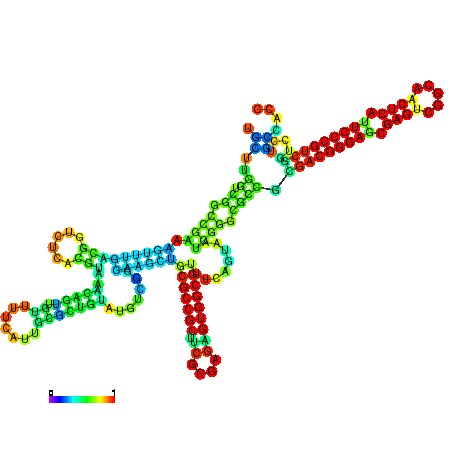 |
| 55 | BPNC20144R | Chr2 | 2506423 | 2506542 | 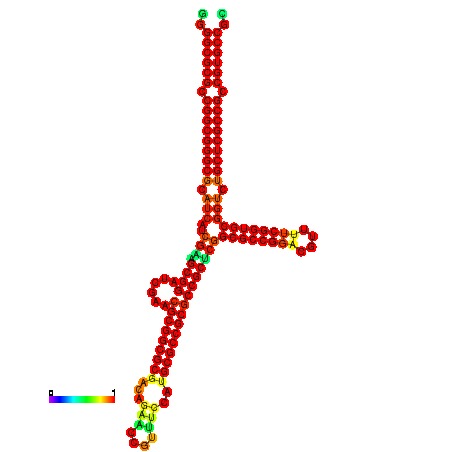 |
| 56 | BPNC20158R | Chr2 | 2854538 | 2854694 | 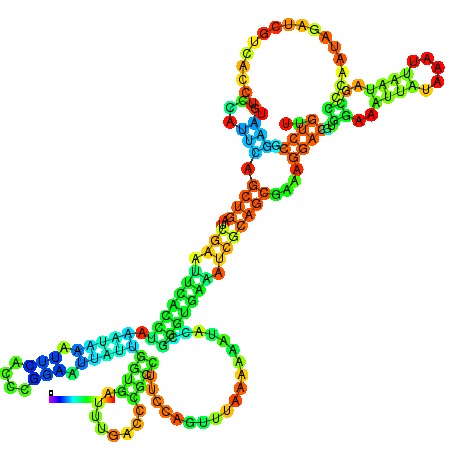 |
| 57 | BPNC20173F | Chr2 | 2901056 | 2901169 | 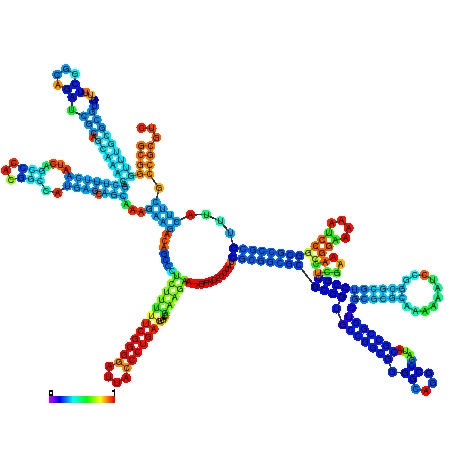 |
| 58 | BPNC20181R | Chr2 | 3122221 | 3122334 | 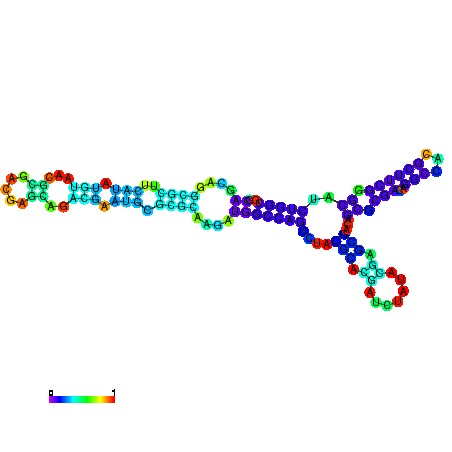 |
