# Supplementary material for: The Condition-Dependent Transcriptional Landscape of Burkholderia pseudomallei
Source: PLoS Genet. 2013 Sep 12;9(9):e1003795. doi: 10.1371/journal.pgen.1003795 (PMC3772027; doi:10.1371/journal.pgen.1003795)
Supplement: Table S9 — Correlation between ncRNAs and clusters. (DOC) [file pgen.1003795.s017.doc]

Table S9: Correlation between ncRNAs and clusters.

| **Feature** | **Cluster** | **Pearson’s Correlation Coefficient** |
| --- | --- | --- |
| BPNC10001F | C224 | 0.76 |
| BPNC10001F | C351 | 0.77 |
| BPNC10003R | C296 | -0.59 |
| BPNC10004F | C482 | 0.92 |
| BPNC10005F | C482 | 0.94 |
| BPNC10007F | C053 | 0.75 |
| BPNC10007F | C232 | 0.75 |
| BPNC10007R | C002 | 0.75 |
| BPNC10007R | C008 | 0.75 |
| BPNC10007R | C109 | 0.76 |
| BPNC10007R | C325 | 0.80 |
| BPNC10007R | C371 | 0.75 |
| BPNC10007R | C486 | 0.76 |
| BPNC10007R | C522 | 0.75 |
| BPNC10008F | C110 | 0.76 |
| BPNC10008R | C127 | 0.83 |
| BPNC10008R | C344 | 0.76 |
| BPNC10008R | C422 | 0.78 |
| BPNC10010R | C613 | -0.72 |
| BPNC10012R | C120 | 0.81 |
| BPNC10016F | C128 | -0.62 |
| BPNC10016F | C015 | 0.83 |
| BPNC10016F | C334 | 0.76 |
| BPNC10016F | C530 | 0.77 |
| BPNC10016F | C531 | 0.83 |
| BPNC10016R | C341 | 0.79 |
| BPNC10018R | C073 | 0.75 |
| BPNC10018R | C141 | 0.75 |
| BPNC10018R | C465 | 0.80 |
| BPNC10023R | C021 | 0.94 |
| BPNC10023R | C058 | 0.93 |
| BPNC10024R | C021 | 0.88 |
| BPNC10024R | C058 | 0.90 |
| BPNC10025F | C279 | 0.79 |
| BPNC10025F | C414 | 0.80 |
| BPNC10025R | C030 | 0.80 |
| BPNC10026F | C186 | 0.94 |
| BPNC10026F | C244 | 0.82 |
| BPNC10030R | C478 | -0.61 |
| BPNC10031F | C226 | 0.91 |
| BPNC10031F | C521 | 0.76 |
| BPNC10032R | C114 | 0.77 |
| BPNC10033F | C226 | 0.81 |
| BPNC10033R | C407 | -0.60 |
| BPNC10034R | C407 | -0.66 |
| BPNC10036R | C034 | 0.75 |
| BPNC10037F | C083 | 0.78 |
| BPNC10037F | C084 | 0.85 |
| BPNC10037F | C213 | 0.77 |
| BPNC10037F | C327 | 0.79 |
| BPNC10037R | C034 | 0.78 |
| BPNC10043R | C226 | 0.76 |
| BPNC10044R | C224 | 0.78 |
| BPNC10044R | C521 | 0.75 |
| BPNC10045F | C020 | 0.88 |
| BPNC10045F | C050 | 0.79 |
| BPNC10045F | C052 | 0.77 |
| BPNC10045R | C429 | -0.59 |
| BPNC10045R | C096 | -0.60 |
| BPNC10045R | C576 | -0.61 |
| BPNC10045R | C341 | -0.62 |
| BPNC10045R | C504 | -0.62 |
| BPNC10045R | C126 | -0.65 |
| BPNC10045R | C550 | -0.66 |
| BPNC10045R | C247 | -0.69 |
| BPNC10045R | C394 | -0.76 |
| BPNC10045R | C128 | 0.76 |
| BPNC10045R | C160 | 0.83 |
| BPNC10047F | C053 | 0.78 |
| BPNC10047F | C195 | 0.91 |
| BPNC10047F | C292 | 0.81 |
| BPNC10047F | C374 | 0.75 |
| BPNC10047F | C376 | 0.78 |
| BPNC10047F | C509 | 0.75 |
| BPNC10047F | C569 | 0.80 |
| BPNC10048R | C014 | 0.76 |
| BPNC10049F | C315 | 0.88 |
| BPNC10051F | C521 | 0.77 |
| BPNC10051F | C528 | 0.75 |
| BPNC10051F | C570 | 0.80 |
| BPNC10052R | C110 | 0.76 |
| BPNC10052R | C229 | 0.83 |
| BPNC10053F | C007 | 0.80 |
| BPNC10053F | C014 | 0.77 |
| BPNC10053F | C109 | 0.84 |
| BPNC10053F | C120 | 0.78 |
| BPNC10053F | C486 | 0.79 |
| BPNC10053F | C517 | 0.80 |
| BPNC10053R | C053 | 0.79 |
| BPNC10053R | C226 | 0.79 |
| BPNC10055F | C497 | 0.75 |
| BPNC10056F | C053 | 0.84 |
| BPNC10056F | C110 | 0.76 |
| BPNC10056F | C292 | 0.82 |
| BPNC10056F | C374 | 0.79 |
| BPNC10056F | C376 | 0.79 |
| BPNC10056F | C509 | 0.86 |
| BPNC10056R | C413 | -0.64 |
| BPNC10056R | C014 | 0.75 |
| BPNC10056R | C109 | 0.79 |
| BPNC10056R | C517 | 0.75 |
| BPNC10057F | C053 | 0.75 |
| BPNC10057F | C509 | 0.78 |
| BPNC10057R | C007 | 0.77 |
| BPNC10058F | C521 | 0.84 |
| BPNC10058F | C528 | 0.85 |
| BPNC10059F | C435 | 0.79 |
| BPNC10059F | C521 | 0.79 |
| BPNC10059F | C528 | 0.86 |
| BPNC10060F | C076 | -0.64 |
| BPNC10060F | C141 | 0.75 |
| BPNC10060F | C213 | 0.80 |
| BPNC10060F | C327 | 0.79 |
| BPNC10060F | C352 | 0.75 |
| BPNC10060F | C380 | 0.77 |
| BPNC10060F | C465 | 0.80 |
| BPNC10061F | C224 | 0.79 |
| BPNC10061F | C546 | 0.77 |
| BPNC10062F | C076 | -0.62 |
| BPNC10062R | C007 | 0.83 |
| BPNC10062R | C120 | 0.78 |
| BPNC10062R | C517 | 0.78 |
| BPNC10063F | C255 | 0.89 |
| BPNC10063R | C053 | 0.80 |
| BPNC10063R | C185 | 0.77 |
| BPNC10063R | C509 | 0.75 |
| BPNC10064F | C150 | 0.78 |
| BPNC10064F | C378 | 0.77 |
| BPNC10064F | C550 | 0.78 |
| BPNC10064R | C229 | 0.75 |
| BPNC10065F | C013 | 0.75 |
| BPNC10065F | C101 | 0.78 |
| BPNC10065F | C117 | 0.77 |
| BPNC10065F | C152 | 0.75 |
| BPNC10065F | C465 | 0.75 |
| BPNC10065F | C521 | 0.82 |
| BPNC10065F | C557 | 0.78 |
| BPNC10070F | C020 | 0.77 |
| BPNC10070F | C052 | 0.83 |
| BPNC10070F | C168 | 0.97 |
| BPNC10072F | C128 | 0.75 |
| BPNC10072F | C435 | 0.77 |
| BPNC10072F | C436 | 0.81 |
| BPNC10072F | C521 | 0.76 |
| BPNC10072R | C119 | 0.79 |
| BPNC10074F | C374 | 0.77 |
| BPNC10074F | C509 | 0.76 |
| BPNC10076F | C066 | 0.75 |
| BPNC10076F | C534 | 0.81 |
| BPNC10076F | C581 | 0.86 |
| BPNC10076R | C066 | 0.79 |
| BPNC10077R | C362 | 0.75 |
| BPNC10078R | C109 | 0.76 |
| BPNC10078R | C331 | 0.77 |
| BPNC10078R | C362 | 0.77 |
| BPNC10079R | C007 | 0.78 |
| BPNC10079R | C014 | 0.81 |
| BPNC10079R | C018 | 0.75 |
| BPNC10079R | C120 | 0.78 |
| BPNC10079R | C378 | 0.82 |
| BPNC10079R | C486 | 0.76 |
| BPNC10080R | C296 | -0.66 |
| BPNC10082F | C154 | -0.60 |
| BPNC10083F | C333 | 0.77 |
| BPNC10083F | C378 | 0.78 |
| BPNC10083R | C168 | 0.86 |
| BPNC10084F | C155 | -0.59 |
| BPNC10084F | C076 | -0.60 |
| BPNC10084F | C117 | 0.80 |
| BPNC10085R | C053 | 0.82 |
| BPNC10085R | C110 | 0.80 |
| BPNC10085R | C195 | 0.78 |
| BPNC10085R | C203 | 0.76 |
| BPNC10085R | C229 | 0.82 |
| BPNC10085R | C292 | 0.78 |
| BPNC10085R | C376 | 0.77 |
| BPNC10085R | C407 | 0.78 |
| BPNC10085R | C509 | 0.82 |
| BPNC10086R | C114 | 0.77 |
| BPNC10087R | C379 | 0.77 |
| BPNC10088F | C597 | 0.79 |
| BPNC10088R | C027 | 0.77 |
| BPNC10088R | C201 | 0.81 |
| BPNC10088R | C416 | 0.82 |
| BPNC10088R | C449 | 0.80 |
| BPNC10088R | C461 | 0.78 |
| BPNC10090F | C351 | 0.75 |
| BPNC10090F | C534 | 0.77 |
| BPNC10090F | C543 | 0.76 |
| BPNC10091R | C005 | 0.80 |
| BPNC10091R | C127 | 0.77 |
| BPNC10091R | C128 | 0.84 |
| BPNC10091R | C129 | 0.79 |
| BPNC10091R | C136 | 0.76 |
| BPNC10091R | C297 | 0.77 |
| BPNC10091R | C415 | 0.76 |
| BPNC10091R | C435 | 0.75 |
| BPNC10091R | C521 | 0.78 |
| BPNC10091R | C528 | 0.76 |
| BPNC10092R | C571 | -0.60 |
| BPNC10092R | C126 | 0.78 |
| BPNC10092R | C131 | 0.80 |
| BPNC10092R | C265 | 0.77 |
| BPNC10092R | C524 | 0.76 |
| BPNC10094R | C361 | -0.60 |
| BPNC10095R | C127 | 0.75 |
| BPNC10097F | C224 | 0.79 |
| BPNC10097F | C303 | 0.78 |
| BPNC10097F | C534 | 0.80 |
| BPNC10097F | C543 | 0.81 |
| BPNC10097R | C230 | -0.60 |
| BPNC10097R | C312 | -0.61 |
| BPNC10097R | C096 | -0.62 |
| BPNC10097R | C005 | 0.75 |
| BPNC10097R | C127 | 0.79 |
| BPNC10097R | C493 | 0.79 |
| BPNC10097R | C521 | 0.78 |
| BPNC10097R | C543 | 0.75 |
| BPNC10099R | C015 | -0.59 |
| BPNC10099R | C068 | -0.61 |
| BPNC10099R | C128 | 0.81 |
| BPNC10099R | C521 | 0.76 |
| BPNC10102F | C274 | 0.75 |
| BPNC10102F | C352 | 0.76 |
| BPNC10102F | C378 | 0.75 |
| BPNC10102F | C380 | 0.78 |
| BPNC10102F | C486 | 0.78 |
| BPNC10102R | C595 | -0.60 |
| BPNC10105F | C429 | 0.76 |
| BPNC10106F | C465 | 0.78 |
| BPNC10106R | C168 | -0.68 |
| BPNC10106R | C265 | 0.79 |
| BPNC10107F | C312 | 0.80 |
| BPNC10107R | C448 | 0.76 |
| BPNC10108F | C159 | 0.76 |
| BPNC10108F | C419 | 0.75 |
| BPNC10108R | C024 | -0.60 |
| BPNC10108R | C230 | -0.61 |
| BPNC10108R | C369 | -0.62 |
| BPNC10108R | C394 | -0.67 |
| BPNC10108R | C096 | -0.68 |
| BPNC10108R | C493 | 0.81 |
| BPNC10108R | C521 | 0.79 |
| BPNC10109F | C096 | -0.60 |
| BPNC10109F | C005 | 0.80 |
| BPNC10109F | C493 | 0.79 |
| BPNC10109F | C521 | 0.86 |
| BPNC10109F | C528 | 0.78 |
| BPNC10109F | C546 | 0.77 |
| BPNC10109R | C128 | 0.81 |
| BPNC10109R | C521 | 0.76 |
| BPNC10110R | C146 | -0.59 |
| BPNC10110R | C149 | -0.64 |
| BPNC10111F | C384 | 0.79 |
| BPNC10111R | C226 | 0.78 |
| BPNC10112R | C371 | -0.59 |
| BPNC10113R | C319 | 0.76 |
| BPNC10114F | C053 | 0.76 |
| BPNC10114F | C185 | 0.75 |
| BPNC10114F | C226 | 0.79 |
| BPNC10116F | C344 | 0.75 |
| BPNC10116F | C534 | 0.77 |
| BPNC10116R | C586 | 0.88 |
| BPNC10118R | C465 | 0.77 |
| BPNC10119R | C027 | 0.89 |
| BPNC10119R | C201 | 0.94 |
| BPNC10119R | C416 | 0.95 |
| BPNC10119R | C449 | 0.92 |
| BPNC10119R | C461 | 0.88 |
| BPNC10120F | C465 | 0.78 |
| BPNC10120R | C534 | 0.79 |
| BPNC10120R | C581 | 0.86 |
| BPNC10121F | C524 | -0.59 |
| BPNC10121F | C553 | -0.59 |
| BPNC10121F | C126 | -0.62 |
| BPNC10121F | C462 | -0.64 |
| BPNC10121F | C080 | 0.83 |
| BPNC10121F | C400 | 0.78 |
| BPNC10121F | C446 | 0.84 |
| BPNC10121F | C447 | 0.76 |
| BPNC10121F | C448 | 0.78 |
| BPNC10122F | C080 | 0.81 |
| BPNC10122F | C400 | 0.77 |
| BPNC10122F | C446 | 0.86 |
| BPNC10122F | C447 | 0.78 |
| BPNC10122F | C448 | 0.76 |
| BPNC10123R | C127 | 0.75 |
| BPNC10123R | C303 | 0.75 |
| BPNC10123R | C422 | 0.75 |
| BPNC10123R | C534 | 0.79 |
| BPNC10123R | C543 | 0.76 |
| BPNC10124R | C005 | 0.75 |
| BPNC10124R | C013 | 0.76 |
| BPNC10124R | C478 | 0.77 |
| BPNC10124R | C493 | 0.81 |
| BPNC10124R | C521 | 0.76 |
| BPNC10124R | C546 | 0.77 |
| BPNC10125F | C007 | 0.75 |
| BPNC10126F | C224 | 0.83 |
| BPNC10126F | C303 | 0.80 |
| BPNC10126F | C543 | 0.79 |
| BPNC10126R | C362 | 0.79 |
| BPNC10127F | C007 | 0.76 |
| BPNC10127R | C465 | 0.76 |
| BPNC10129F | C373 | -0.59 |
| BPNC10129R | C127 | 0.81 |
| BPNC10129R | C528 | 0.83 |
| BPNC10130F | C054 | 0.79 |
| BPNC10130F | C619 | 0.76 |
| BPNC10131F | C123 | -0.60 |
| BPNC10131F | C154 | -0.63 |
| BPNC10131F | C054 | 0.81 |
| BPNC10131F | C571 | 0.75 |
| BPNC10131F | C591 | 0.84 |
| BPNC10132F | C005 | 0.80 |
| BPNC10132F | C127 | 0.78 |
| BPNC10132F | C128 | 0.82 |
| BPNC10132F | C224 | 0.76 |
| BPNC10132F | C415 | 0.77 |
| BPNC10132F | C436 | 0.75 |
| BPNC10132F | C521 | 0.80 |
| BPNC10132F | C528 | 0.78 |
| BPNC10132F | C546 | 0.75 |
| BPNC10132R | C015 | 0.77 |
| BPNC10132R | C530 | 0.81 |
| BPNC10132R | C531 | 0.82 |
| BPNC10133F | C096 | -0.65 |
| BPNC10133F | C394 | -0.69 |
| BPNC10133F | C128 | 0.80 |
| BPNC10133F | C435 | 0.78 |
| BPNC10133F | C521 | 0.77 |
| BPNC10134R | C066 | 0.76 |
| BPNC10135R | C559 | -0.59 |
| BPNC10135R | C146 | 0.80 |
| BPNC10135R | C259 | 0.82 |
| BPNC10135R | C468 | 0.79 |
| BPNC10136F | C185 | 0.76 |
| BPNC10138F | C224 | 0.79 |
| BPNC10138F | C236 | 0.76 |
| BPNC10139R | C382 | 0.75 |
| BPNC10140R | C066 | 0.76 |
| BPNC10140R | C534 | 0.75 |
| BPNC10140R | C581 | 0.84 |
| BPNC10141F | C096 | -0.62 |
| BPNC10141F | C394 | -0.66 |
| BPNC10141F | C005 | 0.79 |
| BPNC10141F | C128 | 0.80 |
| BPNC10141F | C493 | 0.77 |
| BPNC10141F | C546 | 0.81 |
| BPNC10141R | C591 | 0.75 |
| BPNC10142F | C394 | -0.67 |
| BPNC10142F | C096 | -0.69 |
| BPNC10142F | C005 | 0.81 |
| BPNC10142F | C128 | 0.81 |
| BPNC10142F | C435 | 0.75 |
| BPNC10142F | C436 | 0.76 |
| BPNC10142F | C493 | 0.80 |
| BPNC10142F | C521 | 0.79 |
| BPNC10142F | C546 | 0.78 |
| BPNC10143F | C121 | 0.75 |
| BPNC10143F | C497 | 0.77 |
| BPNC10144F | C296 | -0.65 |
| BPNC10144F | C154 | -0.66 |
| BPNC10144F | C076 | -0.67 |
| BPNC10145F | C001 | -0.59 |
| BPNC10145F | C296 | -0.66 |
| BPNC10145F | C076 | -0.73 |
| BPNC10145R | C054 | 0.80 |
| BPNC10145R | C571 | 0.81 |
| BPNC10145R | C619 | 0.76 |
| BPNC10146F | C413 | -0.59 |
| BPNC10146F | C002 | 0.75 |
| BPNC10146F | C014 | 0.78 |
| BPNC10146F | C109 | 0.80 |
| BPNC10146F | C362 | 0.77 |
| BPNC10146R | C003 | 0.80 |
| BPNC10146R | C007 | 0.77 |
| BPNC10146R | C014 | 0.78 |
| BPNC10146R | C517 | 0.78 |
| BPNC10149F | C036 | 0.80 |
| BPNC10149F | C608 | 0.80 |
| BPNC10150F | C036 | 0.90 |
| BPNC10150F | C608 | 0.92 |
| BPNC10151R | C362 | 0.78 |
| BPNC10152F | C066 | 0.82 |
| BPNC10152F | C581 | 0.82 |
| BPNC10152R | C350 | 0.77 |
| BPNC10153F | C230 | -0.59 |
| BPNC10153F | C369 | -0.60 |
| BPNC10153F | C123 | -0.62 |
| BPNC10153F | C394 | -0.62 |
| BPNC10153F | C096 | -0.65 |
| BPNC10157F | C066 | 0.78 |
| BPNC10157F | C581 | 0.77 |
| BPNC10158R | C159 | 0.75 |
| BPNC10160F | C080 | 0.79 |
| BPNC10160F | C386 | 0.76 |
| BPNC10160F | C446 | 0.86 |
| BPNC10160F | C447 | 0.76 |
| BPNC10160F | C448 | 0.80 |
| BPNC10162F | C007 | 0.82 |
| BPNC10162F | C109 | 0.78 |
| BPNC10162F | C517 | 0.76 |
| BPNC10162R | C351 | 0.78 |
| BPNC10162R | C581 | 0.88 |
| BPNC10163R | C543 | 0.76 |
| BPNC10164R | C236 | 0.76 |
| BPNC10164R | C521 | 0.78 |
| BPNC10165F | C589 | 0.83 |
| BPNC10166F | C109 | 0.78 |
| BPNC10169F | C624 | -0.60 |
| BPNC10169F | C081 | -0.61 |
| BPNC10169F | C413 | -0.62 |
| BPNC10169F | C588 | -0.65 |
| BPNC10169R | C068 | -0.60 |
| BPNC10169R | C127 | 0.77 |
| BPNC10170R | C186 | 0.87 |
| BPNC10170R | C244 | 0.80 |
| BPNC10171R | C556 | 0.76 |
| BPNC10172R | C120 | 0.81 |
| BPNC10172R | C325 | 0.78 |
| BPNC10176F | C090 | 0.77 |
| BPNC10176R | C119 | 0.89 |
| BPNC10177F | C076 | -0.60 |
| BPNC10177F | C296 | -0.61 |
| BPNC10177R | C362 | 0.77 |
| BPNC10178F | C073 | 0.75 |
| BPNC10179R | C076 | -0.59 |
| BPNC10180F | C154 | -0.59 |
| BPNC10180F | C271 | -0.60 |
| BPNC10180F | C076 | -0.62 |
| BPNC10181F | C115 | 0.77 |
| BPNC10181F | C429 | 0.80 |
| BPNC10181R | C213 | 0.77 |
| BPNC10181R | C465 | 0.75 |
| BPNC10182R | C087 | -0.60 |
| BPNC10183R | C146 | 0.76 |
| BPNC10184F | C588 | -0.59 |
| BPNC10184R | C340 | 0.77 |
| BPNC10185R | C445 | 0.92 |
| BPNC10186F | C066 | 0.77 |
| BPNC10186R | C445 | 0.89 |
| BPNC10188F | C127 | 0.79 |
| BPNC10188R | C426 | 0.77 |
| BPNC10188R | C427 | 0.76 |
| BPNC10190F | C045 | 0.76 |
| BPNC10190F | C246 | 0.77 |
| BPNC10190R | C073 | 0.78 |
| BPNC10190R | C109 | 0.76 |
| BPNC10190R | C176 | 0.76 |
| BPNC10190R | C331 | 0.75 |
| BPNC10190R | C362 | 0.75 |
| BPNC10191R | C174 | 0.86 |
| BPNC10191R | C205 | 0.78 |
| BPNC10191R | C440 | 0.88 |
| BPNC10192F | C054 | 0.90 |
| BPNC10192F | C139 | 0.89 |
| BPNC10192F | C571 | 0.81 |
| BPNC10192F | C615 | 0.76 |
| BPNC10192F | C619 | 0.82 |
| BPNC10192R | C296 | -0.66 |
| BPNC10193F | C054 | 0.82 |
| BPNC10193F | C139 | 0.87 |
| BPNC10193F | C615 | 0.86 |
| BPNC10194F | C382 | 0.84 |
| BPNC10195F | C510 | 0.80 |
| BPNC10195R | C083 | 0.77 |
| BPNC10195R | C274 | 0.77 |
| BPNC10195R | C486 | 0.80 |
| BPNC10196F | C084 | 0.79 |
| BPNC10196F | C497 | 0.78 |
| BPNC10196R | C045 | 0.75 |
| BPNC10196R | C114 | 0.77 |
| BPNC10196R | C130 | 0.81 |
| BPNC10196R | C196 | 0.75 |
| BPNC10196R | C205 | 0.76 |
| BPNC10197F | C483 | -0.62 |
| BPNC10197F | C299 | -0.62 |
| BPNC10197R | C319 | -0.60 |
| BPNC10197R | C614 | 0.91 |
| BPNC10198R | C614 | 0.95 |
| BPNC10200R | C084 | 0.76 |
| BPNC10200R | C101 | 0.76 |
| BPNC10200R | C497 | 0.76 |
| BPNC10201F | C303 | 0.80 |
| BPNC10201F | C422 | 0.79 |
| BPNC10201F | C534 | 0.76 |
| BPNC10202F | C446 | -0.59 |
| BPNC10202R | C109 | 0.77 |
| BPNC10203F | C271 | -0.59 |
| BPNC10204F | C296 | -0.60 |
| BPNC10207R | C053 | 0.77 |
| BPNC10207R | C110 | 0.83 |
| BPNC10207R | C195 | 0.78 |
| BPNC10207R | C229 | 0.83 |
| BPNC10207R | C282 | 0.81 |
| BPNC10207R | C292 | 0.76 |
| BPNC10207R | C375 | 0.79 |
| BPNC10207R | C376 | 0.77 |
| BPNC10207R | C407 | 0.77 |
| BPNC10207R | C509 | 0.80 |
| BPNC10207R | C510 | 0.83 |
| BPNC10207R | C511 | 0.76 |
| BPNC10208R | C081 | -0.59 |
| BPNC10208R | C559 | -0.63 |
| BPNC10208R | C519 | 0.76 |
| BPNC10209R | C003 | 0.80 |
| BPNC10209R | C149 | 0.78 |
| BPNC10209R | C380 | 0.77 |
| BPNC10209R | C519 | 0.78 |
| BPNC10209R | C533 | 0.79 |
| BPNC10210R | C083 | 0.75 |
| BPNC10210R | C141 | 0.77 |
| BPNC10210R | C465 | 0.86 |
| BPNC10212R | C160 | -0.61 |
| BPNC10212R | C554 | -0.64 |
| BPNC10212R | C126 | 0.87 |
| BPNC10212R | C524 | 0.81 |
| BPNC10212R | C553 | 0.79 |
| BPNC10213R | C447 | -0.60 |
| BPNC10213R | C403 | -0.60 |
| BPNC10213R | C160 | -0.60 |
| BPNC10213R | C361 | -0.61 |
| BPNC10213R | C448 | -0.62 |
| BPNC10213R | C245 | -0.63 |
| BPNC10213R | C400 | -0.65 |
| BPNC10213R | C571 | -0.65 |
| BPNC10213R | C446 | -0.65 |
| BPNC10213R | C080 | -0.66 |
| BPNC10213R | C554 | -0.68 |
| BPNC10213R | C126 | 0.88 |
| BPNC10213R | C524 | 0.86 |
| BPNC10213R | C553 | 0.81 |
| BPNC10215R | C486 | 0.78 |
| BPNC10217R | C101 | 0.77 |
| BPNC10217R | C192 | 0.75 |
| BPNC10220R | C150 | 0.76 |
| BPNC10220R | C259 | 0.79 |
| BPNC10220R | C333 | 0.79 |
| BPNC10220R | C334 | 0.77 |
| BPNC10220R | C378 | 0.79 |
| BPNC10225R | C007 | 0.83 |
| BPNC10225R | C014 | 0.79 |
| BPNC10225R | C018 | 0.75 |
| BPNC10225R | C109 | 0.76 |
| BPNC10225R | C120 | 0.76 |
| BPNC10225R | C517 | 0.76 |
| BPNC10227R | C120 | 0.76 |
| BPNC10228R | C110 | 0.85 |
| BPNC10228R | C195 | 0.78 |
| BPNC10228R | C229 | 0.86 |
| BPNC10228R | C282 | 0.86 |
| BPNC10228R | C292 | 0.78 |
| BPNC10228R | C407 | 0.76 |
| BPNC10228R | C510 | 0.82 |
| BPNC10228R | C511 | 0.81 |
| BPNC10228R | C568 | 0.79 |
| BPNC10229R | C195 | 0.75 |
| BPNC10229R | C510 | 0.79 |
| BPNC10229R | C568 | 0.84 |
| BPNC10234R | C076 | -0.63 |
| BPNC10234R | C465 | 0.78 |
| BPNC20001F | C216 | 0.79 |
| BPNC20001F | C486 | 0.75 |
| BPNC20002F | C094 | -0.59 |
| BPNC20003F | C533 | -0.62 |
| BPNC20003R | C465 | -0.60 |
| BPNC20003R | C327 | -0.61 |
| BPNC20003R | C165 | -0.63 |
| BPNC20003R | C196 | -0.64 |
| BPNC20003R | C313 | -0.65 |
| BPNC20004F | C229 | 0.75 |
| BPNC20004F | C283 | 0.85 |
| BPNC20004F | C292 | 0.77 |
| BPNC20004F | C376 | 0.78 |
| BPNC20004F | C509 | 0.81 |
| BPNC20009F | C027 | 0.93 |
| BPNC20009F | C201 | 0.79 |
| BPNC20009F | C416 | 0.90 |
| BPNC20009F | C449 | 0.91 |
| BPNC20009F | C461 | 0.97 |
| BPNC20010F | C534 | 0.77 |
| BPNC20010R | C229 | 0.78 |
| BPNC20010R | C283 | 0.84 |
| BPNC20010R | C292 | 0.76 |
| BPNC20010R | C509 | 0.75 |
| BPNC20011R | C053 | 0.80 |
| BPNC20011R | C110 | 0.77 |
| BPNC20011R | C374 | 0.77 |
| BPNC20011R | C509 | 0.81 |
| BPNC20012R | C468 | -0.59 |
| BPNC20013F | C247 | -0.61 |
| BPNC20013F | C394 | -0.66 |
| BPNC20013F | C005 | 0.75 |
| BPNC20013F | C045 | 0.79 |
| BPNC20013F | C128 | 0.83 |
| BPNC20013F | C136 | 0.80 |
| BPNC20013F | C160 | 0.76 |
| BPNC20013F | C297 | 0.76 |
| BPNC20013F | C415 | 0.76 |
| BPNC20014F | C020 | 0.82 |
| BPNC20014F | C050 | 0.80 |
| BPNC20014F | C052 | 0.77 |
| BPNC20014F | C168 | 0.77 |
| BPNC20016F | C027 | 0.88 |
| BPNC20016F | C201 | 0.87 |
| BPNC20016F | C416 | 0.92 |
| BPNC20016F | C449 | 0.92 |
| BPNC20016F | C461 | 0.89 |
| BPNC20017F | C247 | -0.61 |
| BPNC20018F | C132 | 0.78 |
| BPNC20018F | C389 | 0.86 |
| BPNC20019F | C361 | -0.61 |
| BPNC20019R | C414 | 0.81 |
| BPNC20020R | C027 | 0.82 |
| BPNC20020R | C201 | 0.78 |
| BPNC20020R | C416 | 0.85 |
| BPNC20020R | C449 | 0.84 |
| BPNC20020R | C461 | 0.85 |
| BPNC20023F | C296 | -0.61 |
| BPNC20023F | C076 | -0.61 |
| BPNC20024F | C068 | 0.76 |
| BPNC20024R | C230 | -0.61 |
| BPNC20024R | C096 | -0.68 |
| BPNC20025F | C068 | 0.79 |
| BPNC20027F | C458 | 0.91 |
| BPNC20027R | C435 | 0.92 |
| BPNC20028F | C042 | 0.78 |
| BPNC20028F | C045 | 0.84 |
| BPNC20028F | C114 | 0.83 |
| BPNC20028F | C136 | 0.77 |
| BPNC20028F | C246 | 0.77 |
| BPNC20028F | C297 | 0.77 |
| BPNC20030R | C068 | 0.84 |
| BPNC20031F | C226 | 0.78 |
| BPNC20031R | C068 | 0.95 |
| BPNC20032F | C053 | 0.77 |
| BPNC20032F | C226 | 0.86 |
| BPNC20033F | C307 | -0.60 |
| BPNC20033F | C203 | -0.62 |
| BPNC20033F | C282 | -0.62 |
| BPNC20033F | C376 | -0.63 |
| BPNC20033F | C407 | -0.63 |
| BPNC20033F | C511 | -0.64 |
| BPNC20033F | C053 | -0.65 |
| BPNC20033F | C568 | -0.65 |
| BPNC20033F | C227 | -0.67 |
| BPNC20033F | C292 | -0.67 |
| BPNC20033F | C510 | -0.68 |
| BPNC20033F | C569 | -0.68 |
| BPNC20033F | C509 | -0.70 |
| BPNC20033F | C195 | -0.71 |
| BPNC20033F | C229 | -0.71 |
| BPNC20033F | C374 | -0.74 |
| BPNC20033F | C110 | -0.75 |
| BPNC20033R | C591 | -0.61 |
| BPNC20033R | C615 | -0.61 |
| BPNC20033R | C168 | -0.64 |
| BPNC20034F | C136 | 0.76 |
| BPNC20034F | C192 | 0.81 |
| BPNC20034F | C297 | 0.75 |
| BPNC20034R | C160 | -0.62 |
| BPNC20035F | C045 | 0.76 |
| BPNC20035F | C136 | 0.84 |
| BPNC20036F | C350 | 0.77 |
| BPNC20036F | C465 | 0.78 |
| BPNC20036F | C557 | 0.78 |
| BPNC20038F | C096 | 0.82 |
| BPNC20040F | C101 | 0.76 |
| BPNC20040R | C155 | -0.60 |
| BPNC20040R | C154 | -0.66 |
| BPNC20040R | C448 | 0.76 |
| BPNC20040R | C561 | 0.78 |
| BPNC20041F | C504 | -0.60 |
| BPNC20041F | C553 | -0.60 |
| BPNC20041F | C154 | -0.60 |
| BPNC20041F | C126 | -0.61 |
| BPNC20041F | C394 | -0.62 |
| BPNC20041F | C080 | 0.79 |
| BPNC20041F | C400 | 0.77 |
| BPNC20041F | C446 | 0.77 |
| BPNC20041F | C447 | 0.79 |
| BPNC20041F | C448 | 0.81 |
| BPNC20041R | C115 | -0.61 |
| BPNC20041R | C429 | -0.67 |
| BPNC20041R | C394 | -0.70 |
| BPNC20041R | C554 | 0.78 |
| BPNC20046F | C127 | 0.79 |
| BPNC20046F | C422 | 0.80 |
| BPNC20049F | C252 | 0.88 |
| BPNC20050F | C027 | 0.94 |
| BPNC20050F | C201 | 0.85 |
| BPNC20050F | C416 | 0.94 |
| BPNC20050F | C449 | 0.94 |
| BPNC20050F | C461 | 0.97 |
| BPNC20050R | C620 | 0.79 |
| BPNC20051R | C136 | 0.76 |
| BPNC20051R | C419 | 0.75 |
| BPNC20052R | C154 | -0.61 |
| BPNC20052R | C394 | -0.64 |
| BPNC20053R | C247 | -0.59 |
| BPNC20053R | C394 | -0.67 |
| BPNC20054R | C029 | 0.79 |
| BPNC20054R | C393 | 0.78 |
| BPNC20056F | C621 | 0.88 |
| BPNC20057F | C621 | 0.80 |
| BPNC20057R | C535 | 0.77 |
| BPNC20058F | C620 | 0.80 |
| BPNC20058F | C621 | 0.78 |
| BPNC20059F | C620 | 0.75 |
| BPNC20059F | C621 | 0.79 |
| BPNC20060R | C394 | -0.66 |
| BPNC20060R | C097 | 0.78 |
| BPNC20060R | C114 | 0.75 |
| BPNC20060R | C128 | 0.78 |
| BPNC20060R | C160 | 0.76 |
| BPNC20060R | C192 | 0.76 |
| BPNC20060R | C240 | 0.75 |
| BPNC20060R | C521 | 0.80 |
| BPNC20061F | C620 | 0.80 |
| BPNC20061F | C621 | 0.78 |
| BPNC20062R | C185 | 0.76 |
| BPNC20063F | C620 | 0.85 |
| BPNC20063F | C621 | 0.78 |
| BPNC20063R | C593 | -0.59 |
| BPNC20063R | C165 | -0.60 |
| BPNC20063R | C313 | -0.60 |
| BPNC20063R | C465 | -0.61 |
| BPNC20063R | C468 | -0.63 |
| BPNC20065F | C622 | 0.80 |
| BPNC20065R | C210 | 0.78 |
| BPNC20065R | C322 | 0.82 |
| BPNC20066F | C029 | 0.88 |
| BPNC20066F | C393 | 0.76 |
| BPNC20067F | C029 | 0.91 |
| BPNC20067F | C114 | 0.75 |
| BPNC20067F | C393 | 0.80 |
| BPNC20068F | C394 | -0.63 |
| BPNC20068F | C546 | 0.80 |
| BPNC20072R | C101 | 0.75 |
| BPNC20074R | C027 | 0.83 |
| BPNC20074R | C201 | 0.85 |
| BPNC20074R | C416 | 0.87 |
| BPNC20074R | C449 | 0.85 |
| BPNC20074R | C461 | 0.82 |
| BPNC20076R | C465 | 0.78 |
| BPNC20080F | C467 | 0.80 |
| BPNC20081R | C531 | 0.77 |
| BPNC20081R | C536 | 0.77 |
| BPNC20082F | C045 | 0.79 |
| BPNC20082F | C114 | 0.82 |
| BPNC20085F | C128 | 0.76 |
| BPNC20085F | C236 | 0.79 |
| BPNC20087F | C575 | 0.75 |
| BPNC20088F | C027 | 0.92 |
| BPNC20088F | C201 | 0.92 |
| BPNC20088F | C416 | 0.96 |
| BPNC20088F | C449 | 0.95 |
| BPNC20088F | C461 | 0.93 |
| BPNC20089F | C014 | 0.77 |
| BPNC20089F | C378 | 0.82 |
| BPNC20089F | C486 | 0.78 |
| BPNC20089R | C002 | 0.76 |
| BPNC20089R | C007 | 0.77 |
| BPNC20089R | C014 | 0.76 |
| BPNC20089R | C109 | 0.79 |
| BPNC20089R | C378 | 0.79 |
| BPNC20089R | C517 | 0.76 |
| BPNC20093F | C374 | 0.76 |
| BPNC20093F | C376 | 0.78 |
| BPNC20093F | C509 | 0.76 |
| BPNC20097R | C247 | -0.59 |
| BPNC20097R | C029 | 0.80 |
| BPNC20097R | C045 | 0.81 |
| BPNC20097R | C114 | 0.76 |
| BPNC20097R | C136 | 0.79 |
| BPNC20097R | C246 | 0.75 |
| BPNC20098R | C247 | -0.60 |
| BPNC20099F | C045 | 0.78 |
| BPNC20099F | C097 | 0.80 |
| BPNC20099F | C114 | 0.79 |
| BPNC20099R | C394 | -0.60 |
| BPNC20099R | C117 | 0.80 |
| BPNC20099R | C478 | 0.79 |
| BPNC20099R | C493 | 0.79 |
| BPNC20099R | C521 | 0.82 |
| BPNC20099R | C546 | 0.81 |
| BPNC20100R | C005 | 0.75 |
| BPNC20100R | C101 | 0.75 |
| BPNC20100R | C117 | 0.82 |
| BPNC20100R | C160 | 0.76 |
| BPNC20100R | C478 | 0.81 |
| BPNC20100R | C493 | 0.76 |
| BPNC20100R | C521 | 0.81 |
| BPNC20100R | C546 | 0.81 |
| BPNC20100R | C557 | 0.76 |
| BPNC20101R | C597 | 0.82 |
| BPNC20103F | C521 | -0.60 |
| BPNC20103F | C024 | 0.84 |
| BPNC20103F | C096 | 0.81 |
| BPNC20104F | C024 | 0.85 |
| BPNC20104F | C336 | 0.82 |
| BPNC20104R | C235 | 0.75 |
| BPNC20104R | C561 | 0.83 |
| BPNC20107F | C420 | 0.79 |
| BPNC20107R | C138 | 0.96 |
| BPNC20109R | C155 | -0.60 |
| BPNC20109R | C076 | -0.65 |
| BPNC20110F | C296 | -0.61 |
| BPNC20110F | C076 | -0.62 |
| BPNC20111F | C076 | -0.71 |
| BPNC20111F | C465 | 0.77 |
| BPNC20112R | C266 | 0.88 |
| BPNC20114R | C436 | 0.76 |
| BPNC20114R | C493 | 0.75 |
| BPNC20114R | C521 | 0.82 |
| BPNC20117F | C127 | 0.80 |
| BPNC20117F | C528 | 0.79 |
| BPNC20120R | C030 | 0.89 |
| BPNC20121F | C266 | 0.84 |
| BPNC20122F | C581 | 0.85 |
| BPNC20122R | C030 | 0.88 |
| BPNC20122R | C035 | 0.77 |
| BPNC20124F | C027 | 0.85 |
| BPNC20124F | C201 | 0.94 |
| BPNC20124F | C416 | 0.92 |
| BPNC20124F | C449 | 0.89 |
| BPNC20124F | C461 | 0.85 |
| BPNC20124R | C096 | -0.64 |
| BPNC20124R | C394 | -0.65 |
| BPNC20124R | C005 | 0.76 |
| BPNC20124R | C478 | 0.76 |
| BPNC20124R | C493 | 0.76 |
| BPNC20124R | C521 | 0.84 |
| BPNC20124R | C546 | 0.78 |
| BPNC20126R | C018 | 0.78 |
| BPNC20128R | C246 | 0.81 |
| BPNC20128R | C403 | 0.76 |
| BPNC20129R | C096 | -0.60 |
| BPNC20129R | C394 | -0.66 |
| BPNC20129R | C005 | 0.83 |
| BPNC20129R | C128 | 0.85 |
| BPNC20129R | C136 | 0.75 |
| BPNC20129R | C435 | 0.77 |
| BPNC20129R | C436 | 0.80 |
| BPNC20129R | C493 | 0.77 |
| BPNC20129R | C521 | 0.87 |
| BPNC20129R | C546 | 0.75 |
| BPNC20131R | C449 | 0.77 |
| BPNC20131R | C461 | 0.76 |
| BPNC20132R | C029 | 0.90 |
| BPNC20132R | C045 | 0.84 |
| BPNC20132R | C114 | 0.77 |
| BPNC20132R | C136 | 0.79 |
| BPNC20132R | C246 | 0.78 |
| BPNC20135F | C030 | 0.89 |
| BPNC20135F | C035 | 0.82 |
| BPNC20135R | C363 | 0.75 |
| BPNC20136F | C436 | 0.77 |
| BPNC20138F | C168 | -0.62 |
| BPNC20138R | C296 | -0.60 |
| BPNC20139F | C414 | 0.82 |
| BPNC20142R | C116 | 0.81 |
| BPNC20145F | C045 | 0.75 |
| BPNC20146F | C296 | -0.60 |
| BPNC20146F | C154 | -0.62 |
| BPNC20146F | C421 | -0.64 |
| BPNC20147F | C018 | 0.78 |
| BPNC20147F | C120 | 0.77 |
| BPNC20147R | C097 | 0.80 |
| BPNC20147R | C114 | 0.77 |
| BPNC20149R | C113 | 0.76 |
| BPNC20149R | C437 | 0.78 |
| BPNC20151F | C517 | 0.76 |
| BPNC20151R | C069 | 0.76 |
| BPNC20151R | C113 | 0.81 |
| BPNC20151R | C437 | 0.76 |
| BPNC20152F | C528 | 0.77 |
| BPNC20153R | C230 | -0.60 |
| BPNC20153R | C236 | 0.78 |
| BPNC20153R | C594 | 0.76 |
| BPNC20154F | C504 | -0.60 |
| BPNC20154F | C219 | -0.61 |
| BPNC20154F | C368 | -0.63 |
| BPNC20154R | C160 | -0.59 |
| BPNC20155R | C110 | 0.82 |
| BPNC20155R | C229 | 0.81 |
| BPNC20155R | C375 | 0.79 |
| BPNC20155R | C509 | 0.81 |
| BPNC20155R | C510 | 0.80 |
| BPNC20156F | C069 | 0.78 |
| BPNC20156F | C092 | 0.79 |
| BPNC20156F | C113 | 0.86 |
| BPNC20156F | C132 | 0.84 |
| BPNC20156F | C389 | 0.84 |
| BPNC20156F | C539 | 0.76 |
| BPNC20156R | C436 | 0.76 |
| BPNC20156R | C521 | 0.82 |
| BPNC20157F | C132 | 0.76 |
| BPNC20157R | C096 | -0.63 |
| BPNC20157R | C394 | -0.66 |
| BPNC20157R | C005 | 0.78 |
| BPNC20157R | C128 | 0.81 |
| BPNC20157R | C493 | 0.79 |
| BPNC20157R | C521 | 0.93 |
| BPNC20157R | C528 | 0.75 |
| BPNC20157R | C546 | 0.77 |
| BPNC20158R | C050 | 0.82 |
| BPNC20158R | C521 | 0.77 |
| BPNC20159R | C521 | 0.84 |
| BPNC20161F | C412 | 0.83 |
| BPNC20161R | C603 | 0.77 |
| BPNC20163F | C394 | -0.62 |
| BPNC20163F | C123 | -0.63 |
| BPNC20163F | C446 | 0.75 |
| BPNC20163R | C556 | 0.75 |
| BPNC20163R | C570 | 0.76 |
| BPNC20164R | C209 | 0.90 |
| BPNC20165F | C005 | 0.81 |
| BPNC20165F | C127 | 0.78 |
| BPNC20165F | C128 | 0.82 |
| BPNC20165F | C129 | 0.78 |
| BPNC20165F | C521 | 0.83 |
| BPNC20165F | C528 | 0.76 |
| BPNC20165F | C546 | 0.76 |
| BPNC20165R | C096 | -0.61 |
| BPNC20165R | C436 | 0.80 |
| BPNC20165R | C521 | 0.83 |
| BPNC20167F | C534 | 0.84 |
| BPNC20167F | C543 | 0.77 |
| BPNC20167F | C581 | 0.83 |
| BPNC20168F | C029 | 0.81 |
| BPNC20168F | C045 | 0.81 |
| BPNC20168F | C114 | 0.77 |
| BPNC20168F | C136 | 0.79 |
| BPNC20168R | C084 | 0.79 |
| BPNC20168R | C222 | 0.78 |
| BPNC20169F | C029 | 0.79 |
| BPNC20169F | C136 | 0.76 |
| BPNC20169F | C415 | 0.76 |
| BPNC20169R | C185 | 0.75 |
| BPNC20169R | C422 | 0.78 |
| BPNC20169R | C543 | 0.79 |
| BPNC20170R | C020 | 0.83 |
| BPNC20171R | C319 | -0.61 |
| BPNC20173R | C020 | 0.89 |
| BPNC20173R | C050 | 0.80 |
| BPNC20173R | C052 | 0.83 |
| BPNC20173R | C168 | 0.82 |
| BPNC20174F | C063 | 0.76 |
| BPNC20174F | C209 | 0.78 |
| BPNC20174F | C263 | 0.79 |
| BPNC20175F | C521 | 0.84 |
| BPNC20176F | C521 | 0.79 |
| BPNC20177F | C319 | -0.61 |
| BPNC20177R | C437 | 0.75 |
| BPNC20178F | C020 | 0.87 |
| BPNC20178F | C050 | 0.78 |
| BPNC20178F | C052 | 0.83 |
| BPNC20178F | C168 | 0.87 |
| BPNC20180F | C344 | 0.78 |
| BPNC20180R | C027 | 0.86 |
| BPNC20180R | C201 | 0.81 |
| BPNC20180R | C416 | 0.86 |
| BPNC20180R | C449 | 0.87 |
| BPNC20180R | C461 | 0.89 |
| BPNC20181R | C465 | 0.78 |
| BPNC20181R | C557 | 0.76 |
| BPNC20182F | C344 | 0.75 |
| BPNC20186F | C389 | 0.78 |
| BPNC20187F | C069 | 0.87 |
| BPNC20187F | C113 | 0.83 |
| BPNC20187F | C539 | 0.78 |
| BPNC20187F | C575 | 0.75 |
| BPNC20187F | C576 | 0.76 |
| BPNC20188F | C027 | 0.87 |
| BPNC20188F | C201 | 0.81 |
| BPNC20188F | C416 | 0.88 |
| BPNC20188F | C449 | 0.89 |
| BPNC20188F | C461 | 0.90 |
