# Supplementary material for: The Condition-Dependent Transcriptional Landscape of Burkholderia pseudomallei
Source: PLoS Genet. 2013 Sep 12;9(9):e1003795. doi: 10.1371/journal.pgen.1003795 (PMC3772027; doi:10.1371/journal.pgen.1003795)
Supplement: Table S10 — Condition subgroups identified by condition clustering. (DOC) [file pgen.1003795.s018.doc]

Table S10. Condition subgroups identified by condition clustering. The full experimental descriptions for each sample are reported in Table S1.

| **Group Color** | **Group ID** | **Samples** |
| --- | --- | --- |
|  | 1 | K9UV10mins  K9UV10minsref  K9UV1hrref  K928C30mins  K9UV1hr  K942C30mins  K937C30mins  K904C16hrs  K904C30mins  K92MNaCl  Bp22LBML  Bp22LB3Hr  Bp22LB4Hr |
|  | 2 | K9Chlamp  K9Ceft  K942C16hrs  K92MSorb |
|  | 3 | K937C16hrs  K9OsmRef  K9MHB  K9Bleach  K9ChemRef  K928C16hrs |
|  | 4 | K9Revived  K9DesRef  K9Des24hrs |
|  | 5 | K91XTSBDC24hrs  K9Al3+24hrs  K9Pb2+24hrs  K9Ni2+24hrs |
|  | 6 | K9Plate  K9H2O24hrref  K930NHS  K9Fe3+24hrs |
|  | 7 | K9TaurineES  K9Na2SO4ES  K9TaurineML  K9Na2SO4ML |
|  | 8 | K9CDMML  K9CDMES |
|  | 9 | Bp22CDMES  Bp22CDMML |
|  | 10 | K9Mn2.ML  K9Ca2.ML  K91XTSBDCML  K9Fe3+ML  K9Cu2+ML  K9Mg2+ML  K9Zn2+ML |
|  | 11 | K9LBS  K9LBES  K9LBML |
|  | 12 | K91XDPBS16hrs  K9Insulin |
