# Supplementary material for: The Condition-Dependent Transcriptional Landscape of Burkholderia pseudomallei
Source: PLoS Genet. 2013 Sep 12;9(9):e1003795. doi: 10.1371/journal.pgen.1003795 (PMC3772027; doi:10.1371/journal.pgen.1003795)
Supplement: Table S11 — cis-regulatory motifs detected using genic upstream sequences from clusters and MRCNs. (DOC) [file pgen.1003795.s019.doc]

Table S11. Cis-regulated motifs detected using genic upstream sequences from clusters and MRCNs. Each motif sequence consists of stacks of symbols (nucleic acids), one stack for each position in the sequence. Conservation among the upstream motifs is indicated by the overall height of the stack. The relative frequency of each nucleic acid at one position is indicated by the height of symbols.

1. Motifs in Clusters. Three or less motifs were over-represented (E-value < 0.1) in the condition-dependent clusters. The asterisk (*) indicates that the particular motif is also similarly detected by using BioProspector.

| No. | Cluster | Motif 1 | Motif 2 | Motif 3 |
| --- | --- | --- | --- | --- |
| **1** | **C001** | 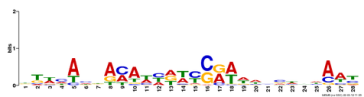 E=1.2e-23 | 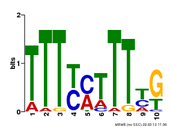 E=0.058 |  |
| **2** | **C002** | 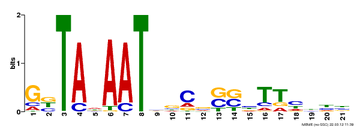3.4e-21* | 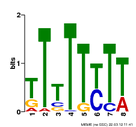 1.1e-08 | 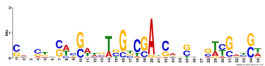 1e-07 |
| **3** | **C003** | 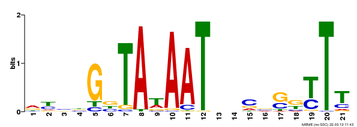 3.7e-101* | 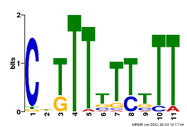 2.9e-15 |  |
| **4** | **C004** | 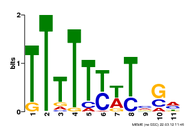 1.3e-14 | 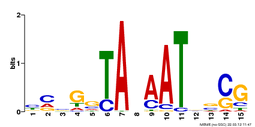 1.3e-10* |  |
| **5** | **C005** | 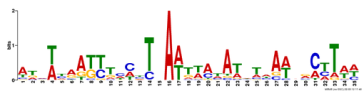 2.2e-15 | 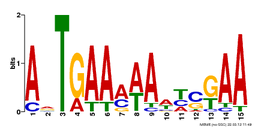 7e-08 |  |
| **6** | **C006** | 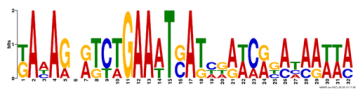 0.047 |  |  |
| **7** | **C007** | 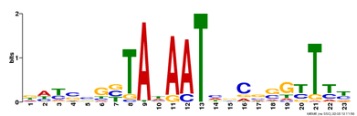 6.4e-50* | 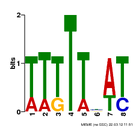 0.00038 |  |
| **8** | **C008** | 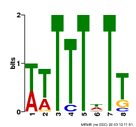 1.7e-06 | 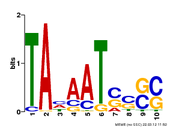 3.6e-06* |  |
| **9** | **C010** | 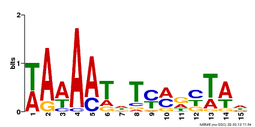 4.8e-07 | 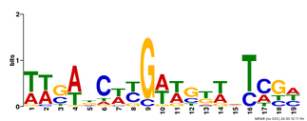 0.00015 |  |
| **10** | **C011** | 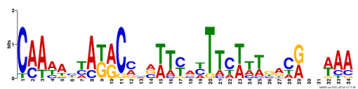 9.9e-10 |  |  |
| **11** | **C013** | 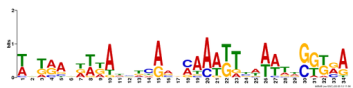 1.8e-06 |  |  |
| **12** | **C014** | 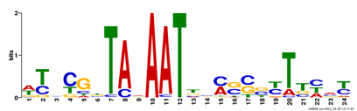 3.1e-15* | 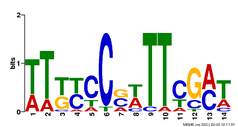 0.024 |  |
| **13** | **C015** | 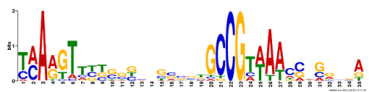 3.7e-30* | 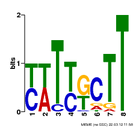 7.5e-05 |  |
| **14** | **C017** | 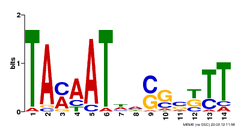 7.3e-10 |  |  |
| **15** | **C018** | 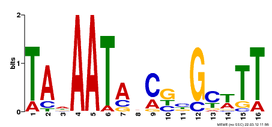 4e-06* | 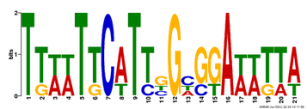 0.076 |  |
| **16** | **C019** | 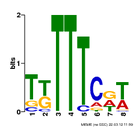 2e-04 | 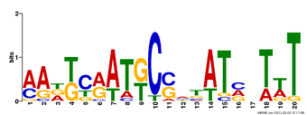 1e-04 |  |
| **17** | **C021** | 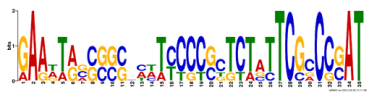 0.0026* |  |  |
| **18** | **C022** | 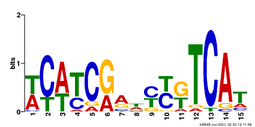 1.2e-14 | 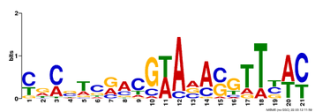 0.046 |  |
| **19** | **C024** | 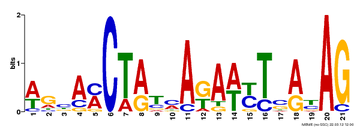1.7e-07* | 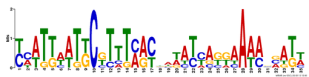 3.8e-07 | 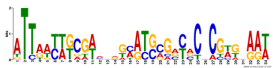 0.025 |
| **20** | **C025** | 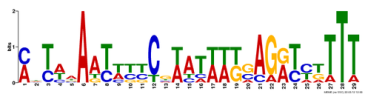 0.0016 |  |  |
| **21** | **C026** | 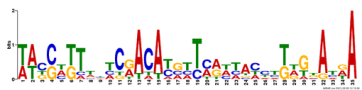 8.8e-05 |  |  |
| **22** | **C027** | 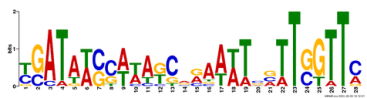 5.4e-27 |  |  |
| **23** | **C028** | 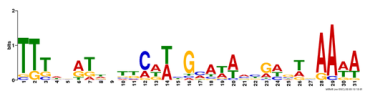 1.8e-08 | 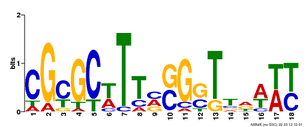 0.008 |  |
| **24** | **C029** | 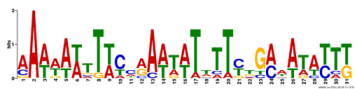 2.2e-28 | 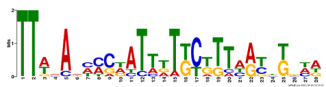 3.5e-10 | 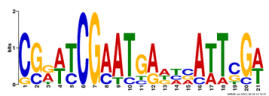 1.3e-05 |
| **25** | **C030** | 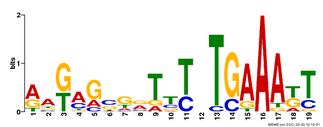 1.8e-07 |  |  |
| **26** | **C031** | 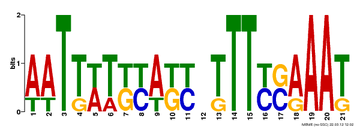 0.041 |  |  |
| **27** | **C032** | 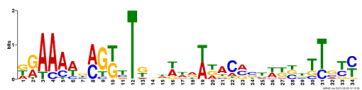 3.8e-06 | 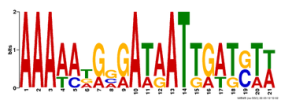 0.011 | 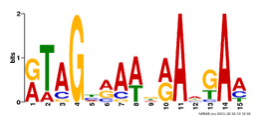 0.033 |
| **28** | **C033** | 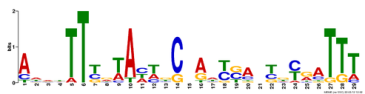 3e-05 | 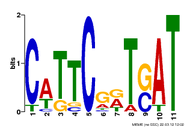 0.039 |  |
| **29** | **C035** | 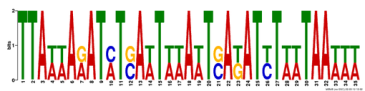 0.0013* |  |  |
| **30** | **C038** | 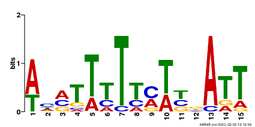 8.5e-05 | 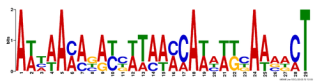 0.0034 |  |
| **31** | **C039** | 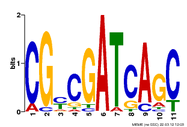 0.0031 | 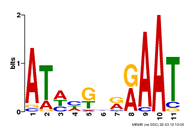 0.013 |  |
| **32** | **C040** | 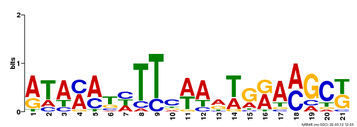 0.001 | 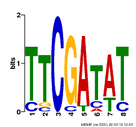 0.011 |  |
| **33** | **C041** | 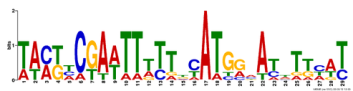 3.7e-12 | 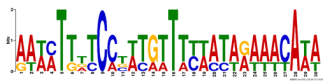 2.2e-07 |  |
| **34** | **C042** | 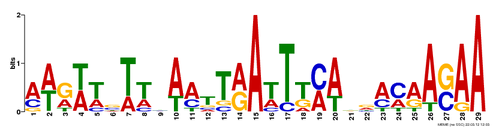 2.2e-09 | 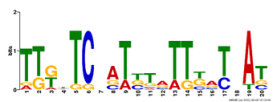 5.1e-06 |  |
| **35** | **C043** | 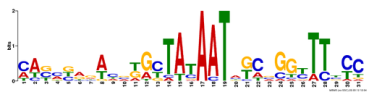 1e-21* |  |  |
| **36** | **C044** | 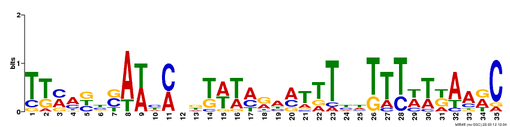 1.4e-07 |  |  |
| **37** | **C045** | 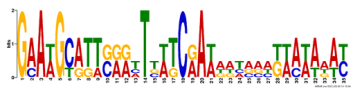 0.00061 |  |  |
| **38** | **C046** | 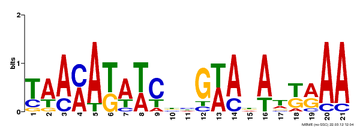0.0028 | 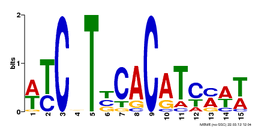 0.00062* | 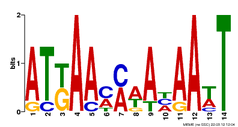 0.046 |
| **39** | **C047** | 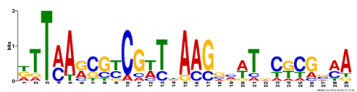 0.00027* |  |  |
| **40** | **C048** | 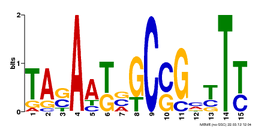 0.0031 |  | 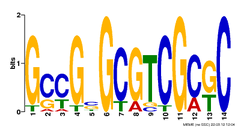 0.074 |
| **41** | **C049** | 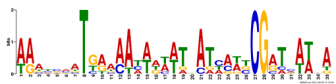 1.2e-08 |  |  |
| **42** | **C051** | 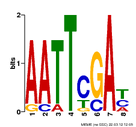 0.012 |  |  |
| **43** | **C053** | 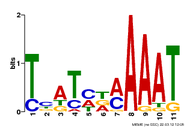 7.6e-06* | 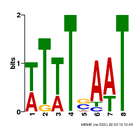 0.0033 | 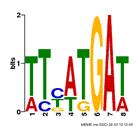 0.0014 |
| **44** | **C055** | 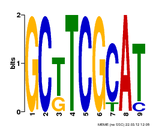 0.046* |  |  |
| **45** | **C056** | 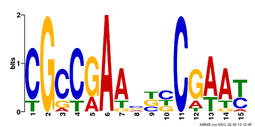 0.078 |  |  |
| **46** | **C057** | 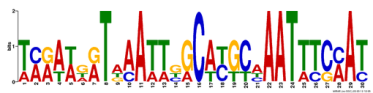 1.5e-06 |  |  |
| **47** | **C058** | 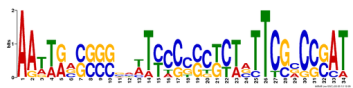 4.5e-09* |  |  |
| **48** | **C059** | 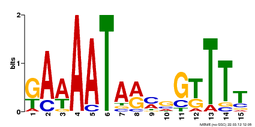 3.8e-06 |  |  |
| **49** | **C060** | 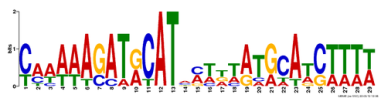 2.4e-23 |  |  |
| **50** | **C061** | 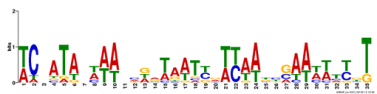 9.5e-17 |  | 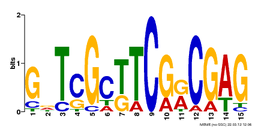 0.022 |
| **51** | **C062** | 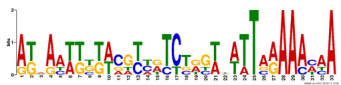 1.6e-07 | 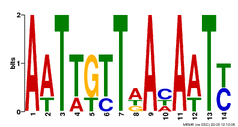 0.0039 |  |
| **52** | **C066** | 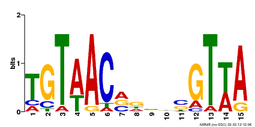 1.6e-07* | 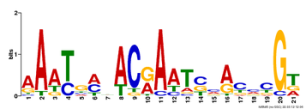 3.7e-06 |  |
| **53** | **C069** | 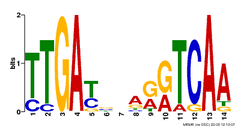 0.00018 |  |  |
| **54** | **C070** | 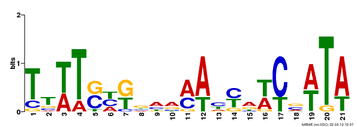3.5e-06 | 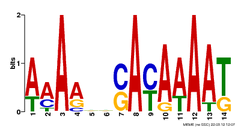 0.019 | 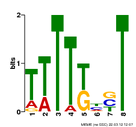 0.069 |
| **55** | **C073** | 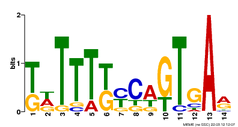 2.9e-07* |  |  |
| **56** | **C076** | 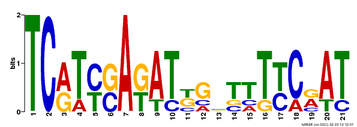 0.027 |  |  |
| **57** | **C077** | 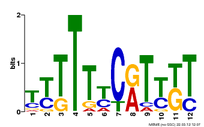 1.7e-06 |  |  |
| **58** | **C079** | 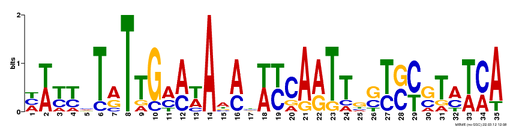3.7e-06 |  |  |
| **59** | **C080** | 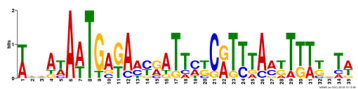 1.7e-11* |  |  |
| **60** | **C081** | 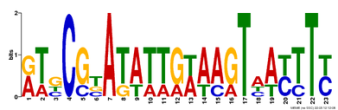 0.098* |  |  |
| **61** | **C082** | 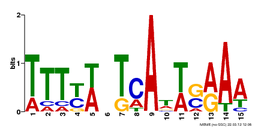 1.8e-05 |  |  |
| **62** | **C083** | 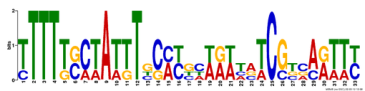 0.0065 |  |  |
| **63** | **C086** | 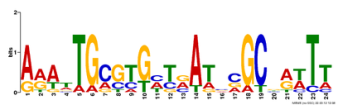0.026 |  |  |
| **64** | **C088** | 0.085 |  |  |
| **65** | **C091** | 1.4e-08 | 0.0025 |  |
| **66** | **C092** | 0.00039 |  |  |
| **67** | **C093** | 0.001 |  |  |
| **68** | **C095** | 8e-05 |  |  |
| **69** | **C097** | 0.0014 |  |  |
| **70** | **C098** | 1.7e-06* |  |  |
| **71** | **C099** | 0.00074 |  |  |
| **72** | **C103** | 0.099 |  |  |
| **73** | **C106** | 0.00013 | 0.013 |  |
| **74** | **C107** | 0.00014* | 0.0014 |  |
| **75** | **C109** | 0.0022* |  |  |
| **76** | **C110** | 0.003 |  |  |
| **77** | **C111** | 4.6e-05 | 0.03 |  |
| **78** | **C112** | 1.2e-09* |  |  |
| **79** | **C119** | 2.9e-05* | 0.013 |  |
| **80** | **C120** | 0.07 |  | 0.089 |
| **81** | **C121** | 0.0028 |  |  |
| **82** | **C122** | 0.00018* |  |  |
| **83** | **C124** | 0.011 |  |  |
| **84** | **C126** | 0.00023 |  |  |
| **85** | **C128** | 0.00044 | 0.0038 | 0.035 |
| **86** | **C132** | 8.1e-09* |  |  |
| **87** | **C134** | 3.4e-07 | 0.092 |  |
| **88** | **C140** | 0.013 |  |  |
| **89** | **C142** | 0.0039 |  |  |
| **90** | **C143** | 0.001 |  |  |
| **91** | **C153** | 2.3e-07* |  |  |
| **92** | **C155** | 4.4e-11 | 6.5e-09 | 0.0052 |
| **93** | **C160** | 1.3e-07* | 0.015 |  |
| **94** | **C162** | 1.1e-09* |  |  |
| **95** | **C163** | 0.00065 |  |  |
| **96** | **C164** | 4.4e-15* |  |  |
| **97** | **C179** | 0.00056 |  |  |
| **98** | **C184** | 0.072 |  |  |
| **99** | **C187** | 0.028 |  |  |
| **100** | **C188** | 0.057 |  |  |
| **101** | **C190** | 0.00079 |  |  |
| **102** | **C192** | 0.0017 | 0.064 |  |
| **103** | **C198** | 0.003* | 0.021 |  |
| **104** | **C199** | 0.017 |  |  |
| **105** | **C201** | 4.7e-05* |  |  |
| **106** | **C216** | 0.0083* |  |  |
| **107** | **C221** | 0.0056 |  |  |
| **108** | **C228** | 0.00083 |  |  |
| **109** | **C229** | 6.5e-08* | 7.2e-06 |  |
| **110** | **C230** | 0.032 * |  |  |
| **111** | **C233** | 0.015 |  |  |
| **112** | **C237** | 0.063 |  |  |
| **113** | **C240** | 0.021 |  |  |
| **114** | **C249** | 0.013 |  |  |
| **115** | **C258** | 0.0014 |  |  |
| **116** | **C275** | 0.09 |  |  |
| **117** | **C282** | 0.0094* |  |  |
| **118** | **C290** | 0.024 |  |  |
| **119** | **C297** | 0.016 |  |  |
| **120** | **C328** | 0.0056 |  |  |
| **121** | **C329** | 0.00056 |  |  |
| **122** | **C349** | 0.00018* |  |  |
| **123** | **C354** | 0.0047 |  |  |
| **124** | **C355** | 0.0098 |  |  |
| **125** | **C366** | 0.062 |  |  |
| **126** | **C382** | 0.00048* |  |  |
| **127** | **C389** | 0.068* |  |  |
| **128** | **C398** | 0.024 |  |  |
| **129** | **C400** | 0.00014* |  |  |
| **130** | **C410** | 3.6e-05* | 0.001 | 0.018 |
| **131** | **C434** | 1.2e-18* | 6.8e-17 | 2.8e-07 |
| **132** | **C485** | 0.02* |  |  |
| **133** | **C493** | 0.034 |  |  |
| **134** | **C501** | 0.081 |  |  |
| **135** | **C602** | 0.0054 |  |  |
| **136** | **C611** | 0.00082* |  |  |
| **137** | **C621** | 0.00015 | 0.044 |  |

1. Motifs in MRCNs. One or more motifs that were over-represented (E-value < 0.1) in the maximum-relatedness of cluster networks (MRCNs). The asterisk (*) indicates that the particular motif is also similarly detected by using BioProspector.

| **No** | **MRCN** | Motif 1 | Motif 2 | Motif 3 |
| --- | --- | --- | --- | --- |
| **1** | **C001-C006-C076-C220** | E=4.9e-33 | E=1.1e-09 | E=9.8e-07 |
| **2** | **C020-C052-C168** | 4.7e-17* | 1.7e-08 |  |
| **3** | **C021-C058** | 0.00026* |  |  |
| **4** | **C024-C096-C336** | 2e-10 | 1.3e-09 |  |
| **5** | **C027-C201-C416-C449-C461-C361** | 6.2e-51 |  |  |
| **6** | **C029-C393** | 1.1e-27* | 1.8e-09 | 4.4e-05 |
| **7** | **C030-C035** | 1.5e-10 |  |  |
| **8** | **C033-C087-C447** | 1.9e-13 |  |  |
| **9** | **C036-C135-C608** | 0.036 |  |  |
| **10** | **C041-C236** | 1.2e-08 | 4.4e-07 | 0.0022 |
| **11** | **C042-C045-C136-C114** | 6.3e-23 | 3.6e-09 | 1.9e-07 |
| **12** | **C046-C097-C240** | 2e-14 | 2.7e-07 | 5.6e-06 |
| **13** | **C053-C195-C226-C111** | 1.5e-15 | 0.0064 | 0.032 |
| **14** | **C054-C139-C571-C619** | 4.9e-06 |  |  |
| **15** | **C055-C210-C322** | 1.7e-06* |  |  |
| **16** | **C059-C602** | 5.5e-08 | 0.0019 |  |
| **17** | **C062-C306-C414** | 1.6e-06 |  |  |
| **18** | **C066-C122-C417-C434-C455** | 7.6e-34* | 7.5e-18 | 1.1e-06 |
| **19** | **C069-C113-C539-C437-C253-C216-C462** | 7e-17* | 0.00057 |  |
| **20** | **C071-C418** | 0.0012 | 0.044 |  |
| **21** | **C080-C386-C400-C446-C448** | 1.9e-34* | 8.1e-07 |  |
| **22** | **C091-C368-C485** | 6.8e-22 | 3.1e-11 | 1.6e-05 |
| **23** | **C092-C112** | 9.4e-20* |  |  |
| **24** | **C094-C313-C165** | 1.1e-06 |  |  |
| **25** | **C095-C128** | 1.9e-10 | 3.7e-06 | 1e-04 |
| **26** | **C107-C482** | 0.018 | 0.017 |  |
| **27** | **C110-C510** | 3.6e-08* |  |  |
| **28** | **C126-C524-C198** | 2.7e-09 | 0.0083 |  |
| **29** | **C131-C265** | 0.013 |  |  |
| **30** | **C132-C389-C596** | 1.6e-27* |  |  |
| **31** | **C133-C174-C440** | 5.7e-05 | 0.01 |  |
| **32** | **C138-C358-C403-C573** | 0.0068 |  |  |
| **33** | **C147-C516** | 0.05 |  |  |
| **34** | **C153-C617** | 1.2e-07* |  |  |
| **35** | **C162-C340** | 1.5e-10 |  |  |
| **36** | **C164-C391-C392** | 4e-18* |  |  |
| **37** | **C167-C249** | 2e-04 |  |  |
| **38** | **C169-C186** | 0.00087* |  |  |
| **39** | **C172-C205-C263-C562-C473-C402** | 9.3e-05 | 0.0089 |  |
| **40** | **C179-C339** | 1.1e-06* |  |  |
| **41** | **C219-C270** | 0.036 |  |  |
| **42** | **C227-C307-C374-C376-C292-C569** | 4.8e-07 | 0.00015 | 9.5e-05 |
| **43** | **C229-C315** | 1.8e-05 | 3.8e-06* | 0.00024 |
| **44** | **C232-C299-C408** | 2.4e-05 |  |  |
| **45** | **C243-C385** | 0.0011 |  |  |
| **46** | **C273-C483** | 0.027 |  |  |
| **47** | **C304-C428-C553-C565** | 2.5e-07 |  |  |
| **48** | **C324-C334** | 0.026 |  |  |
| **49** | **C326-C360** | 0.0043* |  |  |
| **50** | **C329-C545** | 1.3e-06 | 2e-06* | 2e-04 |
| **51** | **C398-C611** | 1.4e-08 |  |  |
| **52** | **C511-C568** | 0.04 |  |  |
