# Supplementary material for: The Condition-Dependent Transcriptional Landscape of Burkholderia pseudomallei
Source: PLoS Genet. 2013 Sep 12;9(9):e1003795. doi: 10.1371/journal.pgen.1003795 (PMC3772027; doi:10.1371/journal.pgen.1003795)
Supplement: Table S12 — ncRNAs with identified cis-regulatory motifs. (DOC) [file pgen.1003795.s020.doc]

Table S12. ncRNAs with identified *cis*-regulatory motifs. Each motif sequence consists of stacks of symbols (nucleic acids), one stack for each position in the sequence. Conservation among the upstream motifs is indicated by the overall height of the stack. The relative frequency of each nucleic acid at one position is indicated by the height of symbols.

|  | **Cluster/Motif** | **ncRNA/motif sequence** | **Location of ncRNA’s motif** | | | |
| --- | --- | --- | --- | --- | --- | --- |
|  |  |  | Chr | S | Start | Stop |
| 1 | C080  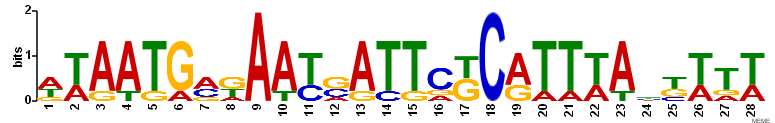 | BPNC20041F  TAAATGCAAATGATTCTCATTTATCAAT | 2 | + | 673187 | 673214 |
| 2 | C055, C210, C322  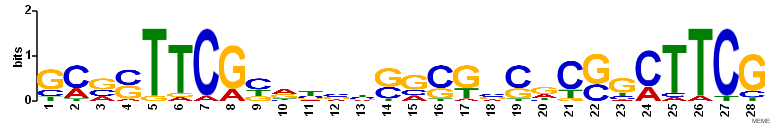 | BPNC20065R  CACGTTCGTGTTTCGCGACGCGGCTTCC | 2 | - | 1077394 | 1077367 |
| 3 | C120  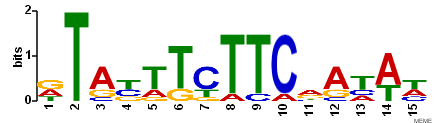 | BPNC10012R  CTGTTACAGCTTCAAAAATAT | 1 | - | 130546 | 130532 |
| 4 | C015  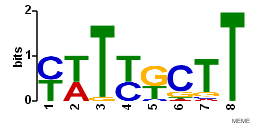 | BPNC10016F  CTTCGCTT | 1 | + | 294760 | 294767 |
| 5 | C021，C058  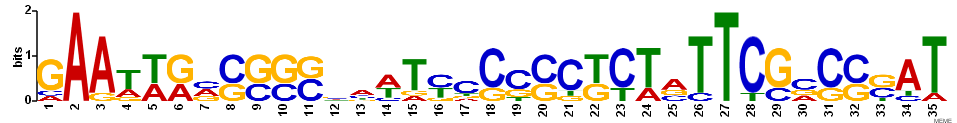 | BPNC10024R  GGCGAATAGCCGGCAAAGGGCCCCTCTATTCGCGGTTTGCC | 1 | - | 282436 | 282402 |

|  | **Cluster/Motif** | **ncRNAs/motif sequence** | **Location of ncRNA’s motif** | | | |
| --- | --- | --- | --- | --- | --- | --- |
|  |  |  | Chr | S | Start | Stop |
| 6 | C020，C052，C168  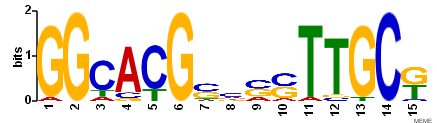 | BPNC10045F  GTGGGCGCGGCGCTTGCTGTT | 1 | + | 834034 | 834048 |
| 7 | C160  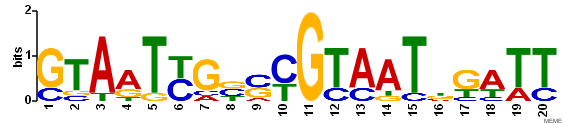 | BPNC10045R  CGGCGAATCGCGCGCCGTCGATT CGA | 1 | - | 664509 | 664490 |
| 8 | C053  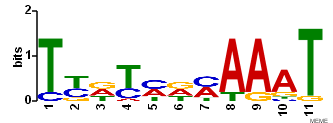 | BPNC10047F   | TTGTCGTTACT |  | | --- | --- | | 1 | + | 866928 | 866938 |
| 9 | C007  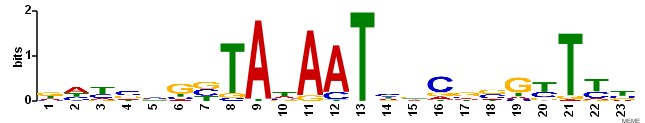 | BPNC10053F  GCTGCGGTATAATTTCGAGGTTT | 1 | + | 1010291 | 1010313 |
| 10 | C053  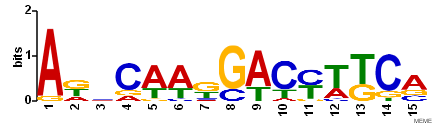 | BPNC10056F  ATACTATGTCTTTCA | 1 | + | 1092469 | 1092483 |

|  | **Cluster/Motif** | **ncRNAs/motif sequence** | **Location of ncRNA’s motif** | | | |
| --- | --- | --- | --- | --- | --- | --- |
|  |  |  | Chr | S | Start | Stop |
| 11 | C014  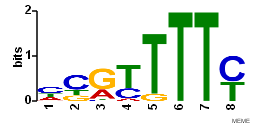 | BPNC10056R  CGGTTTTT | 1 | - | 998934 | 998927 |
| 12 | C007  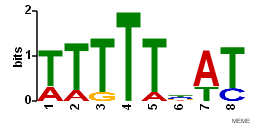 | BPNC10057R  TTTTTGAT | 1 | - | 1012005 | 1011998 |
| 13 | C007  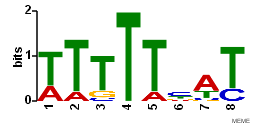 | BPNC10062R  ATTTTCCT | 1 | - | 1069841 | 1069834 |
| 14 | C053  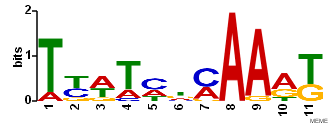 | BPNC10063R  ACAAATAAATT | 1 | - | 1091043 | 1091033 |
| 15 | C229  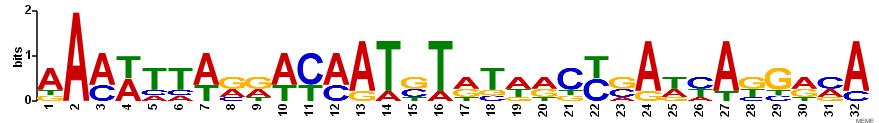 | BPNC10064R  AAAAATTCGTTAGTTTTCAAGTGATTAGCGGA | 1 | - | 1112289 | 1112258 |

|  | **Cluster/Motif** | **ncRNAs/motif sequence** | **Location of ncRNA’s motif** | | | |
| --- | --- | --- | --- | --- | --- | --- |
|  |  |  | Chr | S | Start | Stop |
| 16 | C119  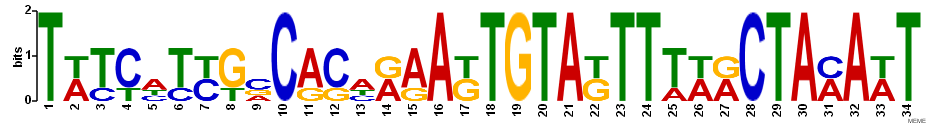 | BPNC10072R  TTTCATCGCCACAAAAGTGTATTTTTGCTAAATT | 1 | - | 1199799 | 1199766 |
| 17 | C066  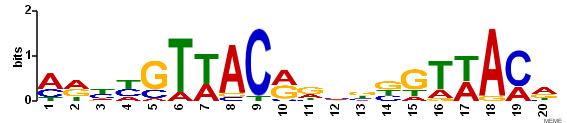 | BPNC10076F  AACTGTTACGAACGTTTACG | 1 | + | 1370721 | 1370740 |
| 18 | C066  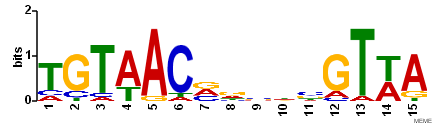 | BPNC10076R  TGCAACGAGTTGTAT | 1 | - | 1221504 | 1221490 |
| 19 | C109  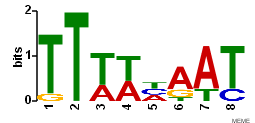 | BPNC10078R  TTAAAAAT | 1 | - | 1226247 | 1226240 |
| 20 | C128  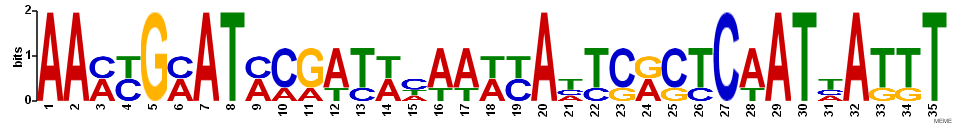 | BPNC10099R  AACCGAATACGATTCAAACATTCACTCAATTATTT | 1 | - | 1620027 | 1619993 |

|  | **Cluster/Motif** | **ncRNAs/motif sequence** | **Location of ncRNA’s motif** | | | |
| --- | --- | --- | --- | --- | --- | --- |
|  |  |  | Chr | S | Start | Stop |
| 21 | C007  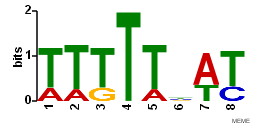 | BPNC10125F  TTTTTTTC | 1 | + | 2216388 | 2216395 |
| 22 | C007  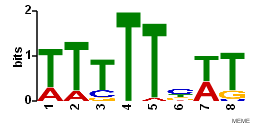 | BPNC10127F  TTTTTCTG | 1 | + | 2304494 | 2304501 |
| 23 | C005  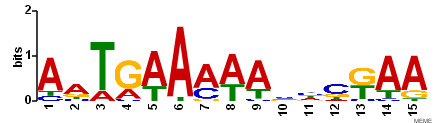 | BPNC10132F  AAAGTAAAACACGTG | 1 | + | 2330799 | 2330813 |
| 24 | C015  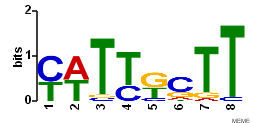 | BPNC10132R  CATTCGTC | 1 | - | 2352532 | 2352525 |
| 25 | C382  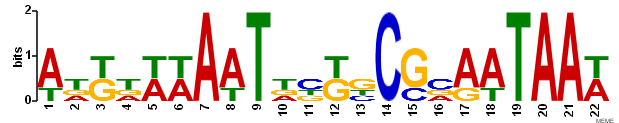 | BPNC10139R  ATTTAAAATAGTGCGCGATAAA | 1 | - | 2454540 | 2454519 |

|  | **Cluster/Motif** | **ncRNAs/motif sequence** | **Location of ncRNA’s motif** | | | |
| --- | --- | --- | --- | --- | --- | --- |
|  |  |  | Chr | S | Start | Stop |
| 26 | C003  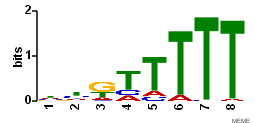 | BPNC10146R  TCGTTTTT | 1 | - | 2590712 | 2590705 |
| 27 | C007  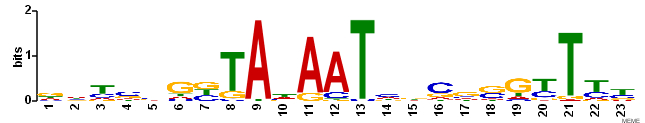 | BPNC10162F  GGTATGGGAAAATGCGGGGTTTC | 1 | + | 3052430 | 3052452 |
| 28 | C119  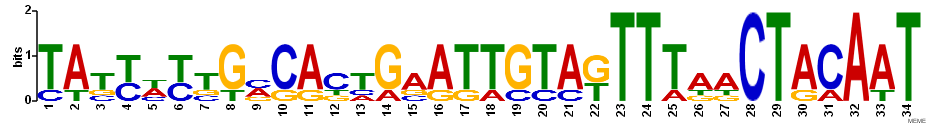 | BPNC10176R  TATTTTTGACACTGAATTGTAGTTTAACTACAAT | 1 | - | 3133541 | 3133508 |
| 29 | C073  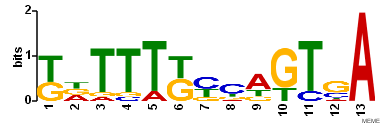 | BPNC10178F  GGATTGCAAGCCA | 1 | + | 3399117 | 3399129 |
| 30 | C073  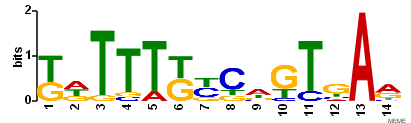 | BPNC10190R  GATTTGTCCCCTAA | 1 | - | 3298478 | 3298465 |

|  | **Cluster/Motif** | **ncRNAs/motif sequence** | **Location of ncRNA’s motif** | | | |
| --- | --- | --- | --- | --- | --- | --- |
|  |  |  | Chr | S | Start | Stop |
| 31 | C054, C139, C571, C619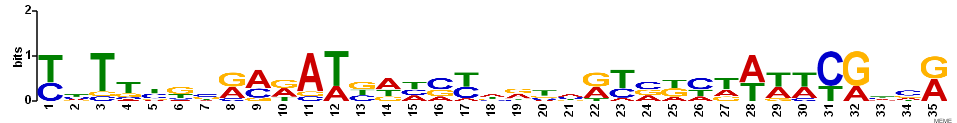 | BPNC10193F  TTGGGGGAAAGATTTCAAATATCGTCAAAACGTAG | 1 | + | 3689353 | 3689387 |
| 32 | C382  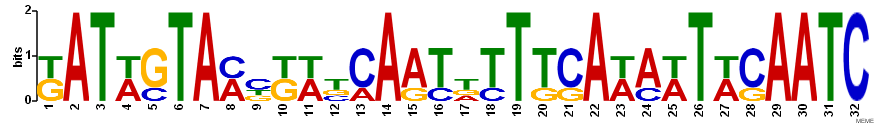 | BPNC10194F  TATAGTACCGATCAATTTTTCATAATTCAATC | 1 | + | 3714023 | 3714054 |
| 33 | C003  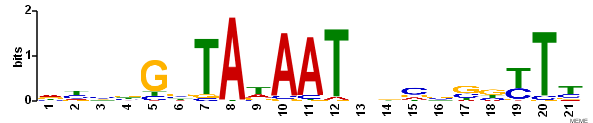 | BPNC10209R  GCCGGTTATCAAAGACCGTTG | 1 | - | 3826072 | 3826052 |
| 34 | C083  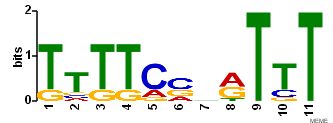 | BPNC10210R  TTTTCCGGTGT | 1 | - | 3830329 | 3830319 |
| 35 | C126  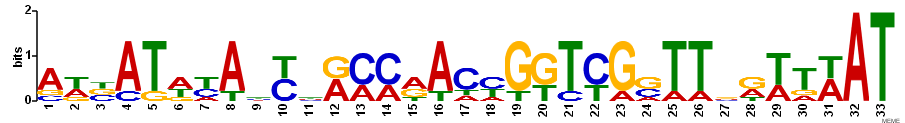 | BPNC10213R  ATTATAAACCAACCGACCGGTCGGTTAATATAT | 1 | - | 3838578 | 3838546 |

|  | **Cluster/Motif** | **ncRNAs/motif sequence** | **Location of ncRNA’s motif** | | | |
| --- | --- | --- | --- | --- | --- | --- |
|  |  |  | Chr | S | Start | Stop |
| 36 | C229  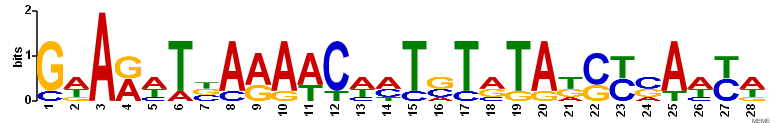 | BPNC20010R  CAAGCTGAAATTATTGCGTATGCGACTA | 2 | - | 84741 | 84714 |
| 37 | C020, C052, C168  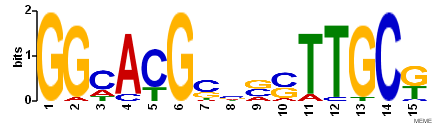 | BPNC20014F  GGAACGCGGCTTGCG | 2 | + | 350840 | 350854 |
| 38 | C132,C389,C596  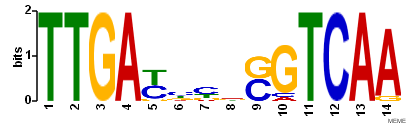 | BPNC20018F  TTGATTCCGATCAG | 2 | + | 367296 | 367309 |
| 39 | C042,C045,C136,C114  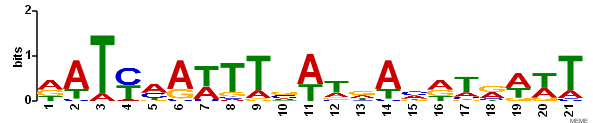 | BPNC20028F  AATCAGTCAGTTAAGTCGAGT | 2 | + | 491515 | 491535 |

|  | **Cluster/Motif** | **ncRNAs/motif sequence** | **Location of ncRNA’s motif** | | | |
| --- | --- | --- | --- | --- | --- | --- |
|  |  |  | Chr | S | Start | Stop |
| 40 | C621  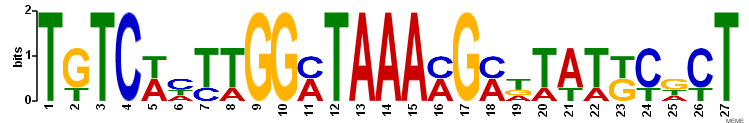 | BPNC20056F  TGTCACTTGGCTAAACGCTTATGCGCT | 2 | + | 894669 | 894695 |
| 41 | C621  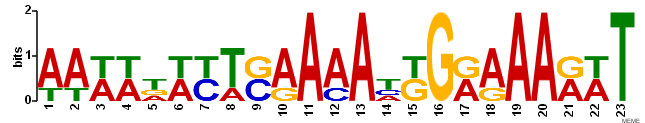 | BPNC20057F  AATTGACAGGAAAATGAAAAGTT | 2 | + | 897440 | 897462 |
| 42 | C621  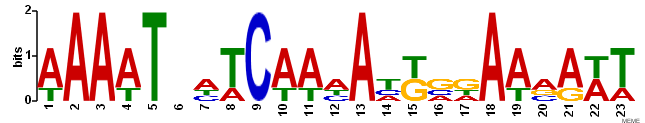 | BPNC20061F  TAAATGATCTTTATGCTATGGTA | 2 | + | 899671 | 899693 |
| 43 | C029,C393  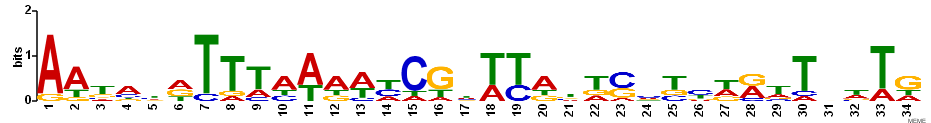 | BPNC20066F  AGGCCTTTTTTTATCGTTCAATGCCATGAATTTA | 2 | + | 918215 | 918248 |
| 44 | C024,C096,C336  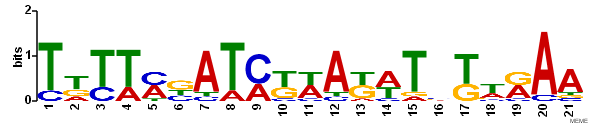 | BPNC20104F  TTTTATATCGAATATCGTGAA | 2 | + | 1587217 | 1587237 |

|  | **Cluster/Motif** | **ncRNAs/motif sequence** | **Location of ncRNA’s motif** | | | |
| --- | --- | --- | --- | --- | --- | --- |
|  |  |  | Chr | S | Start | Stop |
| 45 | C018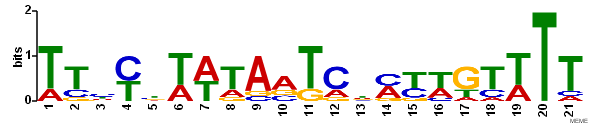 | BPNC20126R  TCCCGTATAATCCGCAGCTTT | 2 | - | 2154669 | 2154649 |
| 46 | C029  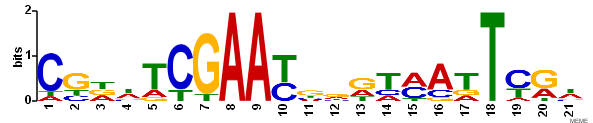 | BPNC20132R  CGGATCGAATCGGTAAATTGT | 2 | - | 2312576 | 2312556 |
| 47 | C069  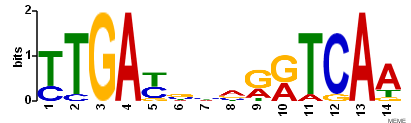 | BPNC20151R  CTGACGCCGATCAT | 2 | - | 2630268 | 2630255 |
| 48 | C110,C510  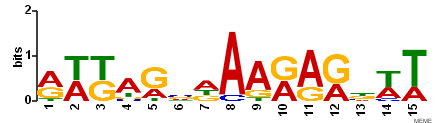 | BPNC20155R  TTGGACGATGAGCAT | 2 | - | 2770428 | 2770414 |
| 49 | C069  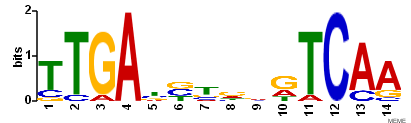 | BPNC20156F  TTGACCTGCGTCAA | 2 | + | 2600998 | 2601011 |
|  | **Cluster/Motif** | **ncRNAs/motif sequence** | **Location of ncRNA’s motif** | | | |
|  |  |  | Chr | S | Start | Stop |
| 50 | C005  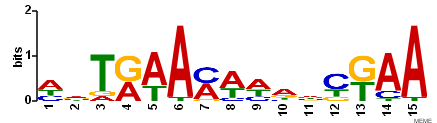 | BPNC20157R  TTGGAAACTGACTAA | 2 | - | 2789764 | 2789750 |
| 51 | C042,C045,C136,C114  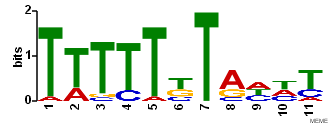 | BPNC20168F  TTTTTCTACCT | 2 | + | 2872252 | 2872262 |
| 52 | C389  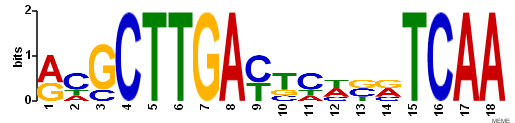 | BPNC20186F  GTCCTTGACTACGATCAA | 2 | + | 3097306 | 3097323 |
| 53 | C069  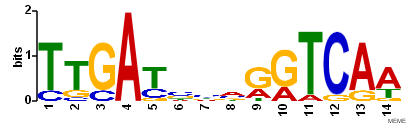 | BPNC20187F  TGCATCCCGGTCGT | 2 | + | 3098888 | 3098901 |
| 54 | C027,C201,C416,C449,C461,C361  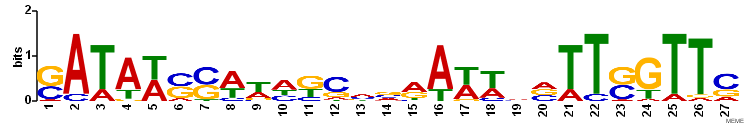 | BPNC20188F  GCTTTCGAATCGCAGTTTTCATCGATC | 2 | + | 3105805 | 3105831 |
