# Supplementary material for: The Condition-Dependent Transcriptional Landscape of Burkholderia pseudomallei
Source: PLoS Genet. 2013 Sep 12;9(9):e1003795. doi: 10.1371/journal.pgen.1003795 (PMC3772027; doi:10.1371/journal.pgen.1003795)
Supplement: Table S13 — Differential expression of quorum sensing signature genes. (DOC) [file pgen.1003795.s021.doc]

Table S13. List of genes showing altered expression by at least 2-fold in *pmlI* mutant (Bp008*ΔpmlI*) with respect to its wild type (Bp008).

| **Gene** | **(Up) Fold Change** |  | **Gene** | **(Down) Fold Change** |
| --- | --- | --- | --- | --- |
| BPSS1835 | 38.4 |  | BPSS0395 | -144.6 |
| BPSS1687 | 35.0 |  | BPSS0394 | -143.3 |
| BPSL0324 | 31.6 |  | BPSL1821 | -87.1 |
| BPSS2230 | 26.4 |  | BPSL3351 | -30.7 |
| BPSL1586 | 25.1 |  | BPSL3416 | -26.9 |
| BPSL1049 | 24.3 |  | BPSS0585 | -26.6 |
| BPSL3114 | 23.1 |  | BPSL0940 | -20.1 |
| BPSS0677 | 23.1 |  | BPSL3073 | -18.8 |
| BPSS1682 | 21.5 |  | BPSS1737 | -16.9 |
| BPSS1688 | 20.6 |  | BPSS0806 | -15.7 |
| BPSL1795 | 20.0 |  | BPSL1269 | -14.2 |
| BPSL1133 | 19.8 |  | BPSS1027 | -13.7 |
| BPSS1689 | 19.5 |  | BPSL0939 | -12.5 |
| BPSL2251 | 17.0 |  | BPSS0771 | -12.4 |
| BPSS1888 | 16.7 |  | BPSS0840 | -12.4 |
| BPSS2219 | 15.3 |  | BPSS2272 | -12.3 |
| BPSS2216 | 15.2 |  | BPSL1073 | -12.2 |
| BPSS1886 | 14.9 |  | BPSL0942 | -12.1 |
| BPSL1045 | 14.5 |  | BPSS0805 | -11.7 |
| BPSS1686 | 14.2 |  | BPSL3417 | -11.2 |
| BPSL1044 | 14.0 |  | BPSL1900 | -11.1 |
| BPSS1825 | 13.8 |  | BPSL3380 | -11.1 |
| BPSS1885 | 13.8 |  | BPSS0269 | -10.7 |
| BPSS1834 | 13.3 |  | BPSS1028 | -10.3 |
| BPSS1832 | 12.8 |  | BPSS1029 | -9.8 |
| BPSS1831 | 11.8 |  | BPSL1743 | -9.8 |
| BPSS1683 | 11.7 |  | BPSS0731 | -9.1 |
| BPSL0175 | 11.6 |  | BPSL1744 | -8.9 |
| BPSS1887 | 11.4 |  | BPSL3415 | -8.8 |
| BPSS1060 | 11.3 |  | BPSL1899 | -8.8 |
| BPSS1830 | 11.3 |  | BPSS2271 | -8.5 |
| BPSL0328 | 11.2 |  | BPSS1467 | -8.4 |
| BPSS1829 | 11.1 |  | BPSL0113A | -8.0 |
| BPSS1485 | 10.7 |  | BPSS1149 | -8.0 |
| BPSL1796 | 10.3 |  | BPSL3309 | -8.0 |
| BPSS1061 | 10.3 |  | BPSL1895 | -7.9 |
| BPSL1046 | 10.2 |  | BPSS2141 | -7.9 |
| BPSS2214 | 10.0 |  | BPSL3418 | -7.7 |
| BPSL0174 | 9.9 |  | BPSS1385 | -7.7 |
| BPSL3115 | 9.8 |  | BPSL1870 | -7.6 |
| BPSS1833 | 9.6 |  | BPSS1466 | -7.5 |
| BPSS1484 | 9.5 |  | BPSL2747 | -7.5 |
| BPSS1892 | 9.4 |  | BPSS0141 | -7.4 |
| BPSS2049 | 9.2 |  | BPSL3284 | -7.3 |
| BPSS2046 | 9.1 |  | BPSS2270 | -7.0 |
| BPSS1660 | 9.0 |  | BPSL1897 | -7.0 |
| BPSS1828 | 9.0 |  | BPSL3414 | -6.9 |
| BPSL3089 | 8.8 |  | BPSS2273 | -6.8 |
| BPSS1893 | 8.8 |  | BPSS1430 | -6.8 |
| BPSS1486 | 8.6 |  | BPSL1843 | -6.7 |
| BPSS1891 | 8.5 |  | BPSS1285 | -6.7 |
| BPSL1057 | 8.5 |  | BPSL1889 | -6.7 |
| BPSL1797 | 8.1 |  | BPSS1152 | -6.6 |
| BPSS1684 | 8.1 |  | BPSL1890 | -6.6 |
| BPSS1685 | 8.1 |  | BPSL0594 | -6.5 |
| BPSS1733 | 7.9 |  | BPSL0281 | -6.5 |
| BPSL0061 | 7.8 |  | BPSL2746 | -6.4 |
| BPSL1618 | 7.6 |  | BPSS0002 | -6.3 |
| BPSL1134 | 7.6 |  | BPSL3074 | -6.3 |
| BPSS1826 | 7.6 |  | BPSS1334 | -6.3 |
| BPSS2022 | 7.5 |  | BPSL1079 | -6.2 |
| BPSS2215 | 7.5 |  | BPSL1893 | -6.2 |
| BPSS2213 | 7.4 |  | BPSS0142 | -6.1 |
| BPSL1559 | 7.4 |  | BPSL0941 | -6.1 |
| BPSS0043 | 7.2 |  | BPSS0804 | -6.1 |
| BPSS1836 | 7.1 |  | BPSL3247 | -6.1 |
| BPSL3090 | 7.1 |  | BPSS1961 | -6.0 |
| BPSL1560 | 7.1 |  | BPSL0502 | -6.0 |
| BPSS1839 | 7.1 |  | BPSL1834 | -6.0 |
| BPSL1050 | 7.0 |  | BPSL2027 | -6.0 |
| BPSL1561 | 6.9 |  | BPSS1384a | -6.0 |
| BPSL0898 | 6.7 |  | BPSL0368 | -5.9 |
| BPSS0427 | 6.7 |  | BPSL0609 | -5.9 |
| BPSL1393 | 6.6 |  | BPSL0503 | -5.9 |
| BPSL3255 | 6.6 |  | BPSL0129 | -5.8 |
| BPSL1695 | 6.5 |  | BPSL3319 | -5.8 |
| BPSL1588 | 6.5 |  | BPSL2028 | -5.7 |
| BPSL1616 | 6.5 |  | BPSL2558 | -5.7 |
| BPSS2023 | 6.4 |  | BPSL0501 | -5.7 |
| BPSS1047 | 6.4 |  | BPSL2817 | -5.7 |
| BPSS0426 | 6.3 |  | BPSL2952 | -5.7 |
| BPSL0329 | 6.3 |  | BPSL2488 | -5.7 |
| BPSS0571 | 6.2 |  | BPSL1753 | -5.6 |
| BPSS1681 | 6.2 |  | BPSL2140 | -5.6 |
| BPSL1794 | 6.2 |  | BPSS2057 | -5.6 |
| BPSL0685 | 6.2 |  | BPSS1183 | -5.6 |
| BPSS0493 | 6.2 |  | BPSS0730 | -5.6 |
| BPSL0327 | 6.0 |  | BPSS0983 | -5.6 |
| BPSL1584 | 6.0 |  | BPSL1894 | -5.6 |
| BPSS0279 | 5.9 |  | BPSS1912 | -5.6 |
| BPSL1054 | 5.9 |  | BPSS0022 | -5.6 |
| BPSL2055 | 5.9 |  | BPSL0583 | -5.5 |
| BPSS0214 | 5.7 |  | BPSL1898 | -5.5 |
| BPSS0879 | 5.7 |  | BPSL3307 | -5.5 |
| BPSS0044 | 5.7 |  | BPSL2508 | -5.5 |
| BPSL1615 | 5.4 |  | BPSL1028 | -5.4 |
| BPSS1076 | 5.4 |  | BPSL0921 | -5.3 |
| BPSL1206 | 5.3 |  | BPSL2073 | -5.3 |
| BPSS1827 | 5.1 |  | BPSL3352 | -5.3 |
| BPSS0277 | 5.1 |  | BPSL1932 | -5.2 |
| BPSS2103 | 5.1 |  | BPSS2140 | -5.2 |
| BPSS1906 | 5.1 |  | BPSS1468 | -5.2 |
| BPSL3340 | 5.0 |  | BPSL2465 | -5.2 |
| BPSS1201 | 5.0 |  | BPSL0290 | -5.2 |
| BPSL3091 | 5.0 |  | BPSS1972 | -5.2 |
| BPSL1721 | 5.0 |  | BPSS1444 | -5.1 |
| BPSL1392 | 4.9 |  | BPSS0140 | -5.1 |
| BPSS1905 | 4.8 |  | BPSS1738 | -5.1 |
| BPSS1764 | 4.8 |  | BPSL0019 | -5.1 |
| BPSS1652 | 4.8 |  | BPSL1883 | -5.1 |
| BPSS1919 | 4.8 |  | BPSS1151 | -5.0 |
| BPSS0914 | 4.8 |  | BPSS0802 | -5.0 |
| BPSS2217 | 4.7 |  | BPSS0138 | -5.0 |
| BPSS1661 | 4.7 |  | BPSL3306 | -5.0 |
| BPSS1110 | 4.7 |  | BPSL0922 | -5.0 |
| BPSL1589 | 4.7 |  | BPSL1896 | -4.9 |
| BPSL1614 | 4.6 |  | BPSS1182 | -4.9 |
| BPSL1637 | 4.6 |  | BPSL1745 | -4.9 |
| BPSS1059 | 4.6 |  | BPSL1924 | -4.8 |
| BPSS1252 | 4.5 |  | BPSL1971 | -4.8 |
| BPSL1617 | 4.5 |  | BPSL1901 | -4.8 |
| BPSS0278 | 4.5 |  | BPSS0284 | -4.7 |
| BPSL1638 | 4.4 |  | BPSL0280 | -4.7 |
| BPSS0573 | 4.4 |  | BPSL1742 | -4.7 |
| BPSL1047 | 4.4 |  | BPSL0920 | -4.7 |
| BPSS0268A | 4.4 |  | BPSS0021 | -4.6 |
| BPSS1422 | 4.3 |  | BPSS1736 | -4.6 |
| BPSS2092 | 4.3 |  | BPSL1321 | -4.6 |
| BPSS1884 | 4.3 |  | BPSL1752 | -4.6 |
| BPSS2249 | 4.3 |  | BPSL0249 | -4.6 |
| BPSL2715 | 4.3 |  | BPSL1201 | -4.6 |
| BPSL0707 | 4.3 |  | BPSL0500A | -4.6 |
| BPSS0603 | 4.3 |  | BPSL1891 | -4.5 |
| BPSS1202 | 4.3 |  | BPSS1862 | -4.5 |
| BPSS2229 | 4.3 |  | BPSL1324 | -4.5 |
| BPSS0225 | 4.3 |  | BPSL1243 | -4.5 |
| BPSS0211 | 4.2 |  | BPSL1884 | -4.5 |
| BPSL1291 | 4.2 |  | BPSL1903 | -4.5 |
| BPSS0024 | 4.2 |  | BPSL0059 | -4.5 |
| BPSL3116 | 4.2 |  | BPSL2192 | -4.4 |
| BPSS0812A | 4.2 |  | BPSL2087 | -4.4 |
| BPSL1658 | 4.2 |  | BPSL1092 | -4.4 |
| BPSS2098 | 4.1 |  | BPSL3305 | -4.4 |
| BPSL0683 | 4.1 |  | BPSS2119 | -4.4 |
| BPSS0536 | 4.1 |  | BPSL0058 | -4.3 |
| BPSS0711 | 4.0 |  | BPSL3241 | -4.3 |
| BPSL3214 | 4.0 |  | BPSS2138 | -4.3 |
| BPSL0951 | 4.0 |  | BPSS1339 | -4.3 |
| BPSL2497 | 3.9 |  | BPSL0657 | -4.3 |
| BPSS0610 | 3.9 |  | BPSS1185 | -4.3 |
| BPSS0513 | 3.9 |  | BPSL0595 | -4.3 |
| BPSL0451 | 3.8 |  | BPSL0919A | -4.3 |
| BPSS1360 | 3.8 |  | BPSL3343 | -4.2 |
| BPSL1725 | 3.8 |  | BPSL0614 | -4.2 |
| BPSL1135 | 3.8 |  | BPSL1387 | -4.2 |
| BPSL0950 | 3.8 |  | BPSS2136 | -4.2 |
| BPSS0535 | 3.8 |  | BPSS1284 | -4.2 |
| BPSS1359 | 3.8 |  | BPSS1445 | -4.2 |
| BPSL0684 | 3.8 |  | BPSS1140 | -4.1 |
| BPSS1753 | 3.7 |  | BPSL1591 | -4.1 |
| BPSL1958 | 3.7 |  | BPSL3412 | -4.1 |
| BPSS1352 | 3.7 |  | BPSS1735 | -4.1 |
| BPSL1720 | 3.7 |  | BPSS0726 | -4.1 |
| BPSL2035 | 3.7 |  | BPSS0586 | -4.1 |
| BPSL2919 | 3.7 |  | BPSS1150 | -4.1 |
| BPSS1358 | 3.7 |  | BPSL1892 | -4.1 |
| BPSS2240 | 3.6 |  | BPSL3341 | -4.0 |
| BPSS1472 | 3.6 |  | BPSL0256 | -4.0 |
| BPSS2204 | 3.6 |  | BPSS1583 | -4.0 |
| BPSS2220 | 3.6 |  | BPSL2314 | -4.0 |
| BPSL1726 | 3.6 |  | BPSL0923 | -4.0 |
| BPSL1390 | 3.6 |  | BPSS0579 | -4.0 |
| BPSS0878 | 3.6 |  | BPSS1153 | -3.9 |
| BPSS2221 | 3.6 |  | BPSS1329 | -3.9 |
| BPSS2080 | 3.6 |  | BPSL0735 | -3.9 |
| BPSS0425 | 3.6 |  | BPSL0275 | -3.9 |
| BPSL1642 | 3.5 |  | BPSS2137 | -3.9 |
| BPSS1903 | 3.5 |  | BPSL0656 | -3.9 |
| BPSL0682 | 3.5 |  | BPSS1774A | -3.8 |
| BPSS1429 | 3.5 |  | BPSL1976 | -3.8 |
| BPSS0668 | 3.5 |  | BPSL0268 | -3.8 |
| BPSL1619 | 3.5 |  | BPSL0371 | -3.8 |
| BPSL0091 | 3.5 |  | BPSS0728 | -3.8 |
| BPSL1043 | 3.5 |  | BPSL2006 | -3.7 |
| BPSL0584 | 3.4 |  | BPSL2086 | -3.7 |
| BPSS1481 | 3.4 |  | BPSS2142 | -3.7 |
| BPSS2248 | 3.4 |  | BPSL3036 | -3.7 |
| BPSS2246 | 3.4 |  | BPSL0267 | -3.7 |
| BPSL2709 | 3.4 |  | BPSS2265 | -3.7 |
| BPSS2097 | 3.4 |  | BPSL1535 | -3.7 |
| BPSS2102 | 3.4 |  | BPSL3248 | -3.7 |
| BPSL2599 | 3.3 |  | BPSS0981 | -3.6 |
| BPSL0812 | 3.3 |  | BPSS0807 | -3.6 |
| BPSS1109 | 3.3 |  | BPSS0933 | -3.6 |
| BPSL3088 | 3.3 |  | BPSS0233 | -3.6 |
| BPSL3238 | 3.3 |  | BPSS1446 | -3.6 |
| BPSS1482 | 3.3 |  | BPSS1218 | -3.6 |
| BPSS1198 | 3.3 |  | BPSS0982 | -3.6 |
| BPSS1904 | 3.3 |  | BPSL0496 | -3.6 |
| BPSL0581 | 3.3 |  | BPSL3176 | -3.6 |
| BPSL0506 | 3.2 |  | BPSL0616 | -3.6 |
| BPSS1203 | 3.2 |  | BPSL3378 | -3.6 |
| BPSS1250 | 3.2 |  | BPSL1544 | -3.6 |
| BPSS0049 | 3.2 |  | BPSL1019 | -3.5 |
| BPSL0021 | 3.2 |  | BPSL2315 | -3.5 |
| BPSS0770 | 3.2 |  | BPSS1385A | -3.5 |
| BPSL1582 | 3.2 |  | BPSS0285 | -3.5 |
| BPSS1261 | 3.2 |  | BPSL3308 | -3.5 |
| BPSL0814 | 3.2 |  | BPSS1336 | -3.5 |
| BPSS2096 | 3.2 |  | BPSS1571 | -3.5 |
| BPSL1376 | 3.2 |  | BPSS1337 | -3.5 |
| BPSS2021 | 3.2 |  | BPSS0137 | -3.5 |
| BPSL0332 | 3.2 |  | BPSS1338 | -3.4 |
| BPSS0428 | 3.1 |  | BPSL2026 | -3.4 |
| BPSS2212 | 3.1 |  | BPSS0578 | -3.4 |
| BPSS1191 | 3.1 |  | BPSL0582 | -3.4 |
| BPSS1935 | 3.1 |  | BPSS2139 | -3.4 |
| BPSS0351 | 3.1 |  | BPSL2292 | -3.4 |
| BPSS1249 | 3.1 |  | BPSL1501 | -3.4 |
| BPSL1724 | 3.1 |  | BPSL2432 | -3.4 |
| BPSL3339 | 3.1 |  | BPSS1165 | -3.4 |
| BPSS0518 | 3.1 |  | BPSL1754 | -3.4 |
| BPSL2526 | 3.1 |  | BPSL1185 | -3.4 |
| BPSL0335 | 3.1 |  | BPSL3303 | -3.4 |
| BPSL0915 | 3.1 |  | BPSL0615 | -3.4 |
| BPSL0326 | 3.1 |  | BPSL3304 | -3.3 |
| BPSS1251 | 3.0 |  | BPSS1174 | -3.3 |
| BPSLs01 | 3.0 |  | BPSS1820 | -3.3 |
| BPSS0957 | 3.0 |  | BPSL2082 | -3.3 |
| BPSL2598 | 3.0 |  | BPSS0449 | -3.3 |
| BPSL0681 | 3.0 |  | BPSS0819 | -3.3 |
| BPSS0429 | 3.0 |  | BPSS1789 | -3.3 |
| BPSS2206 | 3.0 |  | BPSL3045 | -3.3 |
| BPSS0949 | 3.0 |  | BPSL2029 | -3.3 |
| BPSS2104 | 3.0 |  | BPSL1193 | -3.3 |
| BPSS0881 | 3.0 |  | BPSL0273 | -3.2 |
| BPSL0938 | 3.0 |  | BPSL3301 | -3.2 |
| BPSS2205 | 3.0 |  | BPSL1138 | -3.2 |
| BPSL0311 | 3.0 |  | BPSS1584 | -3.2 |
| BPSL1371C | 2.9 |  | BPSL0274 | -3.2 |
| BPSS2209 | 2.9 |  | BPSL1888 | -3.2 |
| BPSS1838 | 2.9 |  | BPSL1093 | -3.2 |
| BPSS0767 | 2.9 |  | BPSL1137 | -3.2 |
| BPSL1719 | 2.9 |  | BPSS0094 | -3.2 |
| BPSL1694 | 2.9 |  | BPSL2081 | -3.2 |
| BPSS1763 | 2.9 |  | BPSL3394 | -3.2 |
| BPSL2334 | 2.9 |  | BPSL0611 | -3.2 |
| BPSS1188 | 2.9 |  | BPSL2433 | -3.2 |
| BPSS1057 | 2.9 |  | BPSL2313 | -3.2 |
| BPSS1190 | 2.9 |  | BPSL2288 | -3.2 |
| BPSL1306 | 2.9 |  | BPSL0798 | -3.2 |
| BPSS0492 | 2.9 |  | BPSS1431 | -3.2 |
| BPSL2728 | 2.9 |  | BPSS1328 | -3.2 |
| BPSS0512 | 2.9 |  | BPSL3072 | -3.1 |
| BPSS1659 | 2.9 |  | BPSL0228 | -3.1 |
| BPSS2020 | 2.9 |  | BPSL0494 | -3.1 |
| BPSS2281 | 2.9 |  | BPSL1192 | -3.1 |
| BPSL2036 | 2.9 |  | BPSL0055 | -3.1 |
| BPSL1066 | 2.9 |  | BPSL3044 | -3.1 |
| BPSS0660 | 2.9 |  | BPSS1817 | -3.1 |
| BPSL2630 | 2.9 |  | BPSL2924 | -3.1 |
| BPSL1806 | 2.9 |  | BPSL0521 | -3.1 |
| BPSS2045 | 2.9 |  | BPSS1181 | -3.1 |
| BPSS2199 | 2.9 |  | BPSL0773 | -3.1 |
| BPSS0156 | 2.9 |  | BPSL1869 | -3.1 |
| BPSS0609 | 2.8 |  | BPSS1742 | -3.1 |
| BPSS2236 | 2.8 |  | BPSL1388 | -3.1 |
| BPSS0748 | 2.8 |  | BPSL2594 | -3.1 |
| BPSS2247 | 2.8 |  | BPSL2146 | -3.1 |
| BPSS1102 | 2.8 |  | BPSL1418 | -3.1 |
| BPSS1194 | 2.8 |  | BPSL2555 | -3.1 |
| BPSLt17 | 2.8 |  | BPSL1319 | -3.1 |
| BPSS2100 | 2.8 |  | BPSL2997 | -3.1 |
| BPSS1312 | 2.7 |  | BPSL3050 | -3.1 |
| BPSS2250 | 2.7 |  | BPSL2923 | -3.1 |
| BPSS0304 | 2.7 |  | BPSL1904 | -3.1 |
| BPSS1747 | 2.7 |  | BPSL1186 | -3.1 |
| BPSS1193 | 2.7 |  | BPSL1386 | -3.0 |
| BPSS2099 | 2.7 |  | BPSS1383a | -3.0 |
| BPSS1918 | 2.7 |  | BPSL2639 | -3.0 |
| BPSL0062 | 2.7 |  | BPSS1283 | -3.0 |
| BPSL1715 | 2.7 |  | BPSS2268 | -3.0 |
| BPSS0533 | 2.7 |  | BPSL0972 | -3.0 |
| BPSL1549 | 2.7 |  | BPSL0742 | -3.0 |
| BPSS1889 | 2.7 |  | BPSL2369 | -3.0 |
| BPSL3096 | 2.7 |  | BPSL1484 | -3.0 |
| BPSL1716 | 2.7 |  | BPSS1176 | -3.0 |
| BPSS2218 | 2.7 |  | BPSL3302 | -3.0 |
| BPSL3104 | 2.7 |  | BPSL1268 | -3.0 |
| BPSL2489 | 2.7 |  | BPSS1432 | -2.9 |
| BPSL1708A | 2.7 |  | BPSL0874 | -2.9 |
| BPSS1372 | 2.7 |  | BPSL1886 | -2.9 |
| BPSS1837 | 2.6 |  | BPSL3249 | -2.9 |
| BPSL1377 | 2.6 |  | BPSS1146 | -2.9 |
| BPSS0358 | 2.6 |  | BPSS1772 | -2.9 |
| BPSL1279 | 2.6 |  | BPSL0103 | -2.9 |
| BPSS2093 | 2.6 |  | BPSL3349 | -2.9 |
| BPSS1928 | 2.6 |  | BPSS0207 | -2.9 |
| BPSS0615 | 2.6 |  | BPSS0391 | -2.9 |
| BPSS1058 | 2.6 |  | BPSS0612 | -2.9 |
| BPSL2868 | 2.6 |  | BPSL1572 | -2.9 |
| BPSS1128 | 2.6 |  | BPSS0485 | -2.9 |
| BPSS2004 | 2.6 |  | BPSL1383 | -2.9 |
| BPSS1271 | 2.6 |  | BPSS0576 | -2.9 |
| BPSS0758 | 2.6 |  | BPSL2699 | -2.9 |
| BPSS1480 | 2.6 |  | BPSL2289 | -2.9 |
| BPSS2241 | 2.6 |  | BPSS1958 | -2.8 |
| BPSL2725 | 2.6 |  | BPSL1191 | -2.8 |
| BPSL0452 | 2.6 |  | BPSL0232 | -2.8 |
| BPSS2025 | 2.6 |  | BPSS1185a | -2.8 |
| BPSL0098 | 2.6 |  | BPSL3313 | -2.8 |
| BPSL2665 | 2.6 |  | BPSL2845 | -2.8 |
| BPSL2894 | 2.6 |  | BPSL2290 | -2.8 |
| BPSL1723 | 2.6 |  | BPSL1384 | -2.8 |
| BPSL1041 | 2.6 |  | BPSL0276 | -2.8 |
| BPSS1192 | 2.6 |  | BPSS0884 | -2.8 |
| BPSS0045 | 2.5 |  | BPSS0101 | -2.8 |
| BPSL1793 | 2.5 |  | BPSS0837 | -2.8 |
| BPSL2918 | 2.5 |  | BPSS1335 | -2.8 |
| BPSL1708 | 2.5 |  | BPSL0122 | -2.8 |
| BPSS1200 | 2.5 |  | BPSS0810 | -2.8 |
| BPSS1909 | 2.5 |  | BPSS0801 | -2.8 |
| BPSL2444 | 2.5 |  | BPSL1595 | -2.8 |
| BPSS2348 | 2.5 |  | BPSL0988 | -2.8 |
| BPSL0221 | 2.5 |  | BPSL2589 | -2.7 |
| BPSS1129 | 2.5 |  | BPSL2844 | -2.7 |
| BPSL2628 | 2.5 |  | BPSS1147 | -2.7 |
| BPSL0200 | 2.5 |  | BPSS1743 | -2.7 |
| BPSL0024 | 2.5 |  | BPSL0495 | -2.7 |
| BPSL2701 | 2.5 |  | BPSL0678 | -2.7 |
| BPSL0916 | 2.5 |  | BPSS1866 | -2.7 |
| BPSLs02 | 2.5 |  | BPSL2084A | -2.7 |
| BPSS1280 | 2.5 |  | BPSS0484 | -2.7 |
| BPSL0871 | 2.5 |  | BPSL3083 | -2.7 |
| BPSL1578 | 2.5 |  | BPSL1194 | -2.7 |
| BPSS2091 | 2.5 |  | BPSL0504 | -2.7 |
| BPSS1712 | 2.5 |  | BPSL1522 | -2.7 |
| BPSS1585 | 2.5 |  | BPSS1815 | -2.7 |
| BPSS0537 | 2.5 |  | BPSL3245 | -2.7 |
| BPSS1375 | 2.5 |  | BPSS1145 | -2.7 |
| BPSS1357 | 2.5 |  | BPSL1741 | -2.7 |
| BPSL0513 | 2.5 |  | BPSL1805 | -2.7 |
| BPSL1173 | 2.5 |  | BPSS1180 | -2.7 |
| BPSS0607 | 2.5 |  | BPSS1177 | -2.7 |
| BPSS0035 | 2.5 |  | BPSS0820 | -2.7 |
| BPSS0633 | 2.5 |  | BPSL1505 | -2.7 |
| BPSS1114 | 2.5 |  | BPSS2038 | -2.7 |
| BPSL0075a | 2.4 |  | BPSL0176 | -2.7 |
| BPSS0961 | 2.4 |  | BPSL0226 | -2.7 |
| BPSS1197 | 2.4 |  | BPSL0574 | -2.7 |
| BPSS0159 | 2.4 |  | BPSL1506 | -2.7 |
| BPSL0293 | 2.4 |  | BPSL3336 | -2.7 |
| BPSL0514 | 2.4 |  | BPSS1340 | -2.7 |
| BPSS1758 | 2.4 |  | BPSL3161 | -2.7 |
| BPSL0347 | 2.4 |  | BPSL0613 | -2.6 |
| BPSS1351 | 2.4 |  | BPSL0669 | -2.6 |
| BPSS2094 | 2.4 |  | BPSS0688 | -2.6 |
| BPSL2093 | 2.4 |  | BPSL1887 | -2.6 |
| BPSL2418 | 2.4 |  | BPSL3043 | -2.6 |
| BPSL0099 | 2.4 |  | BPSS0192 | -2.6 |
| BPSS0605 | 2.4 |  | BPSL3178 | -2.6 |
| BPSL1476 | 2.4 |  | BPSL1844 | -2.6 |
| BPSL0090 | 2.4 |  | BPSL3350 | -2.6 |
| BPSS2224 | 2.4 |  | BPSS0092 | -2.6 |
| BPSL0680 | 2.4 |  | BPSS1173 | -2.6 |
| BPSS1703 | 2.4 |  | BPSL0141 | -2.6 |
| BPSL1608 | 2.4 |  | BPSL1187 | -2.6 |
| BPSL3277 | 2.4 |  | BPSL2651 | -2.6 |
| BPSL1758 | 2.4 |  | BPSL1366 | -2.6 |
| BPSL2032 | 2.4 |  | BPSS0386 | -2.6 |
| BPSS1655 | 2.4 |  | BPSL3321 | -2.6 |
| BPSL0458a | 2.4 |  | BPSS0017 | -2.6 |
| BPSL3186 | 2.4 |  | BPSS0276 | -2.6 |
| BPSS0302 | 2.4 |  | BPSL2434 | -2.6 |
| BPSL1110 | 2.4 |  | BPSL0469 | -2.6 |
| BPSL2990 | 2.4 |  | BPSL2087A | -2.6 |
| BPSL1246 | 2.4 |  | BPSS1211 | -2.6 |
| BPSL1579 | 2.4 |  | BPSL2435 | -2.6 |
| BPSS0959 | 2.4 |  | BPSS0821 | -2.6 |
| BPSL2727 | 2.4 |  | BPSS0691 | -2.6 |
| BPSS1048 | 2.4 |  | BPSS0008 | -2.6 |
| BPSL2040 | 2.4 |  | BPSL0015 | -2.6 |
| BPSL1712 | 2.4 |  | BPSS0898 | -2.6 |
| BPSS0564 | 2.3 |  | BPSL3077 | -2.6 |
| BPSS1902 | 2.3 |  | BPSS0613 | -2.6 |
| BPSL3239 | 2.3 |  | BPSS0390 | -2.6 |
| BPSS2000 | 2.3 |  | BPSL2122 | -2.5 |
| BPSL0063 | 2.3 |  | BPSL0142 | -2.5 |
| BPSS2172 | 2.3 |  | BPSS0354 | -2.5 |
| BPSS0845 | 2.3 |  | BPSS1405 | -2.5 |
| BPSL0448 | 2.3 |  | BPSS1470 | -2.5 |
| BPSL1774 | 2.3 |  | BPSS1282 | -2.5 |
| BPSS0852 | 2.3 |  | BPSS0283 | -2.5 |
| BPSS0408 | 2.3 |  | BPSS0469 | -2.5 |
| BPSS0287 | 2.3 |  | BPSS0254 | -2.5 |
| BPSL3108 | 2.3 |  | BPSS0611 | -2.5 |
| BPSS0676 | 2.3 |  | BPSL0532 | -2.5 |
| BPSL3119 | 2.3 |  | BPSS0096 | -2.5 |
| BPSLt30 | 2.3 |  | BPSS0546 | -2.5 |
| BPSL2143 | 2.3 |  | BPSS1729 | -2.5 |
| BPSS2005 | 2.3 |  | BPSL0612 | -2.5 |
| BPSL2470A | 2.3 |  | BPSL3233 | -2.5 |
| BPSL0596 | 2.3 |  | BPSS1553 | -2.5 |
| BPSL2911 | 2.3 |  | BPSS0126 | -2.5 |
| BPSS1302 | 2.3 |  | BPSS1034 | -2.5 |
| BPSL1785 | 2.3 |  | BPSL1534 | -2.5 |
| BPSL1683 | 2.3 |  | BPSL0741 | -2.5 |
| BPSL3223 | 2.3 |  | BPSL0356 | -2.5 |
| BPSL0516 | 2.3 |  | BPSS1821 | -2.5 |
| BPSL1717 | 2.3 |  | BPSL1879 | -2.5 |
| BPSL1704 | 2.3 |  | BPSL1074 | -2.4 |
| BPSS0976 | 2.3 |  | BPSL1156 | -2.4 |
| BPSS1633 | 2.3 |  | BPSL2804 | -2.4 |
| BPSL2134 | 2.3 |  | BPSL2025 | -2.4 |
| BPSL3120 | 2.3 |  | BPSL1977 | -2.4 |
| BPSL3222 | 2.3 |  | BPSL0181 | -2.4 |
| BPSL3318 | 2.3 |  | BPSS0910 | -2.4 |
| BPSL2052 | 2.3 |  | BPSS1144 | -2.4 |
| BPSL3095 | 2.3 |  | BPSS0636 | -2.4 |
| BPSS2340 | 2.3 |  | BPSL0774 | -2.4 |
| BPSL0457 | 2.3 |  | BPSL0391 | -2.4 |
| BPSL2033 | 2.3 |  | BPSS0577 | -2.4 |
| BPSS1455 | 2.3 |  | BPSL2070 | -2.4 |
| BPSS0557 | 2.3 |  | BPSS0296 | -2.4 |
| BPSS1632 | 2.3 |  | BPSL0084 | -2.4 |
| BPSS1047a | 2.3 |  | BPSL0530 | -2.4 |
| BPSL1056 | 2.2 |  | BPSL2866 | -2.4 |
| BPSL1427 | 2.2 |  | BPSS1707 | -2.4 |
| BPSS2101 | 2.2 |  | BPSL2332 | -2.4 |
| BPSS2347 | 2.2 |  | BPSL1902 | -2.4 |
| BPSL0314 | 2.2 |  | BPSL1833 | -2.4 |
| BPSS1195 | 2.2 |  | BPSL0277 | -2.4 |
| BPSL1015 | 2.2 |  | BPSL2690 | -2.4 |
| BPSL3056 | 2.2 |  | BPSL3353 | -2.4 |
| BPSSt02 | 2.2 |  | BPSS0623 | -2.4 |
| BPSS2239 | 2.2 |  | BPSS0143 | -2.3 |
| BPSL2910 | 2.2 |  | BPSS0694 | -2.3 |
| BPSL2717 | 2.2 |  | BPSL0227 | -2.3 |
| BPSL1371B | 2.2 |  | BPSL1500 | -2.3 |
| BPSL0350 | 2.2 |  | BPSS0005 | -2.3 |
| BPSL0359 | 2.2 |  | BPSL1523 | -2.3 |
| BPSL0348 | 2.2 |  | BPSS0112 | -2.3 |
| BPSL1359 | 2.2 |  | BPSL2030 | -2.3 |
| BPSS0153 | 2.2 |  | BPSL0093 | -2.3 |
| BPSS0608 | 2.2 |  | BPSL0675 | -2.3 |
| BPSL0218 | 2.2 |  | BPSL2824 | -2.3 |
| BPSL2638 | 2.2 |  | BPSS0687 | -2.3 |
| BPSS1680 | 2.2 |  | BPSS0575 | -2.3 |
| BPSS0158 | 2.2 |  | BPSS0006 | -2.3 |
| BPSS1349 | 2.2 |  | BPSS1330 | -2.3 |
| BPSL3276 | 2.2 |  | BPSL1018 | -2.3 |
| BPSL2958 | 2.2 |  | BPSL3294 | -2.3 |
| BPSL0815 | 2.2 |  | BPSL3335 | -2.3 |
| BPSL2591 | 2.2 |  | BPSL0425 | -2.3 |
| BPSL0065 | 2.2 |  | BPSS1556 | -2.3 |
| BPSS0271 | 2.2 |  | BPSL1007 | -2.3 |
| BPSL1657 | 2.2 |  | BPSS1784 | -2.3 |
| BPSL1132 | 2.2 |  | BPSL0123 | -2.3 |
| BPSL1711 | 2.2 |  | BPSL0289 | -2.3 |
| BPSL1709 | 2.2 |  | BPSS0322 | -2.3 |
| BPSS0303 | 2.2 |  | BPSS0483 | -2.3 |
| BPSS2232 | 2.2 |  | BPSL2287 | -2.3 |
| BPSS0930 | 2.2 |  | BPSL0180 | -2.3 |
| BPSL2703 | 2.2 |  | BPSL0116 | -2.3 |
| BPSS0977 | 2.2 |  | BPSL2318 | -2.3 |
| BPSS1783 | 2.2 |  | BPSL2147 | -2.3 |
| BPSL2496 | 2.2 |  | BPSS1816 | -2.3 |
| BPSS1045 | 2.2 |  | BPSS1667 | -2.3 |
| BPSL1381 | 2.2 |  | BPSL1091 | -2.3 |
| BPSL1374 | 2.2 |  | BPSL0665 | -2.3 |
| BPSL2550 | 2.2 |  | BPSL2568 | -2.3 |
| BPSL1943 | 2.1 |  | BPSS1434 | -2.3 |
| BPSL0260 | 2.1 |  | BPSS0073 | -2.3 |
| BPSS1634 | 2.1 |  | BPSL1228 | -2.3 |
| BPSS0534 | 2.1 |  | BPSL3126 | -2.3 |
| BPSS1761 | 2.1 |  | BPSL2498 | -2.3 |
| BPSS2231 | 2.1 |  | BPSS0077 | -2.2 |
| BPSS1231 | 2.1 |  | BPSS0392 | -2.2 |
| BPSS2056 | 2.1 |  | BPSS2039 | -2.2 |
| BPSL3004 | 2.1 |  | BPSL2756 | -2.2 |
| BPSS2211 | 2.1 |  | BPSL3320 | -2.2 |
| BPSL2051 | 2.1 |  | BPSS0467 | -2.2 |
| BPSL1543 | 2.1 |  | BPSL0924 | -2.2 |
| BPSS0679 | 2.1 |  | BPSS1818 | -2.2 |
| BPSS1196 | 2.1 |  | BPSL2257 | -2.2 |
| BPSL2917 | 2.1 |  | BPSL1552 | -2.2 |
| BPSL3202 | 2.1 |  | BPSL3293 | -2.2 |
| BPSL1850 | 2.1 |  | BPSS2120 | -2.2 |
| BPSS2226 | 2.1 |  | BPSS1726 | -2.2 |
| BPSL1463 | 2.1 |  | BPSL1845 | -2.2 |
| BPSS1840 | 2.1 |  | BPSL1769 | -2.2 |
| BPSS2105 | 2.1 |  | BPSL0733 | -2.2 |
| BPSL2888 | 2.1 |  | BPSL0272 | -2.2 |
| BPSL1580 | 2.1 |  | BPSL2543 | -2.2 |
| BPSL3005 | 2.1 |  | BPSL0085 | -2.2 |
| BPSL2876 | 2.1 |  | BPSS1974 | -2.2 |
| BPSS0614 | 2.1 |  | BPSL1440 | -2.2 |
| BPSL0884 | 2.1 |  | BPSL2689 | -2.2 |
| BPSL2954 | 2.1 |  | BPSL0271 | -2.2 |
| BPSS0306 | 2.1 |  | BPSL0028 | -2.2 |
| BPSS1656 | 2.1 |  | BPSL1818 | -2.2 |
| BPSL1738 | 2.1 |  | BPSL2001 | -2.2 |
| BPSL3286 | 2.1 |  | BPSS0031 | -2.2 |
| BPSL3282 | 2.1 |  | BPSS0695 | -2.2 |
| BPSS2300 | 2.1 |  | BPSL0052 | -2.2 |
| BPSL0076 | 2.1 |  | BPSL3258 | -2.2 |
| BPSS0300 | 2.1 |  | BPSL2546 | -2.2 |
| BPSLt15 | 2.1 |  | BPSS1559 | -2.2 |
| BPSS2044 | 2.1 |  | BPSL2145 | -2.2 |
| BPSS1797 | 2.1 |  | BPSS0740 | -2.2 |
| BPSL1305 | 2.1 |  | BPSL0536 | -2.2 |
| BPSL3213 | 2.1 |  | BPSL2765 | -2.2 |
| BPSL3275 | 2.1 |  | BPSL1424 | -2.2 |
| BPSS0157 | 2.1 |  | BPSL3259 | -2.2 |
| BPSS0046 | 2.1 |  | BPSL0121 | -2.2 |
| BPSL0097 | 2.1 |  | BPSL2499 | -2.2 |
| BPSS1056 | 2.1 |  | BPSL2144 | -2.2 |
| BPSS2227 | 2.1 |  | BPSL0056 | -2.2 |
| BPSS0975 | 2.1 |  | BPSL0253 | -2.2 |
| BPSL1112 | 2.1 |  | BPSS1117 | -2.2 |
| BPSS1168 | 2.1 |  | BPSL0031 | -2.2 |
| BPSS1364a | 2.1 |  | BPSL3325 | -2.2 |
| BPSL0296 | 2.1 |  | BPSL3166 | -2.2 |
| BPSL2170 | 2.1 |  | BPSS0312 | -2.2 |
| BPSL3117 | 2.1 |  | BPSL3324 | -2.1 |
| BPSL1764 | 2.0 |  | BPSL1466 | -2.1 |
| BPSL1111 | 2.0 |  | BPSS0808 | -2.1 |
| BPSS0263 | 2.0 |  | BPSL1519 | -2.1 |
| BPSS1757 | 2.0 |  | BPSL0125 | -2.1 |
| BPSS1604 | 2.0 |  | BPSL2083 | -2.1 |
| BPSL2597 | 2.0 |  | BPSS0088 | -2.1 |
| BPSS1112 | 2.0 |  | BPSL0569 | -2.1 |
| BPSL2239 | 2.0 |  | BPSL3165 | -2.1 |
| BPSS1754 | 2.0 |  | BPSL1008 | -2.1 |
| BPSS0013 | 2.0 |  | BPSS0454 | -2.1 |
| BPSS1453 | 2.0 |  | BPSS0620 | -2.1 |
| BPSS0402B | 2.0 |  | BPSL0212A | -2.1 |
| BPSS0539 | 2.0 |  | BPSS0468 | -2.1 |
| BPSS0598 | 2.0 |  | BPSL1521 | -2.1 |
| BPSS0958 | 2.0 |  | BPSL0127 | -2.1 |
| BPSL0302 | 2.0 |  | BPSL2331 | -2.1 |
| BPSL2167 | 2.0 |  | BPSS1143 | -2.1 |
| BPSS0597 | 2.0 |  | BPSL2088 | -2.1 |
| BPSL0033 | 2.0 |  | BPSL1832 | -2.1 |
| BPSL2606 | 2.0 |  | BPSS1588 | -2.1 |
| BPSS0967 | 2.0 |  | BPSL0573 | -2.1 |
| BPSS0724 | 2.0 |  | BPSL0230 | -2.1 |
| BPSL0586a | 2.0 |  | BPSL1318 | -2.1 |
| BPSL2445 | 2.0 |  | BPSS0617 | -2.1 |
| BPSL2716 | 2.0 |  | BPSS1288 | -2.1 |
| BPSL2377 | 2.0 |  | BPSL3031 | -2.1 |
| BPSL0862 | 2.0 |  | BPSL2863 | -2.1 |
| BPSS0675 | 2.0 |  | BPSL1280 | -2.1 |
| BPSL2641 | 2.0 |  | BPSL0867 | -2.1 |
| BPSL0450 | 2.0 |  | BPSS0234 | -2.1 |
| BPSL2250 | 2.0 |  | BPSL0030 | -2.1 |
| BPSS1755 | 2.0 |  | BPSS2173 | -2.1 |
| BPSLt42 | 2.0 |  | BPSL0947 | -2.1 |
| BPSL0286 | 2.0 |  | BPSS0078 | -2.1 |
| BPSL2928 | 2.0 |  | BPSS2074 | -2.1 |
| BPSL0993 | 2.0 |  | BPSL0743 | -2.1 |
| BPSL1553 | 2.0 |  | BPSS0108 | -2.1 |
|  |  |  | BPSL0661 | -2.1 |
|  |  |  | BPSL3164 | -2.1 |
|  |  |  | BPSS0470 | -2.1 |
|  |  |  | BPSL2682 | -2.1 |
|  |  |  | BPSL2295 | -2.1 |
|  |  |  | BPSL0662 | -2.1 |
|  |  |  | BPSL3051 | -2.1 |
|  |  |  | BPSS0364 | -2.1 |
|  |  |  | BPSS1607 | -2.1 |
|  |  |  | BPSL0523 | -2.1 |
|  |  |  | BPSL1817 | -2.1 |
|  |  |  | BPSS0139 | -2.1 |
|  |  |  | BPSL0229 | -2.1 |
|  |  |  | BPSS0809 | -2.1 |
|  |  |  | BPSL0638 | -2.1 |
|  |  |  | BPSS0080 | -2.1 |
|  |  |  | BPSS1768 | -2.1 |
|  |  |  | BPSS1255 | -2.1 |
|  |  |  | BPSS1810 | -2.1 |
|  |  |  | BPSS1779 | -2.1 |
|  |  |  | BPSS0369 | -2.1 |
|  |  |  | BPSL0789 | -2.1 |
|  |  |  | BPSL3326 | -2.1 |
|  |  |  | BPSL0841 | -2.1 |
|  |  |  | BPSL0378 | -2.1 |
|  |  |  | BPSS0481 | -2.1 |
|  |  |  | BPSL1819 | -2.1 |
|  |  |  | BPSL0649 | -2.1 |
|  |  |  | BPSS0093 | -2.1 |
|  |  |  | BPSL1325 | -2.1 |
|  |  |  | BPSL0549A | -2.1 |
|  |  |  | BPSL1121 | -2.0 |
|  |  |  | BPSL0254 | -2.0 |
|  |  |  | BPSL2188 | -2.0 |
|  |  |  | BPSL0252 | -2.0 |
|  |  |  | BPSL1750 | -2.0 |
|  |  |  | BPSS1213 | -2.0 |
|  |  |  | BPSL2615 | -2.0 |
|  |  |  | BPSS0486 | -2.0 |
|  |  |  | BPSL0027 | -2.0 |
|  |  |  | BPSS1447 | -2.0 |
|  |  |  | BPSL1358 | -2.0 |
|  |  |  | BPSL1841 | -2.0 |
|  |  |  | BPSL2024 | -2.0 |
|  |  |  | BPSS2308 | -2.0 |
|  |  |  | BPSL1593 | -2.0 |
|  |  |  | BPSL0747 | -2.0 |
|  |  |  | BPSL0618 | -2.0 |
|  |  |  | BPSL3138 | -2.0 |
|  |  |  | BPSL0608 | -2.0 |
|  |  |  | BPSL0619 | -2.0 |
|  |  |  | BPSL3342 | -2.0 |
|  |  |  | BPSS0624 | -2.0 |
|  |  |  | BPSL1188 | -2.0 |
|  |  |  | BPSL3028 | -2.0 |
|  |  |  | BPSS1776 | -2.0 |
|  |  |  | BPSL0840 | -2.0 |
|  |  |  | BPSL0653 | -2.0 |
|  |  |  | BPSL2998 | -2.0 |
|  |  |  | BPSL0748 | -2.0 |
|  |  |  | BPSL1267 | -2.0 |
|  |  |  | BPSL0535 | -2.0 |
|  |  |  | BPSS0692 | -2.0 |
