# Supplementary material for: The Condition-Dependent Transcriptional Landscape of Burkholderia pseudomallei
Source: PLoS Genet. 2013 Sep 12;9(9):e1003795. doi: 10.1371/journal.pgen.1003795 (PMC3772027; doi:10.1371/journal.pgen.1003795)
Supplement: Table S14 — Quorum-sensing (QS) associated genes found in literature. (DOC) [file pgen.1003795.s022.doc]

Table S14: Quorum-sensing (QS) associated genes found in literature. Genes marked with an asterisk (*) are genes that have been expressed in the QS signature of our study.

| **#** | **SangerID** | **Gene** | **Description** | **Author** | **Year** | **Publication** |
| --- | --- | --- | --- | --- | --- | --- |
| **1** | BPSS0885 | *pmlI* | N-acylhomoserine lactone synthase | Ulrich et. al. | 2004 | J. Medical Microbiology |
| ***2** | BPSS1180 | *bpsI2* | N-acylhomoserine lactone synthase | Ulrich et. al. | 2004 | J. Medical Microbiology |
| **3** | BPSS1570 | *bpsI3* | N-acylhomoserine lactone synthase | Ulrich et. al. | 2004 | J. Medical Microbiology |
| **4** | BPSS0887 | *pmlR* | N-acylhomoserine lactone dependent regulatory protein | Ulrich et. al. | 2004 | J. Medical Microbiology |
| ***5** | BPSS1176 | *bpsR2* | N-acylhomoserine lactone dependent regulatory protein | Ulrich et. al. | 2004 | J. Medical Microbiology |
| **6** | BPSS1569 | *bpsR3* | N-acylhomoserine lactone-dependent regulatory protein | Ulrich et. al. | 2004 | J. Medical Microbiology |
| ***7** | BPSS0312 | *bpsR4* | LuxR family transcriptional regulator | Ulrich et. al. | 2004 | J. Medical Microbiology |
| **8** | BPSL2347 | *bpsR5* | LuxR family transcriptional regulator | Ulrich et. al. | 2004 | J. Medical Microbiology |
| ***9** | BPSL1505 | *rpoS* | RNA polymerase sigma factor RpoS | Wongtrakoongate et. al. | 2012 | Microbiol Immunol |
| **10** | BPSL3029 | *murF* | UDP-N-acetylmuramyl pentapeptide synthase | Wongtrakoongate et. al. | 2012 | Microbiol Immunol |
| **11** | BPSS1679 | *ompC* | porin related exported protein | Wongtrakoongate et. al. | 2012 | Microbiol Immunol |
| ***12** | BPSL1049 | *-* | Phosphatidylethanolamine-binding protein | Wongtrakoongate et. al. | 2012 | Microbiol Immunol |
| **13** | BPSS2003 | *-* | Metal-binding periplasmic protein | Wongtrakoongate et. al. | 2012 | Microbiol Immunol |
| **14** | BPSS0851 | *ntaA* | Nitrilotriacetate monooxygenase A | Wongtrakoongate et. al. | 2012 | Microbiol Immunol |
| **15** | BPSS1281 | *ppk2* | Polyphosphate kinase 2 family | Wongtrakoongate et. al. | 2012 | Microbiol Immunol |
| ***16** | BPSL2952 | *gapA* | Glyceraldehyde 3-phosphate dehydrogenase | Wongtrakoongate et. al. | 2012 | Microbiol Immunol |
| **17** | BPSL2613 | *pgl* | Phosphogluconolactonase | Wongtrakoongate et. al. | 2012 | Microbiol Immunol |
| **18** | BPSL1955 | *scoA* | Succinyl-CoA:3-ketoacid-coenzyme A transferase subunit A | Wongtrakoongate et. al. | 2012 | Microbiol Immunol |
| **19** | BPSS1274 | *acs* | Acyl-CoA synthetases (AMP-forming)/AMP-acid ligases II | Wongtrakoongate et. al. | 2012 | Microbiol Immunol |
| **20** | BPSL0589 | *pcaC* | Carboxymuconolactone decarboxylase | Wongtrakoongate et. al. | 2012 | Microbiol Immunol |
| ***21** | BPSS1183 | *cmaB* | Non-ribosomally encoded peptide/polyketide synthase | Wongtrakoongate et. al. | 2012 | Microbiol Immunol |
| **22** | BPSL2507 | *cysM* | Cysteine synthase | Wongtrakoongate et. al. | 2012 | Microbiol Immunol |
| **23** | BPSL3134 | *hisA* | Phosphoribosyl formimino-5-aminoimidazole carboxamide ribonucleotide isomerase | Wongtrakoongate et. al. | 2012 | Microbiol Immunol |
| **24** | BPSL2169 | *dapD* | 2,3,4,5-tetrahydropyridine-2,6-dicarboxylate N-succinyltransferase | Wongtrakoongate et. al. | 2012 | Microbiol Immunol |
| **25** | BPSL0875 | *adk* | Adenylate kinase | Wongtrakoongate et. al. | 2012 | Microbiol Immunol |
| **26** | BPSL3422 | *adc* | Adenylate cyclase | Wongtrakoongate et. al. | 2012 | Microbiol Immunol |
| **27** | BPSL2480 | *mogA* | Molybdopterin biosynthesis protein | Wongtrakoongate et. al. | 2012 | Microbiol Immunol |
| **28** | BPSS0407 | *-* | Integrase core domain protein | Wongtrakoongate et. al. | 2012 | Microbiol Immunol |
| **29** | BPSS0004 | *-* | Transcriptional regulator, XRE family | Wongtrakoongate et. al. | 2012 | Microbiol Immunol |
| ***30** | BPSL1269 | *-* | Transcriptional regulator | Wongtrakoongate et. al. | 2012 | Microbiol Immunol |
| **31** | BPSL3311 | *flhD* | Transcriptional activator FlhD | Wongtrakoongate et. al. | 2012 | Microbiol Immunol |
| **32** | BPSL0004 | *hupA* | DNA-binding protein HU-alpha | Wongtrakoongate et. al. | 2012 | Microbiol Immunol |
| **33** | BPSL3215 | *tuf* | Elongation factor Tuf | Wongtrakoongate et. al. | 2012 | Microbiol Immunol |
| **34** | BPSL3188 | *rpsD* | 30S ribosomal protein S4 | Wongtrakoongate et. al. | 2012 | Microbiol Immunol |
| **35** | BPSL3196 | *rpsE* | 30S ribosomal protein S5 | Wongtrakoongate et. al. | 2012 | Microbiol Immunol |
| ***36** | BPSL2910 | *rpsI* | 30S ribosomal protein S9 | Wongtrakoongate et. al. | 2012 | Microbiol Immunol |
| **37** | BPSL3212 | *rplD* | 50S ribosomal protein L4 | Wongtrakoongate et. al. | 2012 | Microbiol Immunol |
| **38** | BPSL2698 | *groES* | Chaperonin GroES | Wongtrakoongate et. al. | 2012 | Microbiol Immunol |
| ***39** | BPSL2917 | *-* | Heat shock Hsp20-related protein | Wongtrakoongate et. al. | 2012 | Microbiol Immunol |
| **40** | BPSS2288 | *-* | HSP20/alpha crystallin family protein | Wongtrakoongate et. al. | 2012 | Microbiol Immunol |
| **41** | BPSL2246 | *ppiB* | Peptidyl-prolyl cis-trans isomerase B | Wongtrakoongate et. al. | 2012 | Microbiol Immunol |
| **42** | BPSL0525 | *-* | O-linked N-acetylglucosamine transferase | Wongtrakoongate et. al. | 2012 | Microbiol Immunol |
| **43** | BPSS1533 | *bicA* | Type III secretion chaperone LcrH/SycD | Wongtrakoongate et. al. | 2012 | Microbiol Immunol |
| ***44** | BPSL1896 | *-* | Flp pilus assembly protein | Wongtrakoongate et. al. | 2012 | Microbiol Immunol |
| **45** | BPSS2194 | *-* | TadE-like protein | Wongtrakoongate et. al. | 2012 | Microbiol Immunol |
| ***46** | BPSL3305 | *cheW* | Chemotaxis protein CheW | Wongtrakoongate et. al. | 2012 | Microbiol Immunol |
| **47** | BPSL2298 | *phaP* | Phasin | Wongtrakoongate et. al. | 2012 | Microbiol Immunol |
| **48** | BPSL2097 | *-* | Alkyl hydroperoxide reductase D | Wongtrakoongate et. al. | 2012 | Microbiol Immunol |
| **49** | BPSL2748 | *-* | Oxidoreductase | Wongtrakoongate et. al. | 2012 | Microbiol Immunol |
| **50** | BPSS1782 | *ohr* | Organic hydroperoxide resistance protein | Wongtrakoongate et. al. | 2012 | Microbiol Immunol |
| ***51** | BPSS1958 | *-* | Osmotically inducible Y domain protein | Wongtrakoongate et. al. | 2012 | Microbiol Immunol |
| **52** | BPSS1924 | *-* | Osmotically inducible Y domain protein | Wongtrakoongate et. al. | 2012 | Microbiol Immunol |
| **53** | BPSL1467 | *-* | Bacterioferritin antioxidant protein | Wongtrakoongate et. al. | 2012 | Microbiol Immunol |
| **54** | BPSS1506 | *-* | Uncharacterized lipoprotein | Wongtrakoongate et. al. | 2012 | Microbiol Immunol |
| **55** | BPSS0132 | *-* | Limonene-1,2-epoxide hydrolase | Wongtrakoongate et. al. | 2012 | Microbiol Immunol |
| ***56** | BPSL0348 | *-* | Hypothetical protein | Wongtrakoongate et. al. | 2012 | Microbiol Immunol |
| **57** | BPSL0349 | *-* | Hypothetical protein | Wongtrakoongate et. al. | 2012 | Microbiol Immunol |
| **58** | BPSL0599 | *-* | Hypothetical protein | Wongtrakoongate et. al. | 2012 | Microbiol Immunol |
| ***59** | BPSL1549 | *-* | Hypothetical protein | Wongtrakoongate et. al. | 2012 | Microbiol Immunol |
| **60** | BPSL1601 | *-* | Hypothetical protein | Wongtrakoongate et. al. | 2012 | Microbiol Immunol |
| **61** | BPSL3012 | *-* | Hypothetical protein | Wongtrakoongate et. al. | 2012 | Microbiol Immunol |
| **62** | BPSS0212 | *-* | Hypothetical protein | Wongtrakoongate et. al. | 2012 | Microbiol Immunol |
| **63** | BPSS0213 | *-* | Hypothetical protein | Wongtrakoongate et. al. | 2012 | Microbiol Immunol |
| **64** | BPSS0683 | *-* | Hypothetical protein | Wongtrakoongate et. al. | 2012 | Microbiol Immunol |
| ***65** | BPSS1588 | *-* | Hypothetical protein | Wongtrakoongate et. al. | 2012 | Microbiol Immunol |
| **66** | BPSS2055 | *-* | Hypothetical protein | Wongtrakoongate et. al. | 2012 | Microbiol Immunol |
| **67** | BPSS2129 | *-* | Hypothetical protein | Wongtrakoongate et. al. | 2012 | Microbiol Immunol |
| ***68** | BPSL2863 | *dpsA* | Ferritin | Lumjiaktase et. al. | 2006 | Microbiology |
| **69** | BPSS1993 | *mprA* | Serine metalloprotease precursor | Valade et. al. | 2004 | J. Bacteriology |

Note: Ulrich et. al.: [1]; Wongtrakoongate et. al.: [2]; Lumjiaktase et. al.: [3]; Valade et. al.: [4]

**REFERENCE**

1. Ulrich RL, Deshazer D, Brueggemann EE, Hines HB, Oyston PC, et al. (2004) Role of quorum sensing in the pathogenicity of Burkholderia pseudomallei. J Med Microbiol 53: 1053-1064.

2. Wongtrakoongate P, Tumapa S, Tungpradabkul S (2012) Regulation of a quorum sensing system by stationary phase sigma factor RpoS and their co-regulation of target genes in Burkholderia pseudomallei. Microbiol Immunol 56: 281-294.

3. Lumjiaktase P, Diggle SP, Loprasert S, Tungpradabkul S, Daykin M, et al. (2006) Quorum sensing regulates dpsA and the oxidative stress response in Burkholderia pseudomallei. Microbiology 152: 3651-3659.

4. Valade E, Thibault FM, Gauthier YP, Palencia M, Popoff MY, et al. (2004) The PmlI-PmlR quorum-sensing system in Burkholderia pseudomallei plays a key role in virulence and modulates production of the MprA protease. J Bacteriol 186: 2288-2294.
