# Supplementary material for: The Condition-Dependent Transcriptional Landscape of Burkholderia pseudomallei
Source: PLoS Genet. 2013 Sep 12;9(9):e1003795. doi: 10.1371/journal.pgen.1003795 (PMC3772027; doi:10.1371/journal.pgen.1003795)
Supplement: Table S15 — Bacterial strains and sources used in this study. (DOC) [file pgen.1003795.s023.doc]

Table S15. Bacterial strains and sources used in this study.

| **Bacterial Strains** | **Source** | **Description** | **Remarks** |
| --- | --- | --- | --- |
| BpK96243 | Thailand | Clinical isolate.  Isolated in 1996 from a 34 year old female diabetic patient at Khon Kaen hospital, Northeast Thailand, with a clinical history of short incubation, septicemic infection, and rapid progression to death. | The reference strain.  Sequenced and annotated in 2004 [1] |
| Bp AL30(RpoE) | Mutant | Constructed from parental strain BpK96243.  Sigma factor mutant of which *rpoE* (*BPSL2436*) was disrupted. | [2] |
| Bp22 | Singapore | Clinical isolate.  Isolated from male in the National Service in 1989 who succumbed to the infection. | DMERI Bp strain repository,  DSO National Laboratories. |
| Bp22 *ΔbprC* | Mutant | Constructed from parental strain Bp22.  Type 3 Secretion System 3 (T3SS3) mutant;  *bprC* (*BPSS1520*) gene was deleted. | [3] |
| Bp22 *ΔbprP* | Mutant | Constructed from parental strain Bp22.  Type 3 Secretion System 3 (T3SS3) mutant;  *bprP* (*BPSS1553*) gene was deleted. | [3] |
| Bp22 *ΔbsaN* | Mutant | Constructed from parental strain Bp22.  Type 3 Secretion System 3 (T3SS3) mutant;  *bsaN* (*BPSS1546*) gene was deleted. | [3] |
| Bp22 *ΔvirAG* | Mutant | Constructed from parental strain Bp22.  Type 6 Secretion System 5 (T6SS5) double mutant;  *BPSS1494* and *BPSS1495* genes were deleted. | [3] |
| Bp008 | France | Clinical isolate.  Isolated in 1993 from a patient in France. | [4] |
| Bp008*ΔpmlI* | Mutant | Constructed from parental strain Bp008.  Quorum sensing mutant of which *pmlI* (*BPSS0885*) gene was disrupted. | [4] |

**REFERENCES**

1. Holden MT, Titball RW, Peacock SJ, Cerdeno-Tarraga AM, Atkins T, et al. (2004) Genomic plasticity of the causative agent of melioidosis, Burkholderia pseudomallei. Proc Natl Acad Sci U S A 101: 14240-14245.

2. Korbsrisate S, Vanaporn M, Kerdsuk P, Kespichayawattana W, Vattanaviboon P, et al. (2005) The Burkholderia pseudomallei RpoE (AlgU) operon is involved in environmental stress tolerance and biofilm formation. FEMS Microbiol Lett 252: 243-249.

3. Sun GW, Chen Y, Liu Y, Tan GY, Ong C, et al. (2010) Identification of a regulatory cascade controlling Type III Secretion System 3 gene expression in Burkholderia pseudomallei. Mol Microbiol 76: 677-689.

4. Valade E, Thibault FM, Gauthier YP, Palencia M, Popoff MY, et al. (2004) The PmlI-PmlR quorum-sensing system in Burkholderia pseudomallei plays a key role in virulence and modulates production of the MprA protease. J Bacteriol 186: 2288-2294.
