# Supplementary material for: The Condition-Dependent Transcriptional Landscape of Burkholderia pseudomallei
Source: PLoS Genet. 2013 Sep 12;9(9):e1003795. doi: 10.1371/journal.pgen.1003795 (PMC3772027; doi:10.1371/journal.pgen.1003795)
Supplement: Text S1 — Supplemental methods and supplemental references. (DOC) [file pgen.1003795.s024.doc]

Supplemental Material for

**The Condition-Dependent Transcriptional Landscape of *Burkholderia pseudomallei***

Wen Fong Ooi^1^*, Catherine Ong^2^*, Tannistha Nandi^1^, Jason F. Kreisberg^1^, Hui Hoon Chua^1^, Guangwen Sun^3^, Yahua Chen^4^, Claudia Mueller^5^, Laura Conejero^6^, Majid Eshaghi^1^, Roy Moh Lik Ang^1^, Jianhua Liu^1^, Bruno W. Sobral^7,8^, Sunee Korbsrisate^9^, Yunn Hwen Gan^4^, Richard W. Titball^5^, Gregory J. Bancroft^6^, Eric Valade^10,11^, Patrick Tan^1,12,13,#^

^1^Genome Institute of Singapore, Singapore, Republic of Singapore,

^2^DMERI@DSO, DSO National Laboratories, Singapore, Republic of Singapore,

^3^School of Applied Science, Republic Polytechnic, Singapore, Republic of Singapore,

^4^Department of Biochemistry, Yong Loo Lin School of Medicine, National University of Singapore, Singapore, Republic of Singapore,

^5^College of Life and Environmental Sciences, University of Exeter, Exeter, United Kingdom,

^6^Department of Immunology and Infection, Faculty of Infectious & Tropical Diseases, London School of Hygiene & Tropical Medicine, London, United Kingdom,

^7^Virginia Bioinformatics Institute at Virginia Tech, Blacksburg, United States of America,

^8^Current address: Nestle Institute of Health Sciences, Campus EPFL, Lausanne, Switzerland,

^9^Department of Immunology, Faculty of Medicine Siriraj Hospital, Mahidol University, Bangkok, Thailand,

^10^Institut de Recherche Biomédicale des Armées / CRSSA, La Tronche, France,

^11^Ecole du Val-de-Grâce, Paris, France,

^12^Duke-NUS Graduate Medical School, Singapore, Republic of Singapore,

^13^Cancer Science Institute of Singapore, National University of Singapore, Singapore, Republic of Singapore

* Equal Contribution

# Address Correspondence to [tanbop@gis.a-star.edu.sg](mailto:tanbop@gis.a-star.edu.sg)

Genome Institute of Singapore, 60 Biopolis Street, Genome, Singapore 138672

Telephone #: +65-64788182

Fax #: +65-64789003

**The file includes:**

**Supplemental Methods**

- *Identification of TARs*
- *Experimental validation of transcripts*
- *Enrichment analysis of ‘silent’ genes*
- *Relative expression of genes and ncRNAs*
- *Differential expression analysis*
- *Evaluating the significance of overlap between detectible proteins and transcripts*
- *Optimizing the clustering granularity*
- *Correlations between ncRNAs and clusters*
- *Transforming the weighted coexpression network for approximating a scale-free Network*
- *Clustering of conditions based on condition-dependent profiles*
- *Motif confirmation by BioProspector*
- *Computing network distance between disrupted genes in T3SS3 and other differentially expressed genes*
- *Computing the weighted clustering coefficients*
- *Motility assays*
- *Bp capsule electron microscopy*
- *Identifying in-vitro conditions showing similar expression profiles to mice lung infection*

**Supplemental References**

**SUPPLEMENTAL METHODS**

***Identification of TARs***

We performed TAR identification using a moving window binomial approach [1]. Briefly, the probe intensity data for each condition was log transformed to follow a Gaussian distribution. We defined a base threshold as *c* fold MAD (median absolute deviation) and the probability, $p$that a single probe passes the base threshold as:

$$p=\frac{\#\{x_{i}|x_{i}\geq cMAD\}}{n}$$

where $x_{i}$ (*i* = 1,…., *n*) = median centered log transformed data for probe *i*, and n = total number of probes. The region $\{x_{i}\}_{i-w}^{i+w}$represents the binomial sequence with signal probability$p$for each, where *w* is the predefined half window size. $p_{w}(x_{i})$was defined as the probability that $x_{i}$ is classified as signal within the signal region $\{x_{i}\}_{i-w}^{i+w}$. A region $\{x_{i}\}_{start}^{end}$was defined as a signal region if

$$\left\{\begin{matrix} p_{w}(x_{i})<\alpha,(start\leq i\leq end) \\ end-start\geq4 \\ x_{start}\geq cMAD,x_{end}\geq cMAD \end{matrix}\right.$$

where *α* is the *p*-value cutoff of the binomial test. Parameters were optimized against known TARs, leading to selection of $c=0.5$, and for further analysis.

***Experimental validation of transcripts***

Transcribed regions were PCR-amplified using a MJ Research DNA engine Dyad Peltier thermocycler (Bio-Rad Laboratories, USA) in 100ul reactions containing 5 units of Platinum Taq DNA polymerase (Invitrogen, USA), 200uM dNTPs, 2mM MgCl_2_, 200nM primers, and 2ul template cDNA. Antisense transcripts were validated using strand-specific PCR. For data analysis, the absolute value of the Ct difference was plotted, between either the Forward or Reverse primers, and the RT reaction lacking any primers.

***Enrichment analysis of ‘silent’ genes***

We evaluated the significance of overlap between 468 ‘silent’ Sanger genes and (a) 2,040 genes coding for hypothetical proteins, (b) 1,346 genes not conserved in other *B. pseudomallei* strains by performing a hypergeometric test.

***Relative expression of genes and ncRNAs***

We calculated the relative expression, of each gene/ncRNA, $x$in a condition,$s$as:

$$R_{s}=(x-\mathop{S}\limits^{-} )/MAD(S)$$

where is the gene/ncRNA expression from all conditions; $\mathop{S}\limits^{-}$is the average expression; MAD is a function of computing the median absolute deviation.

***Differential expression analysis***

Changes in expression under one condition (denoted as T; e.g. pH 4) with respect to another condition (denoted as R; e.g. pH 7) were quantified in fold change and were calculated as follows [2]: Firstly, each condition was normalized to a common reference. The common reference (R^c^) in our study is Bp K96243 grown to stationary phase in rich media (Luria Bertani broth, K9LBS). All common references were labeled with Cy5. Log-transformed fold changes (a.k.a. log ratio) were then computed as the difference between the two normalized values: $\log(T/R)=\log(T/R^{c})-\log(R/R^{c})$. The absolute fold change is the untransformed log ratio. Genes or ncRNAs with absolute fold changes > 2 were considered biologically significant.

***Evaluating the significance of overlap between detectible proteins and transcripts***

We evaluated the significance of overlap between detectible proteins and transcripts at early stationary phase [3] using a randomization procedure. A new set of detectible proteins was created through random selection from all protein-coding genes. The new set of proteins was then compared with the detectible transcripts. The steps were iteratively performed for 10,000 times, and the empirical p-value was then computed.

***Optimizing the clustering granularity***

Granularity of clustering was optimized against two parameters: (a) structural efficiency and (b) biological and functional coherence. Structurally efficient clustering, which achieves balance between the density and the size of clusters, was measured by using the tool, CLMINFO that is part of the MCL package [4]. On the other hand, functional coherence of each cluster $i$, $fc_{i}$ was measured by the fraction of gene pairs sharing identical Riley functional categories [5], $fp_{i}$:

$$fc_{i}=\frac{fp_{i}}{p_{i}}$$

given the *i*th cluster contains of annotated gene pairs. We assessed the functional coherence of clusters containing at least 60% of the interactions annotated in Riley functional categories, *n*. The functional coherence score generated from every cluster was summarized using the average coherence, :

$$A_{c}=\frac{\sum_{i} fc_{i}}{n}$$

The scores for efficiency and coherence were normalized and compared. The optimal granularity was determined as the function of maximizing the scores of the two properties.

***Correlations between ncRNAs and clusters***

We measured mean Pearson’s correlations between all pairs of ncRNAs and the expression clusters. The summarized correlation values were tested for significance using a Z-test, under the assumption that the correlations are normally distributed.

***Transforming the weighted coexpression network for approximating a scale-free Network***

We approximated the weighted coexpression network as a scale-free network by applying the fitting index$R^{2}$on the linear regression of a spectrum of weights, $s$and corresponding density, $p(s)$ in the logarithmic scale [6]. The $R^{2}$in a perfect scale-free network is 1.0. So, the $R^{2}$in the coexpression network was optimized for being greater than 0.9. Therefore, the weights between any two genes, $x_{i}$and $x_{j}$were iteratively transformed using a soft power function, $a_{ij}=MIS(x_{i},x_{j})^{\beta}$ with increasing values. The iteration stopped at $\beta=10$when $R^{2}>0.9$, thus formed an approximate scale-free network satisfying a power law: $p(s)\sim s^{-2.19}$.

***Clustering of conditions based on condition-dependent profiles***

We performed hierarchical clustering on the condition-dependent profiles using the *hclust* function in R, using the “average” linkage method and Pearson’s correlation as the similarity measure, which was subsequently modified to dissimilarity by subtracting from 1. To assess the significance of clustering, bootstrap assessment was conducted with varying fractions of genes (multiscale, 50% to 140%) using Pvclust [7]. Groups of conditions with $p\leq0.005$were considered statistically robust.

***Motif confirmation by BioProspector***

All motifs identified by MEME [8] were confirmed using BioProspector [9]. BioProspector parameters included (i) motif width corresponds to the MEME-discovered motif width, (ii) the motif may occur on only some input sequences, and (iii) single strand motif search.

***Computing network distance between disrupted genes in T3SS3 and other differentially expressed genes***

Two mutants were constructed by knocking out *bsaN* (*BPSS1546*) and *bprC* (*BPSS1520*). Genes with altered expression greater than two fold as a consequence were identified. Their distances from the mutated gene were calculated based on the transformed co-expression MIS. To determine if the observed distances are significantly shorter than chance, a randomization procedure was conducted by measuring the distances of a set of randomly selected genes, in which the total number of genes selected is the same as the differentially expressed genes. The observed and the random distances were compared using a one-tailed Wilcoxon rank sum test. The procedure was repeated for 10, 000 times. The p-values resulted from all iterations were then corrected using Benjamini and Hochberg method.

***Computing weighted clustering coefficients***

The weighted clustering coefficient (WCC) of each gene, $i$was computed as follow [10]:

$$WCC_{i}=\frac{\sum_{i\neq k} \sum_{j\neq i,j\neq k} w_{ki}w_{ij}w_{jk}}{(\sum_{i\neq k} w_{ki})^{2}-\sum_{i\neq k} w_{ki}^{2}}$$

The weight is the power-transformation of mutual information (see “Transforming the weighted coexpression network for approximating a scale-free Network”). The WCC of the entire network was computed as the average WCC of all genes.

***Motility assays***

Motility phenotypes of wild type (Bp008) and quorum sensing mutant (Bp008::Δ*pmlI*) were determined using motility tubes (BIOMERIEUX, Marcy l’Etoile). Strains were grown during 24 hours on a TSA plate, and inoculated into tubes. Tubes were observed after 24hrs and 48hrs. Motility was assessed qualitatively by examining the circular swarm formed by growing motile cells.

***Bp capsule electron microscopy***

Electron microscopy was performed using protocols modified from Puthucheary *et al.*, 1996 [11]. Bacterial cultures were harvested from hypoosmotic broth (50mM NaCl) in enriched CO_2_ after 48 hours. Pelleted cells were washed with 0.15M cacodylate buffer, pH7.0 before fixation in 25% glutaraldehyde (Sigma, USA), 0.2M cacodylate buffer, pH 7.0 (Amersham, GE Healthcare, United Kingdom), and 0.15% (w/v) aqueous ruthenium red (Fluka, USA) solution (ratio 1:6:7) for 2hrs at room temperature. Cells were washed in 0.15M cacodylate buffer and post-fixed with 4% osmium tetroxide (EMS, Euromedex, France), 0.2M cacodylate buffer, pH7.0, and 0.15% (w/v) ruthenium red mixed together in equal volumes for 3hrs at 4°C in the dark. The preparation was then incubated in a waterbath at 40°C and cells were embedded in 3% agarose (Sigma, USA). Finally, the preparation was embedded in spur resin (EMS, Euromedex, France), double-stained with uranyl acetate and lead citrate (EMS, Euromedex, France), prior to transmission electron microscopy using a JEOL JEM1010 (Japan) microscope.

***Identifying the in-vitro conditions showing similar expression profiles to mice lung infection***

The similarity of mice lung infection profile to other *in vitro* physio-chemical profiles (also known as a reference database) was determined by using a nonparametric, rank-based pattern matching strategy based on the Kolmogorov-Smirnov statistic [12]. The analysis began with a “query signature” and assessed its similarity to each expression profile in the reference database. The query signature is a list of genes with altered expression in mice lung infection. Detailed implementation is reported in [12].

**SUPPLEMENTAL REFERENCES**

1. Li J, Zhu L, Eshaghi M, Liu J, Karuturi KM (2011) Deciphering transcription factor binding patterns from genome-wide high density ChIP-chip tiling array data. BMC Proc 5 Suppl 2: S8.

2. Yang YH, Speed T (2002) Design issues for cDNA microarray experiments. Nat Rev Genet 3: 579-588.

3. Wongtrakoongate P, Roytrakul S, Yasothornsrikul S, Tungpradabkul S (2011) A proteome reference map of the causative agent of melioidosis Burkholderia pseudomallei. J Biomed Biotechnol 2011: 530926.

4. Enright AJ, Van Dongen S, Ouzounis CA (2002) An efficient algorithm for large-scale detection of protein families. Nucleic Acids Res 30: 1575-1584.

5. Wuchty S, Ipsaro JJ (2007) A draft of protein interactions in the malaria parasite P. falciparum. J Proteome Res 6: 1461-1470.

6. Min JL, Nicholson G, Halgrimsdottir I, Almstrup K, Petri A, et al. (2012) Coexpression network analysis in abdominal and gluteal adipose tissue reveals regulatory genetic loci for metabolic syndrome and related phenotypes. PLoS Genet 8: e1002505.

7. Suzuki R, Shimodaira H (2006) Pvclust: an R package for assessing the uncertainty in hierarchical clustering. Bioinformatics 22: 1540-1542.

8. Bailey TL, Elkan C (1994) Fitting a mixture model by expectation maximization to discover motifs in biopolymers. Proc Int Conf Intell Syst Mol Biol 2: 28-36.

9. Liu X, Brutlag DL, Liu JS (2001) BioProspector: discovering conserved DNA motifs in upstream regulatory regions of co-expressed genes. Pac Symp Biocomput: 127-138.

10. Zhang B, Horvath S (2005) A general framework for weighted gene co-expression network analysis. Stat Appl Genet Mol Biol 4: Article17.

11. Puthucheary SD, Vadivelu J, Ce-Cile C, Kum-Thong W, Ismail G (1996) Short report: Electron microscopic demonstration of extracellular structure of Burkholderia pseudomallei. Am J Trop Med Hyg 54: 313-314.

12. Lamb J, Crawford ED, Peck D, Modell JW, Blat IC, et al. (2006) The Connectivity Map: using gene-expression signatures to connect small molecules, genes, and disease. Science 313: 1929-1935.
